# Modifying (M)CoVaR and constructing tail risk networks through analytic higher-order moments: Evidence from the global forex markets

**DOI:** 10.1371/journal.pone.0277756

**Published:** 2022-11-29

**Authors:** Arief Hakim, A. N. M. Salman, Yeva Ashari, Khreshna Syuhada

**Affiliations:** Faculty of Mathematics and Natural Sciences, Institut Teknologi Bandung, Bandung, Indonesia; Universidad de Almeria, SPAIN

## Abstract

In a financial system, entities (e.g., companies or markets) face systemic risk that could lead to financial instability. To prevent this impact, we require quantitative systemic risk management we can carry out using conditional value-at-risk (CoVaR) and a network model. The former measures any targeted entity’s tail risk conditional on another entity being financially distressed; the latter represents the financial system through a set of nodes and a set of edges. In this study, we modify CoVaR along with its multivariate extension (MCoVaR) considering the joint conditioning events of multiple entities. We accomplish this by first employing a multivariate Johnson’s SU risk model to capture the asymmetry and leptokurticity of the entities’ asset returns. We then adopt the Cornish–Fisher expansion to account for the analytic higher-order conditional moments in modifying (M)CoVaR. In addition, we attempt to construct a conditional tail risk network. We identify its edges using a corresponding Delta (M)CoVaR reflecting the systemic risk contribution and further compute the strength and clustering coefficient of its nodes. When applying the financial system to global foreign exchange (forex) markets before and during COVID-19, we revealed that the resulting expanded (M)CoVaR forecast exhibited a better conditional coverage performance than its unexpanded version. Its superior performance appeared to be more evident over the COVID-19 period. Furthermore, our network analysis shows that advanced and emerging forex markets generally play roles as net transmitters and net receivers of systemic risk, respectively. The former (respectively, the latter) also possessed a high tendency to cluster with their neighbors in the network during (respectively, before) COVID-19. Overall, the interconnectedness and clustering tendency of the examined global forex markets substantially increased as the pandemic progressed.

## Introduction

Entities (e.g., institutions or markets) encounter financial risks arising from their own economic activities. We must manage these risks quantitatively to prevent potential losses. We can do this by employing the widely used measure of risk, namely, value-at-risk (VaR). Basically, VaR is a probability-based tail risk measure we define as the quantile of the risk or return model at a given level of confidence. One commonly assumes the model to be normally distributed to simplify the VaR calculation. This classical assumption, however, neglects evidence that the entities’ asset returns are asymmetric and leptokurtic. This evidence has led numerous studies to utilize an alternative model distributed according to the well-known (skewed) Student’s *t* distribution, but its moments may not exist for higher orders. Accordingly, several authors, e.g., [1–3], preferred to make use of Johnson’s SU model, initially introduced by Johnson [4]. In addition to being capable of capturing the asymmetry and leptokurticity of the returns, Johnson’s SU model has an uncomplicated distribution function and finite moments for all orders with explicit expressions. This is because Johnson [4] derived it from a normal model through a transformation. Nevertheless, the direct utilization of the above skewed and heavy-tailed models does not explicitly involve the skewness and kurtosis of the returns in formulating VaR. Alternatively, one can apply the asymptotic Cornish–Fisher expansion [5] to modify a VaR by accounting for these higher-order moments, as in [2, 6–11]. Specifically, this method analytically expands the quantile of the standardized return distribution around the standardized normal quantile to make the return moments higher than the second order explicitly appear. This framework is in line with investor preferences for higher-order moments [11].

When computing the VaR of a single entity’s risk, one assumes that this entity is in isolation. In practice, all entities form a financial system in which they interact with each other due to mutual economic relationships at the regional or global level. This implies that all entities’ risks could be interdependent, particularly when they are in stressful financial circumstances. As a result, illiquidity, insolvency, and losses could rapidly spread in these unstable periods and could threaten the stability of the financial system [12]. This is what we call systemic risk. One of the phenomena emblematic of systemic risk was the bankruptcy of Lehman Brothers in the United States in September 2008, which led to the global financial crisis, followed by the European sovereign debt crisis at the end of 2009 [13]. The quick propagation of these crises across global financial markets motivated us to investigate ways to properly manage systemic risk and deeply look into its transmission mechanism.

In general, systemic risk management requires two approaches. The first one utilizes a network to describe the topological structure of the entire financial system, and the other one employs a systemic risk measure that takes the influence or the contribution of financial entities into consideration [14]. Network models play a crucial role in managing systemic risk because they enable us to capture the interconnectedness among the financial entities that could trigger the formation of contagion channels, which could lead to amplifying the shocks to the financial system [15]. In the network models, we represent all the entities using nodes, and each edge connecting two nodes reflects the linkage between the corresponding entities. One can identify the existence of this linkage using numerous indicators. Some of them include the well-known Pearson’s correlation coefficient [16–21], partial correlation coefficient [22–24], and tail dependence coefficient [25–28]. However, since these coefficients only measure pairwise dependencies, they cannot accommodate directional causality relationships or measure the magnitude of the impacts [15]. This drawback makes them of limited value from an economic perspective [29]. Furthermore, Schweitzer et al. [30] noted that the linkages are not just binary (i.e., existing or not existing) but are weighted due to the mutual economic interactions. To overcome the shortcomings of correlation-based networks, previous studies have introduced some alternatives that include, for instance, the Granger causality network of Billio et al. [12] and the extreme risk spillover network of Wang et al. [31]. They constructed these networks based on the significance of the Granger-causal linkages between entities in terms of mean spillovers and extreme risk spillovers. Diebold and Yılmaz [29] developed a volatility spillover network that supplied information about the causal linkages through a generalized variance decomposition of a vector autoregression (VAR) model. Sandoval [32] and Bekiros et al. [15] proposed other networks based on a transfer entropy framework to reveal the directional transfer of information between entities’ asset returns. Recently, Wang et al. [33] introduced a trilayer information spillover network. They determined its first, second, and third layers using Granger-causality tests for mean, volatility, and risk, respectively. Wang et al. [34] built another trilayer information spillover network consisting of a return spillover layer, a volatility spillover layer, and an extreme risk spillover layer. They formed these layers based on the spillover index of Diebold and Yılmaz [29] and the least absolute shrinkage and selection operator (LASSO) method.

To measure systemic risk through the second approach, Adrian and Brunnermeier [35] introduced a conditional tail risk measure for systemic risk, namely, conditional VaR (henceforth, CoVaR). They defined this measure as the VaR of the risk a targeted entity (*j*) faces, given that another entity (*i*) experiences an extreme or systemic event. This event indicates that the latter’s risk reaches its VaR. Adrian and Brunnermeier [35] then quantified the contribution of entity *i* to the increase or the decrease in entity *j*’s risk using the difference between 1) the CoVaR if entity *i* experiences the systemic event and 2) the CoVaR if it is in a normal condition. They called this difference the ΔCoVaR. Hautsch et al. [36] and Härdle et al. [37] employed the CoVaR to construct directed and weighted tail risk networks. Meanwhile, Adrian and Brunnermeier [35] argued that the ΔCoVaR could determine network construction.

In practice, all the financial entities (except for the targeted entity) in the financial system may simultaneously experience a systemic event or a normal condition. This possibility inspired Cao [38] and Bernardi and Petrella [39] to extend (Δ)CoVaR to become (Δ)MCoVaR, where the “M” stands for multiple or multivariate. In particular, Torri et al. [40] proposed the MCoVaR of targeted entity *j*’s risk, given that entity *i* experiences a systemic event and, at the same time, other entities (except for entities *i* and *j*) are in a normal condition. Using the corresponding ΔMCoVaR, they quantified the contribution of entity *i* to entity *j* by simultaneously considering the normal conditions of the remaining entities. This framework is contrary to ΔCoVaR, which takes no account of these simultaneous conditions. The superiority of ΔMCoVaR became the basis for Torri et al. [40] to build a more representative conditional tail risk network.

In this study, we aim to modify (Δ)CoVaR and (Δ)MCoVaR by taking into account analytic higher-order conditional moments, including conditional skewness and conditional kurtosis. To achieve this aim, we first consider a multivariate Johnson’s SU model, introduced by Choi and Nam [1], for the returns of entities’ financial assets. It is a multivariate extension of Johnson’s SU model, whose moments exist for all orders with explicit expressions. We then adopt the Cornish–Fisher expansion to formulate modified versions of (Δ)CoVaR and (Δ)MCoVaR. We assess their forecast accuracy in terms of conditional coverage probabilities. In addition, we aim to construct a conditional tail risk network based on the resulting modified Δ(M)CoVaR. We then combine the network topology measures with the modified Δ(M)CoVaR forecasts. We employ them to identify central entities acting as systemically important risk transmitters and recipients and investigate the tendency of neighboring entities to make clusters together in the risk transmission mechanism.

Previous studies have used systemic risk management for stock markets at the institutional level [12, 29, 31–37, 41] and even for the indices of different countries [42–49]. However, managing systemic risk in foreign exchange (forex) markets remains an unexplored topic. Compared to other financial markets, Wang et al. [50] highlighted that forex markets possess the largest trading volumes and bridge the economy and trade of one country with those of other countries. Thus, they affect the balance of the international payment system and the development of each country’s economy. This suggests that the forex markets play a vital role in global financial security and stability. There is well-known evidence that advanced economies, such as the Eurozone, Japan, the United Kingdom, Australia, Canada, and Switzerland, dominate the global forex market. Greenwood-Nimmo et al. [51] and Polat [52] have managed systemic risk in their forex markets using an extension of Diebold and Yılmaz’s [29] spillover network. Nevertheless, Li et al. [47] and the references therein argued that risk transmissions or spillovers commonly occur in an integrated economic system, and global financial instability mainly comes from both the emerging and advanced economies. Although the forex markets of the advanced economies have large dominance, it is still important to include the forex markets of the emerging economies in systemic risk management. Bouri et al. [53] and Anwer et al. [54] have taken this notion into consideration by adopting the extended volatility spillover network, but they focused only on the Asian-Pacific forex markets. To obtain a complete picture of the tail risk interconnectedness across all the global forex markets, we attempt to apply our proposed method to the forex markets of the advanced and emerging economies worldwide. More specifically, we select 1) the five largest forex markets and 2) the forex markets of the BRICS member countries (i.e., Brazil, Russia, India, China, and South Africa). For our empirical study, we use the daily forex rates of the currencies (against the US dollar) traded in these markets during the years 2018–2021, which covers the global COVID-19 outbreak period.

In summary, the contributions of this study to the extant literature are the following. 1) This study is the first attempt to analytically modify (Δ)CoVaR and (Δ)MCoVaR as explicit functions of higher-order conditional moments through the Cornish–Fisher expansion. 2) We combine the resulting modified version of Δ(M)CoVaR with the network method to build a more sophisticated conditional tail risk network. 3) We also consider formulating several measures of the topological structure by accounting for the edge weights we determine using the modified Δ(M)CoVaR. Our conditional tail risk network is similar to that of Torri et al. [40] and Chen et al. [55]. They, however, relied on a (regression) model under the normality or Student’s *t* distributional assumption, with an application to the European banking system or the Chinese financial system. 4) In addition to providing theoretical results, we also contribute in practical terms by applying these results to the global forex markets of advanced and emerging countries before and during the COVID-19 period. This application to global forex markets is closely related to the works of Wang et al. [19], Wang and Xie [25], and Wen and Wang [56]. Nonetheless, they focused solely on utilizing a network approach on the basis of a correlation coefficient or Diebold and Yılmaz’s [29] volatility spillover index, which is different from our method.

By implementing our proposed modification framework to the aforementioned global forex markets, we found that the forecasted value of the modified (M)CoVaR had a higher accuracy, which was more pronounced during times of COVID-19. This more accurate conditional tail risk measure forecast and the resulting conditional tail risk network are thus helpful for policy-makers and investors. More specifically, these may help the former to better manage systemic risk in global forex markets and the latter to better design forex trading strategies.

## Materials and methods

### Risk models

Suppose that a financial system consists of *I* entities for a fixed positive integer *I* ≥ 2. Suppose also that ℐ={1,2,…,I} denotes a set of entities’ labels and that $X={\left(X_{i}\right)}_{i\in ℐ}$ symbolizes a random vector having a joint distribution function *F*_**θ**_ with a parameter **θ**. For each i∈ℐ, the entry *X*_*i*_ represents entity *i*’s risk, with a marginal distribution function $F_{i;\theta_{i}}$ we parameterize by **θ**_*i*_. More specifically, this random risk refers to the return of the corresponding entity’s financial asset over a given time horizon, say one day. If the expectation $E({\left|X_{i}\right|}^{2})$ is finite, we can represent *X*_*i*_ as follows:

$$\begin{array}{c} X_{i}=\mu_{i}+\sqrt{\sigma_{i}^{2}}\;Z_{i}, \end{array}$$ (1)

where $\mu_{i}=E(X_{i})\in ℜ$ and $\sigma_{i}^{2}=V(X_{i})\in {ℜ}_{+}$ denote the mean and variance of *X*_*i*_, respectively, and *Z*_*i*_ is a random variable with a standardized probability distribution.

#### Normal model

We can assume that $Z={\left(Z_{i}\right)}_{i\in ℐ}$ follows a standardized *I*-variate normal distribution, with a joint distribution function *Φ*_**P**_ we characterize by a correlation matrix $P={\left(\rho_{ij}\right)}_{i,j\in ℐ}\in {\left(-1,1\right)}^{I\times I}$. This assumption implies that **X** obeys an *I*-variate normal distribution, with a parameter **θ** = (**μ**, **Σ**), that is, $X∼N_{I}(\mu ,\Sigma )$, where $\mu ={\left(\mu_{i}\right)}_{i\in ℐ}$ denotes its mean vector, and **Σ** = **DPD** symbolizes its covariance matrix that is symmetric and nonsingular, with $D=diag{\left\{\sqrt{\sigma_{i}^{2}}\right\}}_{i\in ℐ}$. We can express its joint distribution function as follows:

$$\begin{array}{c} {}^{N}F_{\theta }\left(x\right)=\Phi_{P}\;[{\left(\frac{x_{i}-\mu_{i}}{\sqrt{\sigma_{i}^{2}}}\right)}_{i\in I}],\;x={\left(x_{i}\right)}_{i\in I}\in R^{I}. \end{array}$$

Since the correlation coefficient is invariant under any increasing linear transformation, the correlation matrix **P** of **Z** is equal to the correlation matrix of **X**. Furthermore, *X*_*i*_ possesses a skewness of *ξ*_*i*_ = 0 and a kurtosis of *κ*_*i*_ = 3, implying that it is incapable of capturing the asymmetry and leptokurticity of the financial asset returns.

#### Johnson’s SU model

As an alternative, we consider a set of transformations:

$$\begin{array}{c} Z_{i}=\zeta_{i}+\lambda_{i}\;\text{sinh}\;(\frac{Y_{i}-\gamma_{i}}{\delta_{i}}),\;i\in I, \end{array}$$ (2)

where $\gamma ={\left(\gamma_{i}\right)}_{i\in ℐ},\zeta ={\left(\zeta_{i}\right)}_{i\in ℐ}\in {ℜ}^{I}$ and $\delta ={\left(\delta_{i}\right)}_{i\in ℐ},\lambda ={\left(\lambda_{i}\right)}_{i\in ℐ}\in {ℜ}_{+}^{I}$ are vectors of constants, $Y={\left(Y_{i}\right)}_{i\in ℐ}$ is a random vector following $N_{I}(0,P)$, and sinh is the hyperbolic sine function, that is, $\text{sinh}(a)=\frac{1}{2}(e^{a}-e^{-a})$. We say that the random vector $Z={\left(Z_{i}\right)}_{i\in ℐ}$ has an *I*-variate Johnson’s SU distribution, with parameters **γ**, **δ**, **ζ**, **λ**, and **P** [1, 4, 57]. Johnson’s SU distribution belongs to the class of skewed and heavy-tailed distributions. For each i∈ℐ, the shape parameters *γ*_*i*_ and *δ*_*i*_ control its skewness and heavy-tailedness, respectively [1]. Compared to other skewed and heavy-tailed distributions, this distribution has the advantage of having a finite moment for each order *n* with the following explicit expression:

E(Zin)=∑k=0n(nk)ζik(12λi)n-k∑ℓ=0n-k(-1)n-k-ℓe(n-k-2ℓ)γiδi+12(n-k-2ℓδi)2. (3)

In particular, we can write its mean and variance as follows:

$$\begin{array}{cc} E(Z_{i}) & =\zeta_{i}-\lambda_{i}\;e^{\frac{1}{2\delta_{i}^{2}}}\text{sinh}\;(\frac{\gamma_{i}}{\delta_{i}}), \end{array}$$ (4)

$$\begin{array}{cc} V(Z_{i}) & =\frac{1}{2}\lambda_{i}^{2}(e^{\frac{1}{\delta_{i}^{2}}}-1)\;[e^{\frac{1}{\delta_{i}^{2}}}\text{cosh}\;(2\frac{\gamma_{i}}{\delta_{i}})+1], \end{array}$$ (5)

where $\text{cosh}(a)=\frac{1}{2}(e^{a}+e^{-a})$. To obtain the standardized form of Johnson’s SU distribution, we set

$$\begin{array}{cc} \zeta_{i} & =\zeta \left(\gamma_{i},\delta_{i}\right)=\lambda \left(\gamma_{i},\delta_{i}\right)\;e^{\frac{1}{2\delta_{i}^{2}}}\;\text{sinh}\;(\frac{\gamma_{i}}{\delta_{i}}), \end{array}$$ (6)

$$\begin{array}{cc} \lambda_{i} & =\lambda \left(\gamma_{i},\delta_{i}\right)={\left\{\frac{1}{2}\;(e^{\frac{1}{\delta_{i}^{2}}}-1)\;[e^{\frac{1}{\delta_{i}^{2}}}\text{cosh}\;(2\frac{\gamma_{i}}{\delta_{i}})+1]\right\}}^{-\frac{1}{2}}; \end{array}$$ (7)

Thus, we can write $Z∼J_{I}^{SU}(0,{}^{SU}P,\gamma ,\delta )$. In this case, the correlation matrix ${}^{SU}P={\left({}^{SU}\rho_{ij}\right)}_{i,j\in ℐ}$ of **Z** is different from the correlation matrix $P={\left(\rho_{ij}\right)}_{i,j\in ℐ}$ of **Y** because we derive the entries of **Z** from the entries of **Y** through the nonlinear transformation (2). For all i,j∈ℐ, we can express the entry ^SU^*ρ*_*ij*_ of ^SU^**P** as follows:

$$\begin{array}{cc} {}^{\text{SU}}\rho_{ij} & =\frac{1}{2}\lambda \left(\gamma_{i},\delta_{i}\right)\;\lambda \left(\gamma_{j},\delta_{j}\right)\;e^{\frac{1}{2(\delta_{i}^{2}+\delta_{j}^{2})}}\\ & \;\times [(e^{\frac{\rho_{ij}}{\delta_{i}\delta_{j}}}-1)\text{cosh}\;(\frac{\gamma_{i}}{\delta_{i}}+\frac{\gamma_{j}}{\delta_{j}})-(e^{-\frac{\rho_{ij}}{\delta_{i}\delta_{j}}}-1)\text{cosh}\;(\frac{\gamma_{i}}{\delta_{i}}-\frac{\gamma_{j}}{\delta_{j}})], \end{array}$$

which is strictly increasing with respect to *ρ*_*ij*_ on (−1, 1). In particular, *ρ*_*ij*_ = 0 implies ^SU^*ρ*_*ij*_ = 0. Furthermore, if *i* = *j*, then ^SU^*ρ*_*ij*_ = 1.

Considering the above transformation, we have $X∼J_{I}^{SU}(\mu ,{}^{SU}\Sigma ,\gamma ,\delta )$, with a covariance matrix ^SU^**Σ** = **D**^SU^**PD**. Its joint distribution function possesses the following expression:

$$\begin{array}{c} {}^{\text{SU}}F_{\theta }\left(x\right)=\Phi_{P}\;[{\left(\gamma_{i}+\delta_{i}\;{\text{sinh}}^{-1}\;(\frac{\frac{x_{i}-\mu_{i}}{\sqrt{\sigma_{i}^{2}}}-\zeta \left(\gamma_{i},\delta_{i}\right)}{\lambda (\gamma_{i},\delta_{i})})\right)}_{i\in I}],\;x={\left(x_{i}\right)}_{i\in I}\in R^{I}, \end{array}$$

where ${\text{sinh}}^{-1}(b)=\text{ln}\;\left(b+\sqrt{1+b^{2}}\right)$. Furthermore, we can derive the explicit skewness and kurtosis of *X*_*i*_ as follows:

$$\begin{array}{cc} \xi_{i} & =-2{\zeta (\gamma_{i},\delta_{i})}^{3}+\frac{3}{2}\zeta \left(\gamma_{i},\delta_{i}\right)\;{\lambda (\gamma_{i},\delta_{i})}^{2}[e^{\frac{2}{\delta_{i}^{2}}}\;\text{cosh}\;(2\frac{\gamma_{i}}{\delta_{i}})-1]\\ & \;-\frac{1}{4}{\lambda (\gamma_{i},\delta_{i})}^{3}\;e^{\frac{1}{2\delta_{i}^{2}}}[e^{\frac{4}{\delta_{i}^{2}}}\;\text{sinh}\;(3\frac{\gamma_{i}}{\delta_{i}})-3\;\text{sinh}\;(\frac{\gamma_{i}}{\delta_{i}})], \end{array}$$ (8)

$$\begin{array}{cc} \kappa_{i} & =-3{\zeta (\gamma_{i},\delta_{i})}^{4}+3{\zeta (\gamma_{i},\delta_{i})}^{2}\;{\lambda (\gamma_{i},\delta_{i})}^{2}[e^{\frac{2}{\delta_{i}^{2}}}\;\text{cosh}\;(2\frac{\gamma_{i}}{\delta_{i}})-1]\\ & \;-\zeta \left(\gamma_{i},\delta_{i}\right)\;{\lambda (\gamma_{i},\delta_{i})}^{3}\;e^{\frac{1}{2\delta_{i}^{2}}}[e^{\frac{4}{\delta_{i}^{2}}}\;\text{sinh}\;(3\frac{\gamma_{i}}{\delta_{i}})-3\text{sinh}\;(\frac{\gamma_{i}}{\delta_{i}})]\\ & \;+\frac{1}{8}{\lambda (\gamma_{i},\delta_{i})}^{4}[e^{\frac{8}{\delta_{i}^{2}}}\text{cosh}\;(4\frac{\gamma_{i}}{\delta_{i}})-4e^{\frac{2}{\delta_{i}^{2}}}\text{cosh}\;(2\frac{\gamma_{i}}{\delta_{i}})+3]. \end{array}$$ (9)

### (Δ)CoVaR and its extension, (Δ)MCoVaR

For each i∈ℐ, we employ value-at-risk (VaR) to measure the risk, specifically the tail risk, of entity *i*. For a given significance level *α*_*i*_ ∈ (0, 1) commonly close to zero, e.g., 5%, we define it as a value ${VaR}_{i}^{\alpha_{i}}(\theta_{i})={VaR}^{\alpha_{i}}(X_{i};\theta_{i})$ satisfying the following coverage probability equation: $P\;\left\{-X_{i}\leq {VaR}_{i}^{\alpha_{i}}(\theta_{i})\right\}=1-\alpha_{i}$ or, equivalently, $P\;\left\{X_{i}\leq -{VaR}_{i}^{\alpha_{i}}(\theta_{i})\right\}=\alpha_{i}$. This means that ${VaR}_{i}^{\alpha_{i}}(\theta_{i})$ is actually the negative of the *α*_*i*_-quantile of the distribution of *X*_*i*_. However, we derive it by assuming that entity *i* is in isolation and disregarding the dependence between its risk and other entities’ risks.

#### (Δ)CoVaR

Given that entity *i* experiences a systemic or distressing event, i.e., $\left\{X_{i}=-{VaR}_{i}^{\alpha_{i}}(\theta_{i})\right\}$, we compute the VaR of the tail risk of another entity j∈ℐ∖{i} at a given significance level *α*_*j*_ ∈ (0, 1) that can be different from *α*_*i*_. Adrian and Brunnermeier [35] called this tail risk measure the conditional VaR (CoVaR). This means that ${CoVaR}_{j|i}^{\alpha_{j}}\;\left(\theta_{j|i}^{\alpha_{i}}\right)={VaR}^{\alpha_{j}}\;\left[X_{j}|\;\left\{X_{i}=-{VaR}_{i}^{\alpha_{i}}(\theta_{i})\right\}\;;\theta_{j|i}^{\alpha_{i}}\right]$, which has a conditional coverage probability

$$\begin{array}{c} P\;[\{X_{j}\leq -{\text{CoVaR}}_{j|i}^{\alpha_{j}}\;(\theta_{j|i}^{\alpha_{i}})\}|\;\{X_{i}=-{\text{VaR}}_{i}^{\alpha_{i}}\left(\theta_{i}\right)\}\;]=\alpha_{j}. \end{array}$$ (10)

In other words, we can determine the CoVaR using a quantile function of the conditional distribution of *X*_*j*_, given $\left\{X_{i}=-{VaR}_{i}^{\alpha_{i}}(\theta_{i})\right\}$, with a parameter $\theta_{j|i}^{\alpha_{i}}$ that depends on the significance level *α*_*i*_. We then quantify the systemic risk contribution of entity *i* to entity *j* using the difference between 1) the CoVaR we calculate when entity *i* experiences the systemic event and 2) the CoVaR we compute when it is in a normal or median state, i.e.,

$$\begin{array}{c} \Delta {\text{CoVaR}}_{j|i}^{\alpha_{j}}\;(\theta_{j|i}^{\alpha_{i}},\theta_{j|i}^{50\%})={\text{CoVaR}}_{j|i}^{\alpha_{j}}\;(\theta_{j|i}^{\alpha_{i}})-{\text{CoVaR}}_{j|i}^{\alpha_{j}}\;(\theta_{j|i}^{50\%}). \end{array}$$ (11)

In other words, the above ΔCoVaR measures the change in the tail risk of entity *j* when entity *i* moves from a nondistressing event to a distressing event. Its positive value indicates that the distress that entity *i* experiences results in an increase in entity *j*’s tail risk, suggesting the presence of a tail risk transmission from entity *i* to entity *j*.

Under the normality assumption, we can easily verify that ${}^{N}{VaR}_{i}^{\alpha_{i}}\;\left(\mu_{i},\sigma_{i}^{2}\right)=-\mu_{i}-\sqrt{\sigma_{i}^{2}}\;z^{\alpha_{i}}$, with $z^{\alpha_{i}}=\Phi^{-1}(\alpha_{i})$ denoting the *α*_*i*_-quantile of the standardized univariate normal distribution. Furthermore, the conditional distribution of $X_{j}|\;\left\{X_{i}=-{}^{N}{VaR}_{i}^{\alpha_{i}}\;\left(\mu_{i},\sigma_{i}^{2}\right)\right\}$ is $N\;\left(\mu_{j|i}^{\alpha_{i}},\sigma_{j|i}^{2;\alpha_{i}}\right)$, with $\mu_{j|i}^{\alpha_{i}}=\mu_{j}+\sqrt{\sigma_{j}^{2}}\;\rho_{ij}\;z^{\alpha_{i}}$ and $\sigma_{j|i}^{2;\alpha_{i}}=\sigma_{j}^{2}\;\left(1-\rho_{ij}^{2}\right)$. As a result, we can formulate its CoVaR as follows:

$$\begin{array}{cc} {}^{N}{\text{CoVaR}}_{j|i}^{\alpha_{j}}\;(\mu_{j|i}^{\alpha_{i}},\sigma_{j|i}^{2;\alpha_{i}}) & =-\mu_{j|i}^{\alpha_{i}}-\sqrt{\sigma_{j|i}^{2;\alpha_{i}}}\;z^{\alpha_{j}}\\ & =-\mu_{j}-\sqrt{\sigma_{j}^{2}}\;\rho_{ij}\;z^{\alpha_{i}}-\sqrt{\sigma_{j}^{2}\;(1-\rho_{ij}^{2})}\;z^{\alpha_{j}}. \end{array}$$ (12)

We can then derive the corresponding ΔCoVaR as follows:

$$\begin{array}{c} \Delta {}^{N}{\text{CoVaR}}_{j|i}^{\alpha_{j}}\;(\theta_{j|i}^{\alpha_{i}},\theta_{j|i}^{50\%})=-\sqrt{\sigma_{j}^{2}}\;\rho_{ij}\;z^{\alpha_{i}}, \end{array}$$ (13)

which does not depend on the significance level *α*_*j*_ we set for targeted entity *j*.

When assuming Johnson’s SU model, we can express the VaR of *X*_*i*_ as follows: ${}^{SU}{VaR}_{i}^{\alpha_{i}}\;\left(\mu_{i},\sigma_{i}^{2},\gamma_{i},\delta_{i}\right)=-\mu_{i}-\sqrt{\sigma_{i}^{2}}\;{}^{SU}z^{\alpha_{i}}(\gamma_{i},\delta_{i})$, where according to Eq (2), ${}^{SU}z^{\alpha_{i}}(\gamma_{i},\delta_{i})=\zeta (\gamma_{i},\delta_{i})+\lambda (\gamma_{i},\delta_{i})\text{sinh}\;\left(\frac{z^{\alpha_{i}}-\gamma_{i}}{\delta_{i}}\right)$ denotes the *α*_*i*_-quantile of the standardized Johnson’s SU distribution, with shape parameters *γ*_*i*_ and *δ*_*i*_. We can further derive the conditional distribution function of *X*_*j*_, given {*X*_*i*_ = *x*_*i*_}, as follows:

$$\begin{array}{cc} F_{j|i;\theta_{j|i}}(x_{j}\;|\;x_{i}) & =P(\left\{X_{j}\leq x_{j}\right\}\;|\;\{X_{i}=x_{i}\})\\ & =P(\;\{Z_{j}\leq \frac{x_{j}-\mu_{j}}{\sqrt{\sigma_{j}^{2}}}\}\;|\{Z_{i}=\frac{x_{i}-\mu_{i}}{\sqrt{\sigma_{i}^{2}}}\})\\ & =P(\left\{Y_{j}\leq y_{j}\right\}\;|\;\{Y_{i}=y_{i}\}), \end{array}$$

with $y_{k}=\gamma_{k}+\delta_{k}\;{\text{sinh}}^{-1}\;\left(\frac{\frac{x_{k}-\mu_{k}}{\sqrt{\sigma_{k}^{2}}}-\zeta (\gamma_{k},\delta_{k})}{\lambda (\gamma_{k},\delta_{k})}\right)$, *k* ∈ {*i*, *j*}. Since $Y_{j}\;|\;\{Y_{i}=y_{i}\}∼N\;\left(\rho_{ij}\;y_{i},1-\rho_{ij}^{2}\right)$, we then have

$$\begin{array}{cc} F_{j|i;\theta_{j|i}}(x_{j}\;|\;x_{i}) & =\Phi (\frac{y_{j}-\rho_{ij}\;y_{i}}{\sqrt{1-\rho_{ij}^{2}}})\\ & =\Phi \;[\frac{\gamma_{j}-\rho_{ij}\;y_{i}}{\sqrt{1-\rho_{ij}^{2}}}+\frac{\delta_{j}}{\sqrt{1-\rho_{ij}^{2}}}{\text{sinh}}^{-1}\;(\frac{\frac{x_{j}-\mu_{j}}{\sqrt{\sigma_{j}^{2}}}-\zeta \left(\gamma_{j},\delta_{j}\right)}{\lambda (\gamma_{j},\delta_{j})})]. \end{array}$$

This implies that *X*_*j*_, given $\left\{X_{i}=-{}^{SU}{VaR}_{i}^{\alpha_{i}}\;\left(\mu_{i},\sigma_{i}^{2},\gamma_{i},\delta_{i}\right)\right\}$, is distributed according to $J^{SU}\;\left(\mu_{j|i}^{\alpha_{i}},\sigma_{j|i}^{2;\alpha_{i}},\gamma_{j|i}^{\alpha_{i}},\delta_{j|i}\right)$, where following Eqs (4) and (5),

$$\begin{array}{cc} \mu_{j|i}^{\alpha_{i}} & =\mu_{j}+\sqrt{\sigma_{j}^{2}}[\zeta \left(\gamma_{j},\delta_{j}\right)-\lambda \left(\gamma_{j},\delta_{j}\right)\;e^{\frac{1}{2{\left(\delta_{j|i}\right)}^{2}}}\text{sinh}(\frac{\gamma_{j|i}^{\alpha_{i}}}{\delta_{j|i}})], \end{array}$$ (14)

$$\begin{array}{cc} \sigma_{j|i}^{2;\alpha_{i}} & =\frac{1}{2}\sigma_{j}^{2}\;{\lambda (\gamma_{j},\delta_{j})}^{2}(e^{\frac{1}{{\left(\delta_{j|i}\right)}^{2}}}-1)\;[e^{\frac{1}{{\left(\delta_{j|i}\right)}^{2}}}\text{cosh}(2\frac{\gamma_{j|i}^{\alpha_{i}}}{\delta_{j|i}})+1], \end{array}$$ (15)

with shape parameters

$$\begin{array}{c} \gamma_{j|i}^{\alpha_{i}}=\frac{\gamma_{j}-\rho_{ij}\;z^{\alpha_{i}}}{\sqrt{1-\rho_{ij}^{2}}},\;\delta_{j|i}=\frac{\delta_{j}}{\sqrt{1-\rho_{ij}^{2}}}. \end{array}$$ (16)

Consequently, we obtain

$$\begin{array}{c} {}^{\text{SU}}{\text{CoVaR}}_{j|i}^{\alpha_{j}}\;(\mu_{j|i}^{\alpha_{i}},\sigma_{j|i}^{2;\alpha_{i}},\gamma_{j|i}^{\alpha_{i}},\delta_{j|i})=-\mu_{j|i}^{\alpha_{i}}-\sqrt{\sigma_{j|i}^{2;\alpha_{i}}}\;{}^{\text{SU}}z^{\alpha_{j}}\;(\gamma_{j|i}^{\alpha_{i}},\delta_{j|i}), \end{array}$$ (17)

with ${}^{SU}z^{\alpha_{j}}\;\left(\gamma_{j|i}^{\alpha_{i}},\delta_{j|i}\right)=\zeta \;\left(\gamma_{j|i}^{\alpha_{i}},\delta_{j|i}\right)+\lambda \;\left(\gamma_{j|i}^{\alpha_{i}},\delta_{j|i}\right)\text{sinh}\;\left(\frac{z^{\alpha_{j}}-\gamma_{j|i}^{\alpha_{i}}}{\delta_{j|i}}\right)$ being the *α*_*j*_-quantile of $J^{SU}\;\left(0,1,\gamma_{j|i}^{\alpha_{i}},\delta_{j|i}\right)$. Furthermore, we derive the corresponding ΔCoVaR equal to ${}^{SU}{CoVaR}_{j|i}^{\alpha_{j}}\;\left(\mu_{j|i}^{\alpha_{i}},\sigma_{j|i}^{2;\alpha_{i}},\gamma_{j|i}^{\alpha_{i}},\delta_{j|i}\right)-{}^{SU}{CoVaR}_{j|i}^{\alpha_{j}}\;\left(\mu_{j|i}^{50\%},\sigma_{j|i}^{2;50\%},\gamma_{j|i}^{50\%},\delta_{j|i}\right)$.

#### (Δ)MCoVaR

For all i,j∈ℐ, *i* ≠ *j*, we have found that the (Δ)CoVaR of targeted entity *j*’s risk depends only on the conditioning entity *i*’s risk and takes no account of its dependence on the risks of all the remaining entities in the financial system. By including the conditioning events of all the entities, except for targeted entity *j*, we follow Torri et al. [40] to formulate a multivariate extension of (Δ)CoVaR, namely, (Δ)MCoVaR. Specifically, we define the MCoVaR as the VaR of targeted entity *j*’s risk under the condition that entity *i* experiences a systemic event and that the remaining entities are jointly in their median state. We denote it as follows:

$$\begin{array}{cc} & {\text{MCoVaR}}_{j|i,\backslash ij}^{\alpha_{j}}\;(\theta_{j|i,\backslash ij}^{\alpha_{i},50\%})\\ & ={\text{VaR}}^{\alpha_{j}}\;[X_{j}|\;\{X_{i}=-{\text{VaR}}_{i}^{\alpha_{i}}\left(\theta_{i}\right)\}⋂{\left\{X_{k}=-{\text{VaR}}_{k}^{50\%}\left(\theta_{k}\right)\right\}}_{k\in I_{\backslash ij}};\theta_{j|i,\backslash ij}^{\alpha_{i},50\%}], \end{array}$$ (18)

where ∖ij=ℐ∖{i,j}. We then define the corresponding ΔMCoVaR as the difference ${MCoVaR}_{j|i,\setminus ij}^{\alpha_{j}}\;\left(\theta_{j|i,\setminus ij}^{\alpha_{i},50\%}\right)-{MCoVaR}_{j|i,\setminus ij}^{\alpha_{j}}\;\left(\theta_{j|i,\setminus ij}^{50\%,50\%}\right)$.

Under the normality assumption, rearranging the entries of $Z∼N_{I}(0,P)$ results in

$$\begin{array}{c} (\begin{array}{c} Z_{j}\\ Z_{\backslash j} \end{array})∼N_{I}\;[(\begin{array}{c} 0\\ 0 \end{array}),(\begin{array}{cc} 1 & P_{j,\backslash j}\\ P_{\backslash j,j} & P_{\backslash j,\backslash j} \end{array})], \end{array}$$ (19)

where **P**_∖*j*, ∖*j*_ is the correlation matrix of $Z_{\setminus j}={\left(Z_{k}\right)}_{k\in {ℐ}_{\setminus j}}$, and the row matrix $P_{j,\setminus j}=P_{\setminus j,j}^{⊤}$ consists of the correlation coefficients between *Z*_*j*_ and all the entries of **Z**_∖*j*_. Accordingly, for all $z_{\setminus j}\in {ℜ}^{I-1}$, we have

$$\begin{array}{c} Z_{j}|\;\{Z_{\backslash j}=z_{\backslash j}\}\;∼N\;(P_{j,\backslash j}\;P_{\backslash j,\backslash j}^{-1}\;z_{\backslash j},1-P_{j,\backslash j}\;P_{\backslash j,\backslash j}^{-1}\;P_{\backslash j,j}). \end{array}$$

This implies that $\left(X_{j}|\;\left\{X_{\setminus j}=x_{\setminus j}\right\}\right)\overset{d}{=}\left(\;\mu_{j}+\sqrt{\sigma_{j}^{2}}\;Z_{j}\;|\left\{Z_{\setminus j}=z_{\setminus j}\right\}\right)$, which is normally distributed, with a conditional mean $\mu_{j}+\sqrt{\sigma_{j}^{2}}\;P_{j,\setminus j}\;P_{\setminus j,\setminus j}^{-1}\;z_{\setminus j}$ and a conditional variance $\sigma_{j}^{2}\;\left(1-P_{j,\setminus j}\;P_{\setminus j,\setminus j}^{-1}\;P_{\setminus j,j}\right)$, where $z_{\setminus j}={\left(\frac{x_{k}-\mu_{k}}{\sqrt{\sigma_{k}^{2}}}\right)}_{k\in {ℐ}_{\setminus j}}$. Since ${}^{N}{VaR}_{i}^{\alpha_{i}}\;\left(\mu_{i},\sigma_{i}^{2}\right)=-\mu_{i}-\sqrt{\sigma_{i}^{2}}\;z^{\alpha_{i}}$ and *z*^50%^ = *Φ*^−1^(50%) = 0, we can write the resulting MCoVaR as follows:

$$\begin{array}{cc} {}^{N}{\text{MCoVaR}}_{j|i,\backslash ij}^{\alpha_{j}}\;(\theta_{j|i,\backslash ij}^{\alpha_{i},50\%}) & =-\mu_{j}-\sqrt{\sigma_{j}^{2}}\;{\left(P_{j,\backslash j}\;P_{\backslash j,\backslash j}^{-1}\right)}_{i}\;z^{\alpha_{i}}\\ & \;-\sqrt{\sigma_{j}^{2}\;(1-P_{j,\backslash j}\;P_{\backslash j,\backslash j}^{-1}\;P_{\backslash j,j})}\;z^{\alpha_{j}}, \end{array}$$ (20)

where ${\left(P_{j,\setminus j}\;P_{\setminus j,\setminus j}^{-1}\right)}_{i}$ is the entry of the row matrix $P_{j,\setminus j}\;P_{\setminus j,\setminus j}^{-1}$ with index *i*. We can then derive the corresponding ΔMCoVaR as follows:

$$\begin{array}{c} \Delta {}^{N}{\text{MCoVaR}}_{j|i,\backslash ij}^{\alpha_{j}}\;(\theta_{j|i,\backslash ij}^{\alpha_{i},50\%},\theta_{j|i,\backslash ij}^{50\%,50\%})=-\sqrt{\sigma_{j}^{2}}\;{\left(P_{j,\backslash j}\;P_{\backslash j,\backslash j}^{-1}\right)}_{i}\;z^{\alpha_{i}}. \end{array}$$ (21)

Similarly, we can rearrange the random vector $Y∼N_{I}(0,P)$ determining Johnson’s SU transformation (2), as in Eq (19). Through this rearrangement, we find that *Y*_*j*_|{**Y**_∖*j*_ = **y**_∖*j*_} obeys a normal distribution, with a conditional mean $P_{j,\setminus j}\;P_{\setminus j,\setminus j}^{-1}\;y_{\setminus j}$ and a conditional variance $1-P_{j,\setminus j}\;P_{\setminus j,\setminus j}^{-1}\;P_{\setminus j,j}$, for all $y_{\setminus j}\in {ℜ}^{I-1}$. As a result, if **X** is distributed according to an *I*-variate Johnson’s SU distribution, the conditional distribution function of *X*_*j*_|{**X**_∖*j*_ = **x**_∖*j*_} has the following expression:

$$\begin{array}{cc} F_{j|\backslash j;\theta_{j|\backslash j}}\;(x_{j}|\;x_{\backslash j}) & =P\;(\{X_{j}\leq x_{j}\}|\;\{X_{\backslash j}=x_{\backslash j}\}\;)\\ & =P\;(\{Y_{j}\leq y_{j}\}|\;\{Y_{\backslash j}=y_{\backslash j}\}\;)\\ & =\Phi (\frac{y_{j}-P_{j,\backslash j}\;P_{\backslash j,\backslash j}^{-1}\;y_{\backslash j}}{\sqrt{1-P_{j,\backslash j}\;P_{\backslash j,\backslash j}^{-1}\;P_{\backslash j,j}}}), \end{array}$$

which is equal to

$$\begin{array}{c} \Phi \;[\frac{\gamma_{j}-P_{j,\backslash j}\;P_{\backslash j,\backslash j}^{-1}\;y_{\backslash j}}{\sqrt{1-P_{j,\backslash j}\;P_{\backslash j,\backslash j}^{-1}\;P_{\backslash j,j}}}+\frac{\delta_{j}}{\sqrt{1-P_{j,\backslash j}\;P_{\backslash j,\backslash j}^{-1}\;P_{\backslash j,j}}}\;{\text{sinh}}^{-1}\;(\frac{\frac{x_{j}-\mu_{j}}{\sqrt{\sigma_{j}^{2}}}-\zeta \left(\gamma_{j},\delta_{j}\right)}{\lambda (\gamma_{j},\delta_{j})})], \end{array}$$

where $y_{k}=\gamma_{k}+\delta_{k}{\text{sinh}}^{-1}\;\left(\frac{\frac{x_{k}-\mu_{k}}{\sqrt{\sigma_{k}^{2}}}-\zeta (\gamma_{k},\delta_{k})}{\lambda (\gamma_{k},\delta_{k})}\right)$, k∈ℐ. Since ${}^{SU}{VaR}_{i}^{\alpha_{i}}\;\left(\mu_{i},\sigma_{i}^{2},\gamma_{i},\delta_{i}\right)=-\mu_{i}-\sqrt{\sigma_{i}^{2}}\;{}^{SU}z^{\alpha_{i}}(\gamma_{i},\delta_{i})$, with ${}^{SU}z^{\alpha_{i}}(\gamma_{i},\delta_{i})=\zeta (\gamma_{i},\delta_{i})+\lambda (\gamma_{i},\delta_{i})\text{sinh}\;\left(\frac{z^{\alpha_{i}}-\gamma_{i}}{\delta_{i}}\right)$, the conditional distribution of *X*_*j*_, given both $\left\{X_{i}=-{}^{SU}{VaR}_{i}^{\alpha_{i}}\;\left(\mu_{i},\sigma_{i}^{2},\gamma_{i},\delta_{i}\right)\right\}$ and ${\left\{X_{k}=-{}^{SU}{VaR}_{k}^{50\%}\;\left(\mu_{k},\sigma_{k}^{2},\gamma_{k},\delta_{k}\right)\right\}}_{k\in {ℐ}_{\setminus ij}}$, is $J^{SU}\;\left(\mu_{j|i,\setminus ij}^{\alpha_{i},50\%},\sigma_{j|i,\setminus ij}^{2;\alpha_{i},50\%},\gamma_{j|i,\setminus ij}^{\alpha_{i},50\%},\delta_{j|i,\setminus ij}\right)$. According to Eqs (4) and (5), we can express its conditional mean and conditional variance as follows:

$$\begin{array}{cc} \mu_{j|i,\backslash ij}^{\alpha_{i},50\%} & =\mu_{j}+\sqrt{\sigma_{j}^{2}}[\zeta \left(\gamma_{j},\delta_{j}\right)-\lambda \left(\gamma_{j},\delta_{j}\right)\;e^{\frac{1}{2{\left(\delta_{j|i,\backslash ij}\right)}^{2}}}\text{sinh}(\frac{\gamma_{j|i,\backslash ij}^{\alpha_{i},50\%}}{\delta_{j|i,\backslash ij}})], \end{array}$$ (22)

$$\begin{array}{cc} \sigma_{j|i,\backslash ij}^{2;\alpha_{i},50\%} & =\frac{1}{2}\sigma_{j}^{2}\;{\lambda (\gamma_{j},\delta_{j})}^{2}(e^{\frac{1}{{\left(\delta_{j|i,\backslash ij}\right)}^{2}}}-1)\;[e^{\frac{1}{{\left(\delta_{j|i,\backslash ij}\right)}^{2}}}\text{cosh}(2\frac{\gamma_{j|i,\backslash ij}^{\alpha_{i},50\%}}{\delta_{j|i,\backslash ij}})+1], \end{array}$$ (23)

which depend on the shape parameters

$$\begin{array}{c} \gamma_{j|i,\backslash ij}^{\alpha_{i},50\%}=\frac{\gamma_{j}-{\left(P_{j,\backslash j}\;P_{\backslash j,\backslash j}^{-1}\right)}_{i}\;z^{\alpha_{i}}}{\sqrt{1-P_{j,\backslash j}\;P_{\backslash j,\backslash j}^{-1}\;P_{\backslash j,j}}},\;\delta_{j|i,\backslash ij}=\frac{\delta_{j}}{\sqrt{1-P_{j,\backslash j}\;P_{\backslash j,\backslash j}^{-1}\;P_{\backslash j,j}}}. \end{array}$$ (24)

Therefore, we derive

$$\begin{array}{cc} & {}^{\text{SU}}{\text{MCoVaR}}_{j|i,\backslash ij}^{\alpha_{j}}\;(\mu_{j|i,\backslash ij}^{\alpha_{i},50\%},\sigma_{j|i,\backslash ij}^{2;\alpha_{i},50\%},\gamma_{j|i,\backslash ij}^{\alpha_{i},50\%},\delta_{j|i,\backslash ij})\\ & =-\mu_{j|i,\backslash ij}^{\alpha_{i},50\%}-\sqrt{\sigma_{j|i,\backslash ij}^{2;\alpha_{i},50\%}}\;{}^{\text{SU}}z^{\alpha_{j}}\;(\gamma_{j|i,\backslash ij}^{\alpha_{i},50\%},\delta_{j|i,\backslash ij}), \end{array}$$ (25)

with the term ${}^{SU}z^{\alpha_{j}}\;\left(\gamma_{j|i,\setminus ij}^{\alpha_{i},50\%},\delta_{j|i,\setminus ij}\right)=\zeta \;\left(\gamma_{j|i,\setminus ij}^{\alpha_{i},50\%},\delta_{j|i,\setminus ij}\right)+\lambda \;\left(\gamma_{j|i,\setminus ij}^{\alpha_{i},50\%},\delta_{j|i,\setminus ij}\right)\text{sinh}\left(\frac{z^{\alpha_{j}}-\gamma_{j|i,\setminus ij}^{\alpha_{i},50\%}}{\delta_{j|i,\setminus ij}}\right)$ symbolizing the *α*_*j*_-quantile of $J^{SU}\;\left(0,1,\gamma_{j|i,\setminus ij}^{\alpha_{i},50\%},\delta_{j|i,\setminus ij}\right)$. We then obtain the corresponding ΔMCoVaR as follows: ${}^{SU}{MCoVaR}_{j|i,\setminus ij}^{\alpha_{j}}\;\left(\mu_{j|i,\setminus ij}^{\alpha_{i},50\%},\sigma_{j|i,\setminus ij}^{2;\alpha_{i},50\%},\gamma_{j|i,\setminus ij}^{\alpha_{i},50\%},\delta_{j|i,\setminus ij}\right)-{}^{SU}{MCoVaR}_{j|i,\setminus ij}^{\alpha_{j}}\;\left(\mu_{j|i,\setminus ij}^{50\%,50\%},\sigma_{j|i,\setminus ij}^{2;50\%,50\%},\gamma_{j|i,\setminus ij}^{50\%,50\%},\delta_{j|i,\setminus ij}\right)$.

### Modified (M)CoVaR with higher-order moments

We have shown that the CoVaR and MCoVaR under the normality assumption do not reflect the skewed and heavy-tailed returns. In addition, Johnson’s SU distributional assumption does not explicitly involve the conditional skewness and conditional kurtosis of these returns in formulating both the CoVaR and MCoVaR. To overcome these drawbacks, we propose to modify them by asymptotically expanding the quantile of the standardized conditional return model around the standardized normal quantile with the inclusion of such higher-order conditional moments.

First, consider the Edgeworth expansion method [5] that can expand the distribution function $E_{G_{i}}$ of the standardized return model $Z_{i}=\frac{X_{i}-\mu_{i}}{\sqrt{\sigma_{i}^{2}}}$ around the standardized normal distribution function *Φ* as follows:

$$\begin{array}{c} {}^{E}G_{i}\left(z\right)=\Phi \left(z\right)-\phi \left(z\right)\sum_{n=0}^{\infty }a_{n}\;H_{n}\left(z\right). \end{array}$$

The function *ϕ* is the standardized normal probability function, *a*_*n*_ is a constant, and *H*_*n*_(*z*) is a Hermite polynomial of order *n* in *z* satisfying $\frac{d^{n}}{dz^{n}}\phi (z)={\left(-1\right)}^{n}\;\phi (z)\;H_{n}(z)$ [58]. In particular, we attempt to account for its skewness *ξ*_*i*_ and kurtosis *κ*_*i*_ by considering the following expansion [5, 9]:

$$\begin{array}{c} {}^{E}G_{i}\left(z\right)=\Phi \left(z\right)-\phi \left(z\right)\;[\frac{\xi_{i}}{6}H_{2}\left(z\right)+\frac{\kappa_{i}-3}{24}H_{3}\left(z\right)+\frac{\xi_{i}^{2}}{72}H_{5}\left(z\right)], \end{array}$$

which depends on the Hermite polynomials *H*_2_(*z*) = *z*^2^−1, *H*_3_(*z*) = *z*^3^−3*z*, and *H*_5_(*z*) = *z*^5^−10*z*^3^+ 15*z* of order two, three, and five, respectively. We can then use the corresponding Cornish–Fisher (CF) expansion to expand the *α*_*i*_-quantile of $Z_{i}=\frac{X_{i}-\mu_{i}}{\sqrt{\sigma_{i}^{2}}}$ around the standardized normal *α*_*i*_-quantile $z^{\alpha_{i}}=\Phi^{-1}(\alpha_{i})$ as follows [5, 9]:

$$\begin{array}{c} {}^{\text{CF}}z^{\alpha_{i}}\left(\xi_{i},\kappa_{i}\right)=z^{\alpha_{i}}+\frac{\xi_{i}}{6}H_{2}\left(z^{\alpha_{i}}\right)+\frac{\kappa_{i}-3}{24}H_{3}\left(z^{\alpha_{i}}\right)-\frac{\xi_{i}^{2}}{36}[H_{1}\left(z^{\alpha_{i}}\right)+2H_{3}\left(z^{\alpha_{i}}\right)], \end{array}$$ (26)

with *H*_1_(*z*) = *z*.

#### Four-moment CoVaR

By making use of the abovementioned expansion method, we can expand the VaR of entity *i*’s risk we derive under Johnson’s SU distributional assumption as follows:

$$\begin{array}{c} {}^{\text{CF}}{\text{VaR}}_{i}^{\alpha_{i}}\;(\mu_{i},\sigma_{i}^{2},\xi_{i},\kappa_{i})=-\mu_{i}-\sqrt{\sigma_{i}^{2}}\;{}^{\text{CF}}z^{\alpha_{i}}\left(\xi_{i},\kappa_{i}\right), \end{array}$$ (27)

which depends on the skewness *ξ*_*i*_ and kurtosis *κ*_*i*_ in Eqs (8) and (9), respectively. Given $\left\{X_{i}=-{}^{CF}{VaR}_{i}^{\alpha_{i}}\;\left(\mu_{i},\sigma_{i}^{2},\xi_{i},\kappa_{i}\right)\right\}$, we can show that the conditional distribution of *X*_*j*_ is $J^{SU}\;\left(\mu_{j|i}^{\alpha_{i}},\sigma_{j|i}^{2;\alpha_{i}},\gamma_{j|i}^{\alpha_{i}},\delta_{j|i}\right)$. We formulate its conditional mean $\mu_{j|i}^{\alpha_{i}}$ and conditional variance $\sigma_{j|i}^{2;\alpha_{i}}$ in Eqs (14) and (15), respectively, with new shape parameters

$$\begin{array}{c} \gamma_{j|i}^{\alpha_{i}}=\frac{\gamma_{j}-\rho_{ij}\;[\gamma_{i}+\delta_{i}\;{\text{sinh}}^{-1}\;(\frac{{}^{\text{CF}}z^{\alpha_{i}}\left(\xi_{i},\kappa_{i}\right)-\zeta \left(\gamma_{i},\delta_{i}\right)}{\lambda (\gamma_{i},\delta_{i})})]}{\sqrt{1-\rho_{ij}^{2}}},\;\delta_{j|i}=\frac{\delta_{j}}{\sqrt{1-\rho_{ij}^{2}}}. \end{array}$$ (28)

By adopting the results in Eqs (8) and (9), we can explicitly express its conditional skewness and conditional kurtosis as follows:

$$\begin{array}{cc} \xi_{j|i}^{\alpha_{i}} & =-2{\zeta \;(\gamma_{j|i}^{\alpha_{i}},\delta_{j|i})}^{3}+\frac{3}{2}\zeta \;(\gamma_{j|i}^{\alpha_{i}},\delta_{j|i}){\lambda \;(\gamma_{j|i}^{\alpha_{i}},\delta_{j|i})}^{2}[e^{\frac{2}{{\left(\delta_{j|i}\right)}^{2}}}\;\text{cosh}(2\frac{\gamma_{j|i}^{\alpha_{i}}}{\delta_{j|i}})-1]\\ & \;-\frac{1}{4}{\lambda \;(\gamma_{j|i}^{\alpha_{i}},\delta_{j|i})}^{3}\;e^{\frac{1}{2{\left(\delta_{j|i}\right)}^{2}}}[e^{\frac{4}{{\left(\delta_{j|i}\right)}^{2}}}\text{sinh}(3\frac{\gamma_{j|i}^{\alpha_{i}}}{\delta_{j|i}})-3\;\text{sinh}(\frac{\gamma_{j|i}^{\alpha_{i}}}{\delta_{j|i}})], \end{array}$$ (29)

$$\begin{array}{cc} \kappa_{j|i}^{\alpha_{i}} & =-3{\zeta \;(\gamma_{j|i}^{\alpha_{i}},\delta_{j|i})}^{4}+3{\zeta \;(\gamma_{j|i}^{\alpha_{i}},\delta_{j|i})}^{2}\;{\lambda \;(\gamma_{j|i}^{\alpha_{i}},\delta_{j|i})}^{2}[e^{\frac{2}{{\left(\delta_{j|i}\right)}^{2}}}\;\text{cosh}(2\frac{\gamma_{j|i}^{\alpha_{i}}}{\delta_{j|i}})-1]\\ & \;-\zeta \;(\gamma_{j|i}^{\alpha_{i}},\delta_{j|i}){\lambda \;(\gamma_{j|i}^{\alpha_{i}},\delta_{j|i})}^{3}\;e^{\frac{1}{2{\left(\delta_{j|i}\right)}^{2}}}[e^{\frac{4}{{\left(\delta_{j|i}\right)}^{2}}}\;\text{sinh}(3\frac{\gamma_{j|i}^{\alpha_{i}}}{\delta_{j|i}})-3\text{sinh}(\frac{\gamma_{j|i}^{\alpha_{i}}}{\delta_{j|i}})]\\ & \;+\frac{1}{8}{\lambda \;(\gamma_{j|i}^{\alpha_{i}},\delta_{j|i})}^{4}[e^{\frac{8}{{\left(\delta_{j|i}\right)}^{2}}}\;\text{cosh}(4\frac{\gamma_{j|i}^{\alpha_{i}}}{\delta_{j|i}})-4e^{\frac{2}{{\left(\delta_{j|i}\right)}^{2}}}\;\text{cosh}(2\frac{\gamma_{j|i}^{\alpha_{i}}}{\delta_{j|i}})+3], \end{array}$$ (30)

respectively. As a result, we derive the CoVaR of *X*_*j*_, given $\left\{X_{i}=-{}^{CF}{VaR}_{i}^{\alpha_{i}}\;\left(\mu_{i},\sigma_{i}^{2},\xi_{i},\kappa_{i}\right)\right\}$, with the following analytic expansion:

$$\begin{array}{c} {}^{\text{CF}}{\text{CoVaR}}_{j|i}^{\alpha_{j}}\;(\mu_{j|i}^{\alpha_{i}},\sigma_{j|i}^{2;\alpha_{i}},\xi_{j|i}^{\alpha_{i}},\kappa_{j|i}^{\alpha_{i}})=-\mu_{j|i}^{\alpha_{i}}-\sqrt{\sigma_{j|i}^{2;\alpha_{i}}}\;{}^{\text{CF}}z^{\alpha_{j}}\;(\xi_{j|i}^{\alpha_{i}},\kappa_{j|i}^{\alpha_{i}}), \end{array}$$ (31)

with

$$\begin{array}{c} {}^{\text{CF}}z^{\alpha_{j}}\;(\xi_{j|i}^{\alpha_{i}},\kappa_{j|i}^{\alpha_{i}})=z^{\alpha_{j}}+\frac{\xi_{j|i}^{\alpha_{i}}}{6}H_{2}(z^{\alpha_{j}})+\frac{\kappa_{j|i}^{\alpha_{i}}-3}{24}H_{3}(z^{\alpha_{j}})-\frac{{\left(\xi_{j|i}^{\alpha_{i}}\right)}^{2}}{36}[H_{1}(z^{\alpha_{j}})+2H_{3}(z^{\alpha_{j}})]. \end{array}$$ (32)

We then compute the corresponding ΔCoVaR as follows: ${}^{CF}{CoVaR}_{j|i}^{\alpha_{j}}\;\left(\mu_{j|i}^{\alpha_{i}},\sigma_{j|i}^{2;\alpha_{i}},\xi_{j|i}^{\alpha_{i}},\kappa_{j|i}^{\alpha_{i}}\right)-{}^{CF}{CoVaR}_{j|i}^{\alpha_{j}}\;\left(\mu_{j|i}^{50\%},\sigma_{j|i}^{2;50\%},\xi_{j|i}^{50\%},\kappa_{j|i}^{50\%}\right)$.

#### Four-moment MCoVaR

From Eq (26), we find that ${}^{CF}z^{50\%}(\xi_{k},\kappa_{k})=-\frac{\xi_{k}}{6}$. Consequently, we can verify that *X*_*j*_, given both $\left\{X_{i}=-{}^{CF}{VaR}_{i}^{\alpha_{i}}\;\left(\mu_{i},\sigma_{i}^{2},\xi_{i},\kappa_{i}\right)\right\}$ and ${\left\{X_{k}=-{}^{CF}{VaR}_{k}^{50\%}\;\left(\mu_{k},\sigma_{k}^{2},\xi_{k},\kappa_{k}\right)\right\}}_{k\in {ℐ}_{\setminus ij}}$, is distributed according to $J^{SU}\;\left(\mu_{j|i,\setminus ij}^{\alpha_{i},50\%},\sigma_{j|i,\setminus ij}^{2;\alpha_{i},50\%},\gamma_{j|i,\setminus ij}^{\alpha_{i},50\%},\delta_{j|i,\setminus ij}\right)$. We provide its conditional mean $\mu_{j|i,\setminus ij}^{\alpha_{i},50\%}$ and conditional variance $\sigma_{j|i,\setminus ij}^{2;\alpha_{i},50\%}$ in Eqs (22) and (23), respectively, with new shape parameters

$$\begin{array}{c} \gamma_{j|i,\backslash ij}^{\alpha_{i},50\%}=\frac{\gamma_{j}-P_{j,\backslash j}\;P_{\backslash j,\backslash j}^{-1}\;{}^{\text{CF}}\;y_{\backslash j}}{\sqrt{1-P_{j,\backslash j}\;P_{\backslash j,\backslash j}^{-1}\;P_{\backslash j,j}}},\;\delta_{j|i,\backslash ij}=\frac{\delta_{j}}{\sqrt{1-P_{j,\backslash j}\;P_{\backslash j,\backslash j}^{-1}\;P_{\backslash j,j}}}, \end{array}$$ (33)

where the entries of the vector ^CF^**y**_∖*j*_ are ${}^{CF}y_{k}=\gamma_{k}+\delta_{k}\;{\text{sinh}}^{-1}\;\left[\frac{{}^{CF}z^{\alpha_{k}}(\xi_{k},\kappa_{k})-\zeta (\gamma_{k},\delta_{k})}{\lambda (\gamma_{k},\delta_{k})}\right]$ for *k* = *i* and ${}^{CF}y_{k}=\gamma_{k}+\delta_{k}\;{\text{sinh}}^{-1}\;\left[\frac{-\frac{\xi_{k}}{6}-\zeta (\gamma_{k},\delta_{k})}{\lambda (\gamma_{k},\delta_{k})}\right]$ for $k\in {ℐ}_{\setminus ij}$. Meanwhile, we formulate its conditional skewness $\xi_{j|i,\setminus ij}^{\alpha_{i},50\%}$ and conditional kurtosis $\kappa_{j|i,\setminus ij}^{\alpha_{i},50\%}$ as in Eqs (29) and (30), respectively, by replacing the shape parameters $\gamma_{j|i}^{\alpha_{i},50\%}$ and *δ*_*j*|*i*_ with $\gamma_{j|i,\setminus ij}^{\alpha_{i},50\%}$ and *δ*_*j*|*i*, ∖*ij*_, respectively. Therefore, we expand the MCoVaR as follows:

$$\begin{array}{cc} & {}^{\text{CF}}{\text{MCoVaR}}_{j|i,\backslash ij}^{\alpha_{j}}\;(\mu_{j|i,\backslash ij}^{\alpha_{i},50\%},\sigma_{j|i,\backslash ij}^{2;\alpha_{i},50\%},\xi_{j|i,\backslash ij}^{\alpha_{i},50\%},\kappa_{j|i,\backslash ij}^{\alpha_{i},50\%})\\ & =-\mu_{j|i,\backslash ij}^{\alpha_{i},50\%}-\sqrt{\sigma_{j|i,\backslash ij}^{2;\alpha_{i},50\%}}\;{}^{\text{CF}}z^{\alpha_{j}}\;(\xi_{j|i,\backslash ij}^{\alpha_{i},50\%},\kappa_{j|i,\backslash ij}^{\alpha_{i},50\%}), \end{array}$$ (34)

where ${}^{CF}z^{\alpha_{j}}\;\left(\xi_{j|i,\setminus ij}^{\alpha_{i},50\%},\kappa_{j|i,\setminus ij}^{\alpha_{i},50\%}\right)$ is the Cornish–Fisher expansion involving the conditional skewness $\xi_{j|i,\setminus ij}^{\alpha_{i},50\%}$ and conditional kurtosis $\kappa_{j|i,\setminus ij}^{\alpha_{i},50\%}$. Furthermore, we obtain the corresponding ΔMCoVaR as follows: ${}^{CF}{MCoVaR}_{j|i,\setminus ij}^{\alpha_{j}}\;\left(\mu_{j|i,\setminus ij}^{\alpha_{i},50\%},\sigma_{j|i,\setminus ij}^{2;\alpha_{i},50\%},\xi_{j|i,\setminus ij}^{\alpha_{i},50\%},\kappa_{j|i,\setminus ij}^{\alpha_{i},50\%}\right)-{}^{CF}{MCoVaR}_{j|i,\setminus ij}^{\alpha_{j}}\;\left(\mu_{j|i,\setminus ij}^{50\%,50\%},\sigma_{j|i,\setminus ij}^{2;50\%,50\%},\xi_{j|i,\setminus ij}^{50\%,50\%},\kappa_{j|i,\setminus ij}^{50\%,50\%}\right)$.

### (M)CoVaR forecast and its coverage property

In practice, we cannot compute the true values of ${VaR}_{i}^{\alpha_{i}}(\theta_{i})$, ${CoVaR}_{j|i}^{\alpha_{j}}\;\left(\theta_{j|i}^{\alpha_{i}}\right)$, and ${MCoVaR}_{j|i,\setminus ij}^{\alpha_{j}}\;\left(\theta_{j|i,\setminus ij}^{\alpha_{i},50\%}\right)$ as well as their modified versions since these (conditional) tail risk measures depend on unknown parameters. Accordingly, we must forecast them by replacing such parameters with their estimators we compute based on an available dataset ${\left\{{\left(X_{i;t}\right)}_{i\in ℐ}\right\}}_{t\in T}$, where T={1,2,…,T}. Specifically, we employ the method of moments by equating

$$\begin{array}{cc} \hat{E(X_{i})}={\hat{\mu }}_{i} & =\frac{1}{T}\sum_{t\in T}X_{i;t}={\bar{X}}_{i},\\ \hat{E[{\left(X_{i}-\mu_{i}\right)}^{2}]}=\hat{\sigma_{i}^{2}} & =\frac{1}{T}\sum_{t\in T}{\left(X_{i;t}-{\bar{X}}_{i}\right)}^{2},\\ \hat{E[{\left(X_{i}-\mu_{i}\right)}^{3}]}={\hat{\xi }}_{i}\;\sqrt{{\left(\hat{\sigma_{i}^{2}}\right)}^{3}} & =\frac{1}{T}\sum_{t\in T}{\left(X_{i;t}-{\bar{X}}_{i}\right)}^{3},\\ \hat{E[{\left(X_{i}-\mu_{i}\right)}^{4}]}={\hat{\kappa }}_{i}{\left(\hat{\sigma_{i}^{2}}\right)}^{2} & =\frac{1}{T}\sum_{t\in T}{\left(X_{i;t}-{\bar{X}}_{i}\right)}^{4},\\ \hat{E[\left(X_{i}-\mu_{i}\right)\left(X_{j}-\mu_{j}\right)]}={\hat{\rho }}_{ij}\;\sqrt{\hat{\sigma_{i}^{2}}}\;\sqrt{\hat{\sigma_{j}^{2}}} & =\frac{1}{T}\sum_{t\in T}(X_{i;t}-{\bar{X}}_{i})(X_{j;t}-{\bar{X}}_{j}), \end{array}$$

and then solving for the parameter estimators. If we denote the estimator for **θ**_*i*_, $\theta_{j|i}^{\alpha_{i}}$, and $\theta_{j|i,\setminus ij}^{\alpha_{i},50\%}$ by $\hat{\theta }{}_{i}$, $\hat{\theta }{}_{j|i}^{\alpha_{i}}$, and $\hat{\theta }{}_{j|i,\setminus ij}^{\alpha_{i},50\%}$, respectively, we then obtain the estimative forecast for ${VaR}_{i}^{\alpha_{i}}(\theta_{i})$, ${CoVaR}_{j|i}^{\alpha_{j}}\;\left(\theta_{j|i}^{\alpha_{i}}\right)$, and ${MCoVaR}_{j|i,\setminus ij}^{\alpha_{j}}\;\left(\theta_{j|i,\setminus ij}^{\alpha_{i},50\%}\right)$ as follows: ${VaR}_{i}^{\alpha_{i}}(\hat{\theta }{}_{i})$, ${CoVaR}_{j|i}^{\alpha_{j}}\;\left(\hat{\theta }{}_{j|i}^{\alpha_{i}}\right)$, and ${MCoVaR}_{j|i,\setminus ij}^{\alpha_{j}}\;\left(\hat{\theta }{}_{j|i,\setminus ij}^{\alpha_{i},50\%}\right)$, respectively.

Because we define the true values of the above (conditional) tail risk measures through (conditional) coverage probabilities, we now attempt to compute their forecasts’ (conditional) coverage probabilities. For the VaR forecast, we compute ${CP}_{i}^{\alpha_{i}}(\theta_{i})=P\;\left\{X_{i}\leq -{VaR}_{i}^{\alpha_{i}}(\hat{\theta }{}_{i})\right\}$. Meanwhile, we compute the CoCP of the CoVaR forecast and the MCoCP of the MCoVaR forecast as follows:

$$\begin{array}{c} {\text{CoCP}}_{j|i}^{\alpha_{j}}\;(\theta_{j|i}^{\alpha_{i}})=P\;[\{X_{j}\leq -{\text{CoVaR}}_{j|i}^{\alpha_{j}}\;(\hat{\theta }{}_{j|i}^{\alpha_{i}})\}|\;\{X_{i}=-{\text{VaR}}_{i}^{\alpha_{i}}\left(\theta_{i}\right)\}\;] \end{array}$$

and

$$\begin{array}{cc} & {\text{MCoCP}}_{j|i,\backslash ij}^{\alpha_{j}}\;(\theta_{j|i,\backslash ij}^{\alpha_{i},50\%})\\ & =P\;[\{X_{j}\leq -{\text{MCoVaR}}_{j|i,\backslash ij}^{\alpha_{j}}\;(\hat{\theta }{}_{j|i,\backslash ij}^{\alpha_{i},50\%})\}|\;\{X_{i}=-{\text{VaR}}_{i}^{\alpha_{i}}\left(\theta_{i}\right)\}⋂{\left\{X_{k}=-{\text{VaR}}_{k}^{50\%}\left(\theta_{k}\right)\right\}}_{k\in I_{\backslash ij}}\;]. \end{array}$$

respectively. Due to the substitution of parameter estimators for the (conditional) tail risk measures, the variability of such estimators leads to the variability of their forecasts. This evidence makes the (conditional) coverage probability of each forecasted value differ from the significance level under consideration. Therefore, we assess their forecast accuracy by computing the following root-mean-square errors (RMSEs):

$$\begin{array}{cc} \text{RMSE}\;[{\text{CP}}_{i}^{\alpha_{i}}(\hat{\theta }{}_{i})] & =\sqrt{\frac{1}{L}\sum_{\ell \in L}{\left[{\text{CP}}_{i}^{\alpha_{i}}(\hat{\theta }{}_{i;\ell })-\alpha_{i}\right]}^{2}}, \end{array}$$ (35)

$$\begin{array}{cc} \text{RMSE}\;[{\text{CoCP}}_{j|i}^{\alpha_{j}}\;(\hat{\theta }{}_{j|i}^{\alpha_{i}})] & =\sqrt{\frac{1}{L}\sum_{\ell \in L}{\left[{\text{CoCP}}_{j|i}^{\alpha_{j}}\;(\hat{\theta }{}_{j|i;\ell }^{\alpha_{i}})-\alpha_{j}\right]}^{2}}, \end{array}$$ (36)

$$\begin{array}{cc} \text{RMSE}\;[{\text{MCoCP}}_{j|i,\backslash ij}^{\alpha_{j}}\;(\hat{\theta }{}_{j|i,\backslash ij}^{\alpha_{i},50\%})] & =\sqrt{\frac{1}{L}\sum_{\ell \in L}{\left[{\text{MCoCP}}_{j|i,\backslash ij}^{\alpha_{j}}\;(\hat{\theta }{}_{j|i,\backslash ij;\ell }^{\alpha_{i},50\%})-\alpha_{j}\right]}^{2}}, \end{array}$$ (37)

where ℒ={1,2,…,L}. For each ℓ∈ℒ, the terms ${CP}_{i}^{\alpha_{i}}(\hat{\theta }{}_{i;\ell })$, ${CoCP}_{j|i}^{\alpha_{j}}\;\left(\hat{\theta }{}_{j|i;\ell }^{\alpha_{i}}\right)$, and ${MCoCP}_{j|i,\setminus ij}^{\alpha_{j}}\;\left(\hat{\theta }{}_{j|i,\setminus ij;\ell }^{\alpha_{i},50\%}\right)$ denote the simulated CP of the VaR forecast, simulated CoCP of the CoVaR forecast, and simulated MCoCP of the MCoVaR forecast, respectively.

### Network construction and network topology measurement

We represent a financial system consisting of *I* entities by a directed network N=(V(N),E(N)) of order |V(N)|=I, where V(N)=ℐ denotes a set of nodes with labels 1, 2, …, *I* representing entities 1, 2, …, *I*, respectively, and E(N)⊆V(N)×V(N) symbolizes a set of directed edges. We express an edge directed from node *i* to node *j* by an ordered pair (*i*, *j*). We do not allow any loop (*i*, *i*) beginning and ending at the same node *i*. The nonexistence of any loop means that any entity cannot propagate its risk to itself directly.

Adrian and Brunnermeier [35] argued that ΔCoVaR can identify the existence of each edge. Meanwhile, Torri et al. [40] took ΔMCoVaR into consideration as an alternative to ΔCoVaR. Specifically, an edge (*i*, *j*) directed from node *i* to node *j* exists if $\Delta {CoVaR}_{j|i}^{\alpha_{j}}$ is positive (for the ΔCoVaR-based network) or if $\Delta {MCoVaR}_{j|i,\setminus ij}^{\alpha_{j}}$ is positive (for the ΔMCoVaR-based network). Meanwhile, the nonpositivity of these systemic risk measures implies the nonexistence of such a directed edge. We can thus write the edge set as follows:

$$\begin{array}{c} E\left(N\right)=\{\left(i,j\right):i,j\in V\left(N\right),i\neq j,\Delta {\text{CoVaR}}_{j|i}^{\alpha_{j}}>0\} \end{array}$$ (38)

(for the ΔCoVaR-based network) or

$$\begin{array}{c} E\left(N\right)=\{\left(i,j\right):i,j\in V\left(N\right),i\neq j,\Delta {\text{MCoVaR}}_{j|i,\backslash ij}^{\alpha_{j}}>0\} \end{array}$$ (39)

(for the ΔMCoVaR-based network).

We can uniquely determine the above network N using an adjacency matrix $A=A(N)\in {\left\{0,1\right\}}^{I\times I}$. We define its entry in position (*i*, *j*) as ${\left(A\right)}_{ij}={ℐ}_{E(N)}[(i,j)]$, where ${ℐ}_{E(N)}$ is the indicator function of E(N), i.e., (**A**)_*ij*_ = 1 if (i,j)∈E(N) and zero otherwise. Since N is directed, the adjacency matrix **A** is generally asymmetric. Furthermore, because of the absence of any loop in N, the diagonal entry (**A**)_*ii*_ is zero. In addition, we define an edge-weighting function w:E(N)→ℜ, where for each edge (i,j)∈E(N),

$$\begin{array}{c} w[(i,j)]=\frac{\Delta {\text{CoVaR}}_{j|i}^{\alpha_{j}}}{\text{max}\;\{\Delta {\text{CoVaR}}_{\ell |k}^{\alpha_{\ell }}:\left(k,\ell \right)\in E\left(N\right)\}} \end{array}$$ (40)

(for the ΔCoVaR-based network) or

$$\begin{array}{c} w[(i,j)]=\frac{\Delta {\text{MCoVaR}}_{j|i,\backslash ij}^{\alpha_{j}}}{\text{max}\;\{\Delta {\text{MCoVaR}}_{\ell |k,\backslash k\ell }^{\alpha_{\ell }}:\left(k,\ell \right)\in E\left(N\right)\}} \end{array}$$ (41)

(for the ΔMCoVaR-based network). The normalization of the edge weight implies that w[E(N)]⊆[0,1]. According to the above weighting function, we then construct a weighting matrix $W=W(N)\in {\left[0,1\right]}^{I\times I}$. We define its entries as follows: (**W**)_*ij*_ = *w*[(*i*, *j*)] when (i,j)∈E(N) and zero otherwise.

We further attempt to measure the topological structure of the resulting directed and weighted network N. First, we define the node centrality measures, which quantify the importance of each node in such N. Since the network is directed, we must break them down into out- and in-centrality measures. For each node *i*, one can define an out-degree $d_{i}^{out}=\sum_{j\in {ℐ}_{\setminus i}}{\left(A\right)}_{ij}$, denoting the number of directed edges beginning at node *i*, and an in-degree $d_{i}^{in}=\sum_{j\in {ℐ}_{\setminus i}}{\left(A\right)}_{ji}$, representing the number of directed edges ending at node *i*. One can also compute the difference between the former and the latter. We call it the net degree and symbolize it as follows: $d_{i}^{net}=d_{i}^{out}-d_{i}^{in}$. However, these degree measures supply little information about the topological structure of the network because they disregard the magnitude of the edge weights that reflect the systemic risk contributions of the linked entities. Thus, we prefer to make use of the following out- and in-strength measures [59]:

$$\begin{array}{cc} s_{i}^{\text{out}} & =\sum_{j\in I_{\backslash i}}{\left(W\right)}_{ij}, \end{array}$$ (42)

$$\begin{array}{cc} s_{i}^{\text{in}} & =\sum_{j\in I_{\backslash i}}{\left(W\right)}_{ji}, \end{array}$$ (43)

respectively. The former sums up the contributions coming from entity *i* to all the remaining entities in the risk transmission mechanism, while the latter summarizes the contributions that entity *i* receives from all the others. This suggests that the out-strength can identify a systemically important entity transmitting its risk to the remaining entities. Meanwhile, the in-strength can determine an entity becoming the most important receiver in the risk transmission mechanism. Following Diebold and Yılmaz [29] and Wen and Wang [56], we also consider the following net-strength measure:

$$\begin{array}{c} s_{i}^{\text{net}}=s_{i}^{\text{out}}-s_{i}^{\text{in}}. \end{array}$$ (44)

Its positive (negative) value indicates that entity *i* plays a role as a net transmitter (net receiver) of systemic risk.

**Fig 1:**
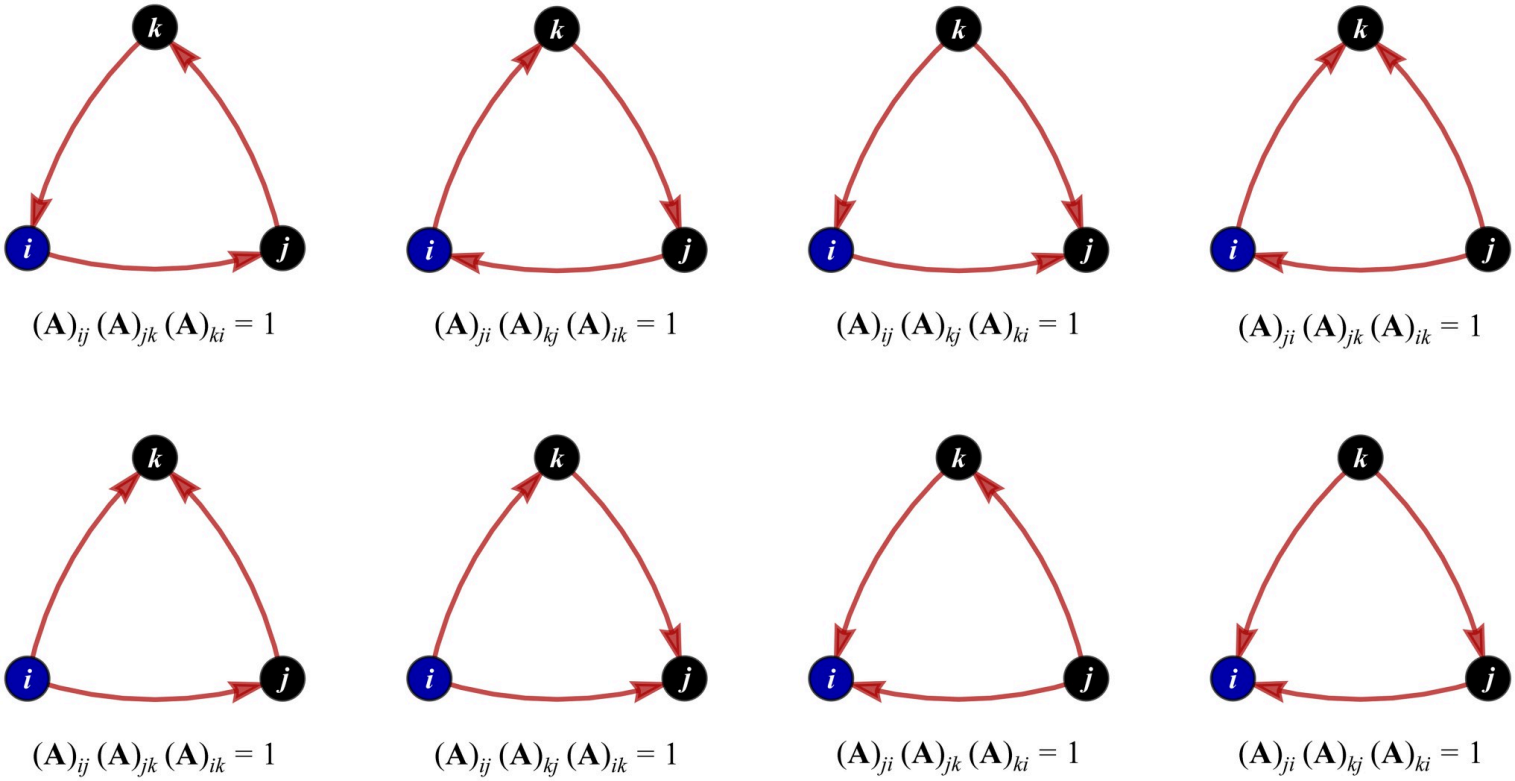
Eight different triangles containing node *i* in a directed network. The product of the form (**A**)_⋅⋅_(**A**)_⋅⋅_(**A**)_⋅⋅_ = 1 below each triangle indicates its existence in the network.

In addition, we need to measure the tendency of the neighboring nodes to cluster together using the so-called clustering coefficient. In an undirected network, Watts and Strogatz [60] defined the coefficient *c*_*i*_ of clustering around a particular node *i* as the ratio between the number of actual triangles containing node *i* and the number of all possible triangles node *i* can form. This means that *c*_*i*_ belongs to [0, 1]. Since our network N is directed, we must take account of all eight different triangles containing node *i* in such N, as we display in Fig 1. By involving the weighting matrix **W**, we follow Fagiolo [61] to formulate the extended coefficient of clustering around node *i* as follows:

$$\begin{array}{cc} c_{i} & =\frac{\frac{1}{2}\sum_{j\in I_{\backslash i}}\sum_{k\in I_{\backslash ij}}[\sqrt[{3}]{{\left(W\right)}_{ij}}+\sqrt[{3}]{{\left(W\right)}_{ji}}]\;[\sqrt[{3}]{{\left(W\right)}_{jk}}+\sqrt[{3}]{{\left(W\right)}_{kj}}]\;[\sqrt[{3}]{{\left(W\right)}_{ki}}+\sqrt[{3}]{{\left(W\right)}_{ik}}]}{d_{i}^{\text{tot}}\;(d_{i}^{\text{tot}}-1)-2d_{i}^{↔}}\\ & =\frac{\frac{1}{2}{\left({\left(\tilde{W}+{\tilde{W}}^{⊤}\right)}^{3}\right)}_{ii}}{d_{i}^{\text{tot}}\;(d_{i}^{\text{tot}}-1)-2d_{i}^{↔}} \end{array}$$ (45)

if $d_{i}^{tot}\;\left(d_{i}^{tot}-1\right)-2d_{i}^{↔}\neq 0$ and zero otherwise. Note that $\tilde{W}={\left(3\sqrt{{\left(W\right)}_{ij}}\right)}_{i,j\in I}$ is a matrix we derive from **W** by taking the third root of each entry, $d_{i}^{tot}=d_{i}^{out}+d_{i}^{in}=\sum_{j\in {ℐ}_{\setminus i}}[{\left(A\right)}_{ij}+{\left(A\right)}_{ji}]$ symbolizes the total degree of node *i*, and $d_{i}^{↔}=\sum_{j\in {ℐ}_{\setminus i}}{\left(A\right)}_{ij}\;{\left(A\right)}_{ji}={\left(A^{2}\right)}_{ii}$ denotes the number of bilateral edges between node *i* and its neighbors. We can utilize the above clustering coefficient to identify an entity that tends to make clusters together with its neighbors and propagate systemic risk.

## Results and discussion

### Data description and preliminary analysis

We applied our proposed method to quantitatively manage systemic risk in the global forex market, as a representation of the global financial system. As we mentioned in the introduction, we selected the five largest forex markets. They include the forex markets of the Eurozone, Japan, the United Kingdom, Australia, and Canada, which are advanced markets. In addition, we also chose the forex markets of five major emerging countries belonging to BRICS (Brazil, Russia, India, China, and South Africa). Therefore, we worked with ten markets; see Table 1.

**Table 1:** Ten markets and their currencies.

Advanced Market	Currency	Currency Code	*i*	Emerging Market	Currency	Currency Code	*i*
Eurozone^a^	Euro	EUR	1	Brazil	Real	BRL	6
Japan	Yen	JPY	2	Russia	Ruble	RUB	7
United Kingdom	Pound sterling	GBP	3	India	Rupee	INR	8
Australia	Dollar	AUD	4	China	Yuan	CNY	9
Canada	Dollar	CAD	5	South Africa	Rand	ZAR	10

^a^The Eurozone, which we officially call the euro area, is a group consisting of 19 out of 27 member countries of the European Union that have adopted the euro as their official currency.

To conduct an empirical study, we employed a dataset {*P*_*i*;*t*_} consisting of the daily exchange rates of each market *i*’s currency, with the US dollar being the numeraire or the base currency. We collected it from Yahoo Finance for the period ranging from January 1, 2018, to December 31, 2021. We split this data period into a subperiod before COVID-19 (i.e., January 1, 2018–March 10, 2020) and a subperiod during COVID-19 (i.e., March 11, 2020–December 31, 2021). We assumed March 11, 2020, to be the starting point of the latter subperiod based on the fact that the World Health Organization (WHO) declared the COVID-19 outbreak a pandemic on this date. We then transformed the above price dataset over each subperiod into a set {*X*_*i*;*t*_} of daily returns *X*_*i*;*t*_ = 100 ln(*P*_*i*;*t*_/*P*_*i*;*t*−1_). Over the pre-COVID-19 (respectively, COVID-19) subperiod, it contained 569 (respectively, 473) observations.

**Fig 2:**
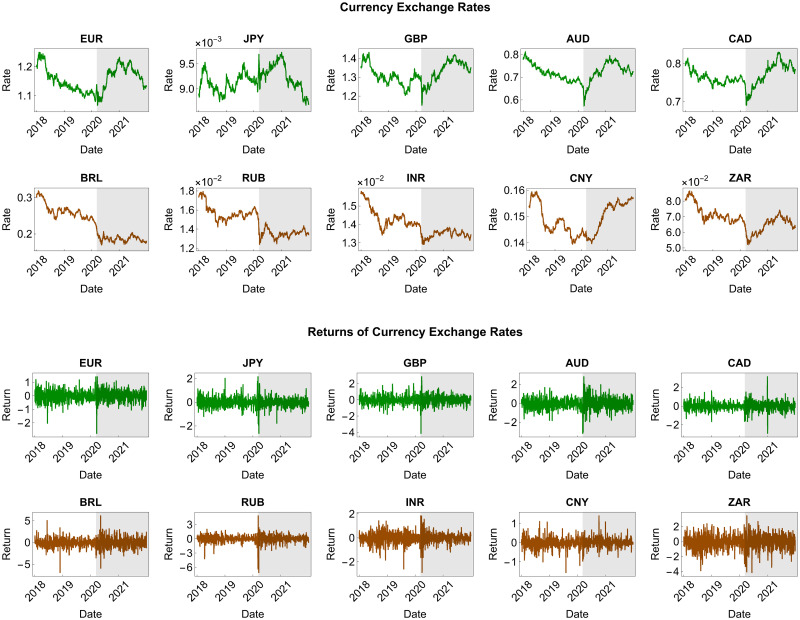
Daily exchange rates of ten currencies and their returns. We denominate each currency exchange rate in US dollars. Green and brown represent the currencies of the advanced and emerging countries, respectively. Meanwhile, the shaded region indicates the COVID-19 period ranging from March 11, 2020, to December 31, 2021.

We plot all the data in Fig 2, where the green and brown plots are for the advanced and emerging currencies, respectively. We found that the sampled exchange rates of the various currencies experienced a drastic decline at the onset of the COVID-19 pandemic. Furthermore, this pandemic resulted in more volatile exchange rates and returns, in line with the increase in the return variance we present in Table 2. An exception was the exchange rate of the Indian rupee (respectively, the Chinese yuan), whose return variance fell from 0.23 (respectively, 0.08) to 0.19 (respectively, 0.06). From Table 2, we also observe that all the returns were asymmetric and leptokurtic, with a generally negative skewness and a kurtosis above three, which were statistically significant at the 5% level according to the D’Agostino and Anscombe–Glynn tests, respectively. In addition to their variance, their kurtosis also increased dramatically, except for the Brazilian real exchange rate return, whose kurtosis declined from 9.36 to 5.17. These empirical facts strongly indicate that the normality assumption was inappropriate. We confirmed this indication using the Jarque–Bera test, significantly rejecting the null hypothesis of normality at the 5% level. Accordingly, we required Johnson’s SU distributional assumption and the asymptotic Cornish–Fisher expansion to explicitly account for the skewness and kurtosis values in modeling the returns and forecasting their tail risk measures. For the comparisons, we also considered the normal model as a benchmark.

**Table 2:** Summary statistics of the returns of currency exchange rates.

	EUR	JPY	GBP	AUD	CAD	BRL	RUB	INR	CNY	ZAR
Before COVID-19										
Mean	−0.01	0.02	−0.01	−0.03	−0.01	−0.06	−0.05	−0.03	−0.01	−0.05
Variance	0.15	0.18	0.27	0.25	0.15	0.93	0.56	0.23	0.08	0.84
Skewness	−0.10	0.77^a^	0.32^a^	−0.09	0.12	−0.34^a^	−1.86^a^	−0.43^a^	−0.40^a^	−0.29^a^
Kurtosis	4.62^b^	5.71^b^	4.44^b^	3.84^b^	5.40^b^	9.23^b^	15.58^b^	4.68^b^	6.51^b^	3.66^b^
Jarque–Bera	65.74^c^	236.64^c^	60.69^c^	18.56^c^	142.26^c^	959.78^c^	4192.72^c^	86.65^c^	317.25^c^	19.04^c^
During COVID-19										
Mean	−0.00	−0.02	0.01	0.02	0.01	−0.03	0.00	−0.00	0.02	0.00
Variance	0.18	0.17	0.33	0.49	0.26	1.61	0.77	0.19	0.06	1.07
Skewness	−0.64^a^	−0.89^a^	−0.65^a^	−0.16	−0.07	0.12	−1.04^a^	−0.43^a^	0.23^a^	−0.50^a^
Kurtosis	7.05^b^	9.26^b^	10.04^b^	5.36^b^	8.52^b^	4.98^b^	14.38^b^	9.67^b^	7.63^b^	4.21^b^
Jarque–Bera	367.78^c^	865.33^c^	1045.27^c^	116.25^c^	623.92^c^	82.31^c^	2724.54^c^	924.01^c^	443.05^c^	50.37^c^

^a^The skewness is significantly nonzero based on the D’Agostino test at the 5% level.

^b^The kurtosis is significantly above three based on the Anscombe–Glynn test at the 5% level.

^c^The Jarque–Bera test significantly rejects the null hypothesis of normality at the 5% level.

**Fig 3:**
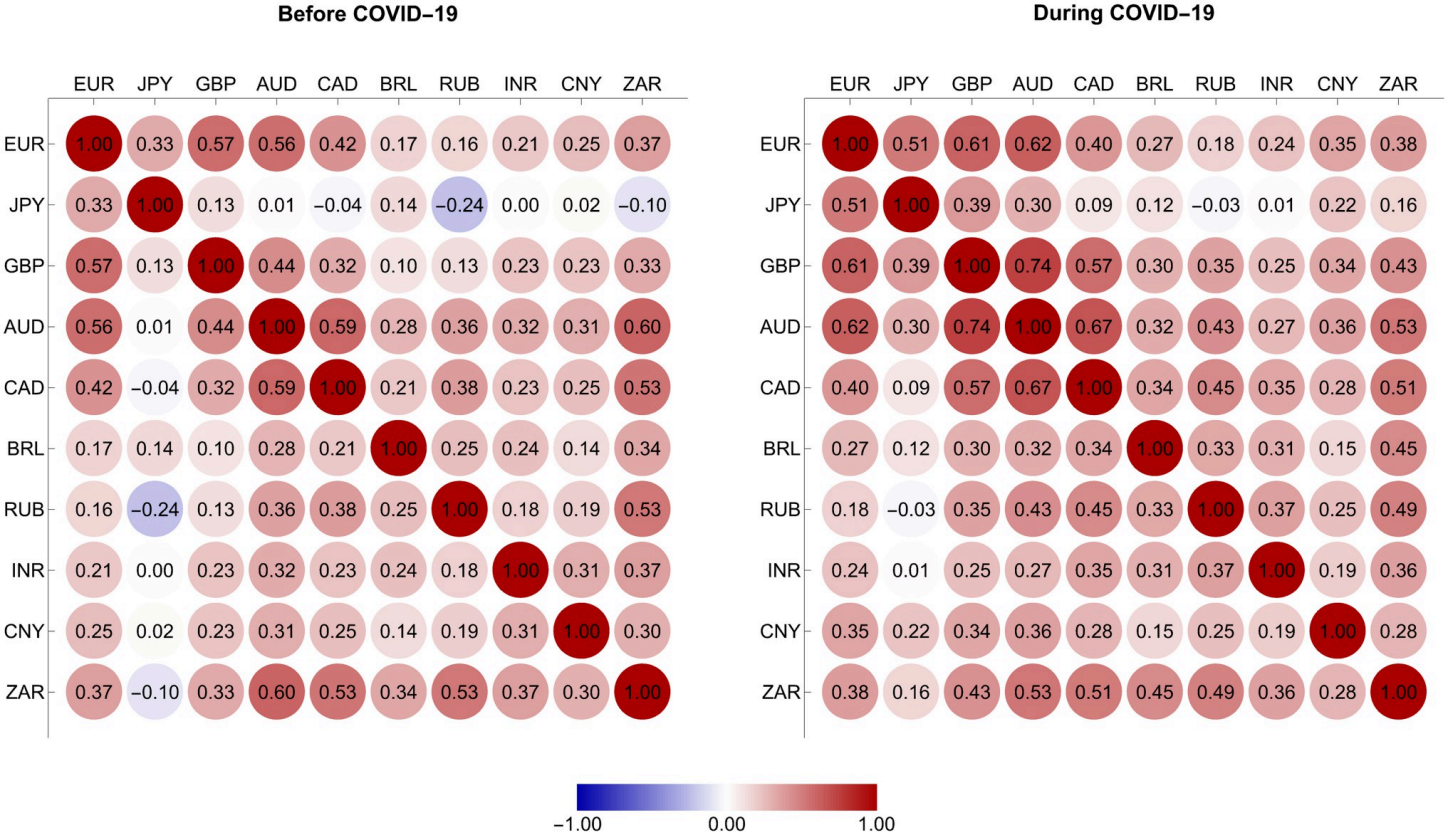
Empirical correlation matrices of the returns of currency exchange rates.

In addition, we could not ignore the dependence assumption since all the pairs of returns possessed correlation coefficients that appeared to be nonzero, as we depict in Fig 3. Their values were generally positive, meaning that the sampled currencies tended to comove in a similar direction. A notable exception was the forex rate return of the Japanese yen, which was negatively correlated with that of some other currencies. This evidence is consistent with its safe-haven property, as Ranaldo and S derlind [62] documented. We further observe from Fig 3 that the correlation of each return pair tended to be stronger as the COVID-19 crisis progressed, suggesting that the ten currencies became more interdependent during this crisis period. This result is in line with that of Wang et al. [19], demonstrating that past crises, such as the US subprime mortgage crisis, the 2008 global financial crisis, and the European sovereign debt crisis, led to an increase in the correlation coefficients between the global forex rate returns.

### Forecasted (M)CoVaR and its estimated coverage property

In Table 3, we report the root-mean-square error (RMSE) of the estimated coverage probability (CP) of each VaR we forecasted at the 5% level of significance (or at the 95% level of confidence) by first assuming that each forex market was isolated. We computed the RMSE value according to Eq (35) and utilized it to assess the accuracy of the VaR forecast. We say that a VaR forecast whose estimated CP has the lowest RMSE exhibits the best coverage performance. We found that the normal model performed the worst in forecasting the VaR for the majority of forex markets we examined before COVID-19, except for the Japanese, British, and Canadian forex markets. Amid the COVID-19 outbreak, no VaR forecast we derived using the normal model had the best coverage performance. This result is in line with evidence we reveal from Table 2 that the returns over such a period deviated further from the normality assumption due to their drastically increased kurtosis. Meanwhile, Johnson’s SU model resulted in a VaR forecast having an estimated CP with the lowest RMSE for several forex rate returns over the pre-COVID-19 and COVID-19 periods. When we expanded this model through the Cornish–Fisher expansion, the resulting VaR forecast exhibited a better coverage performance before and during COVID-19. This result demonstrates the overperformance of such an expansion method in accurately forecasting VaR.

**Table 3:** RMSE of the estimated coverage probability ${CP}_{i}^{5\%}$ of the forecasted ${VaR}_{i}^{5\%}$.

	*i*									
	EUR	JPY	GBP	AUD	CAD	BRL	RUB	INR	CNY	ZAR
Before COVID-19										
N	0.83%	**0.75%**	**0.73%**	0.77%	**0.86%**	1.15%	1.64%	0.89%	1.00%	0.80%
SU	0.84%	0.81%	0.80%	0.77%	0.89%	**1.03%**	**1.14%**	0.80%	0.89%	0.77%
CF	**0.72%**	1.47%	0.86%	**0.74%**	0.76%	1.19%	2.75%	**0.73%**	**0.34%**	**0.76%**
During COVID-19										
N	1.17%	1.38%	1.33%	0.99%	1.19%	0.90%	1.61%	1.31%	1.10%	0.97%
SU	0.96%	1.13%	**1.08%**	0.96%	**1.04%**	0.93%	**1.21%**	**1.14%**	**0.98%**	0.88%
CF	**0.54%**	**0.93%**	1.26%	**0.63%**	1.31%	**0.87%**	3.78%	1.33%	1.64%	**0.81%**

We computed each RMSE value based on Eq (35) with *L* = 10, 000 runs. For each forex market *i* over each period, we provide the lowest RMSE in boldface.

We then carried out a computation and an accuracy assessment of the CoVaR forecasts at the 5% significance level for all the possible pairs of different forex markets. We tabulate the resulting RMSEs of their estimated conditional coverage probabilities (CoCPs) in Tables 4 and 5 and summarize them in Table 6. The targeted forex market *j* in each column of the former two tables acts as a risk receiver, while the conditioning forex market *i* in each row serves as a risk emitter. Surprisingly, we found the normal model and the Cornish–Fisher expansion to perform competitively in producing CoVaR forecasts with the best conditional coverage property prior to COVID-19. Nevertheless, the latter was superior to the former in forecasting an accurate CoVaR for most paired forex markets over the COVID-19 period. Johnson’s SU model, on the other hand, offered the most accurate CoVaR forecast only for the Russian forex market we regarded as the targeted forex market. A large number of parameters in its bivariate form may cause its worst performance in the majority of cases. The large variability of its estimated parameters results in a high variability of the estimated CoCP of the resulting CoVaR forecast, as we indicate from its high RMSE. However, we could overcome this weakness of Johnson’s SU model using the Cornish–Fisher expansion, which substantially reduced the RMSE.

**Table 4:** RMSE of the estimated conditional coverage probability ${CoCP}_{j|i}^{5\%}$ of the forecasted ${CoVaR}_{j|i}^{5\%}$ before COVID-19.

			***j***									
			**EUR**	**JPY**	**GBP**	**AUD**	**CAD**	**BRL**	**RUB**	**INR**	**CNY**	**ZAR**
*i*	EUR	N	-	**1.26%**	**1.20%**	**1.29%**	1.25%	**1.43%**	1.26%	1.05%	1.22%	**1.22%**
		SU	-	1.42%	1.52%	1.54%	1.38%	1.61%	**0.96%**	1.06%	1.02%	1.34%
		CF	-	1.34%	1.48%	1.53%	**1.08%**	1.47%	2.66%	**0.95%**	**0.82%**	1.33%
	JPY	N	**1.23%**	-	1.01%	1.17%	1.28%	1.52%	1.32%	1.12%	1.18%	1.17%
		SU	1.53%	-	1.05%	1.10%	1.16%	1.57%	1.51%	1.00%	1.03%	1.03%
		CF	1.34%	-	**0.99%**	**1.01%**	**0.96%**	**1.34%**	**1.25%**	**0.84%**	**0.41%**	**0.95%**
	GBP	N	**1.39%**	**1.16%**	-	**1.24%**	1.18%	1.40%	1.47%	1.19%	1.37%	**1.15%**
		SU	2.28%	1.38%	-	1.35%	1.24%	1.43%	**1.12%**	1.28%	1.29%	1.18%
		CF	2.06%	1.76%	-	1.30%	**0.94%**	**1.25%**	2.98%	**1.12%**	**0.81%**	1.16%
	AUD	N	**1.41%**	**1.05%**	**1.17%**	-	**1.44%**	**1.48%**	1.95%	**1.22%**	1.38%	**1.33%**
		SU	2.36%	1.44%	1.36%	-	1.78%	1.87%	**1.75%**	1.44%	1.30%	1.62%
		CF	2.13%	2.28%	1.28%	-	1.46%	1.64%	3.21%	1.26%	**0.84%**	1.61%
	CAD	N	**1.34%**	**1.14%**	1.09%	**1.32%**	-	**1.50%**	2.43%	1.16%	1.36%	**1.33%**
		SU	1.99%	1.57%	1.15%	1.53%	-	1.72%	**2.27%**	1.28%	1.24%	1.60%
		CF	1.76%	2.67%	**1.06%**	1.53%	-	1.55%	3.39%	**1.13%**	**0.82%**	1.62%
	BRL	N	**1.27%**	**1.19%**	0.99%	**1.12%**	1.15%	-	2.00%	1.29%	1.41%	**1.20%**
		SU	1.49%	1.21%	0.98%	1.13%	1.28%	-	**1.70%**	1.46%	1.33%	1.31%
		CF	1.29%	1.43%	**0.95%**	1.12%	**0.92%**	-	3.76%	**1.29%**	**0.73%**	1.36%
	RUB	N	**1.46%**	**1.58%**	**1.19%**	**1.17%**	**1.49%**	**1.61%**	-	1.14%	1.38%	**1.31%**
		SU	1.79%	2.81%	1.25%	1.19%	1.92%	1.98%	-	1.25%	1.28%	1.68%
		CF	1.58%	5.21%	1.20%	1.18%	1.50%	1.76%	-	**1.13%**	**0.81%**	1.80%
	INR	N	**1.27%**	**1.08%**	1.07%	1.09%	1.17%	**1.46%**	1.91%	-	1.66%	**1.20%**
		SU	1.56%	1.30%	1.14%	1.12%	1.26%	1.77%	**1.55%**	-	1.75%	1.32%
		CF	1.34%	2.03%	**1.05%**	**1.08%**	**0.89%**	1.65%	3.78%	-	**0.91%**	1.31%
	CNY	N	**1.21%**	**1.27%**	1.09%	1.09%	1.13%	**1.29%**	1.74%	**1.55%**	-	**1.19%**
		SU	1.51%	1.36%	1.14%	1.10%	1.22%	1.43%	**1.41%**	1.89%	-	1.28%
		CF	1.33%	1.95%	**1.07%**	**1.07%**	**0.89%**	1.36%	3.55%	1.66%	-	1.28%
	ZAR	N	**1.28%**	**1.10%**	1.12%	**1.30%**	1.47%	**1.50%**	2.62%	**1.33%**	1.40%	-
		SU	1.86%	1.61%	1.19%	1.50%	1.84%	2.04%	2.54%	1.67%	1.37%	-
		CF	1.63%	2.94%	**1.09%**	1.46%	**1.44%**	1.64%	**1.89%**	1.44%	**0.85%**	-

Forex market *i* we present in each row acts as a risk transmitter, while forex market *j* in each column serves as a risk receiver. We computed each RMSE value based on Eq (36) with *L* = 10, 000 runs. For each pair of different forex markets *i* and *j*, we provide the lowest RMSE in boldface.

**Table 5:** RMSE of the estimated conditional coverage probability ${CoCP}_{j|i}^{5\%}$ of the forecasted ${CoVaR}_{j|i}^{5\%}$ during COVID-19.

			***j***									
			**EUR**	**JPY**	**GBP**	**AUD**	**CAD**	**BRL**	**RUB**	**INR**	**CNY**	**ZAR**
*i*	EUR	N	-	2.54%	2.29%	**1.73%**	1.47%	1.26%	**2.20%**	**1.86%**	1.76%	1.39%
		SU	-	2.58%	3.02%	2.07%	1.62%	1.51%	2.23%	1.91%	1.64%	1.38%
		CF	-	**1.00%**	**1.01%**	1.75%	**1.06%**	**1.25%**	5.79%	1.93%	**0.85%**	**1.29%**
	JPY	N	**2.19%**	-	2.15%	1.55%	1.53%	1.29%	2.50%	1.71%	1.71%	1.43%
		SU	3.69%	-	2.44%	1.68%	1.47%	1.52%	**2.45%**	1.76%	1.60%	1.38%
		CF	2.21%	-	**1.97%**	**1.22%**	**0.78%**	**1.27%**	4.88%	**1.51%**	**0.58%**	**1.26%**
	GBP	N	**2.00%**	2.25%	-	**2.00%**	1.94%	**1.34%**	**3.12%**	1.92%	1.69%	**1.54%**
		SU	3.70%	2.08%	-	2.79%	2.05%	1.65%	3.58%	2.02%	1.59%	1.65%
		CF	2.62%	**1.22%**	-	3.12%	**0.95%**	1.41%	5.89%	**1.91%**	**0.84%**	1.65%
	AUD	N	**2.01%**	2.19%	2.78%	-	2.24%	1.38%	**2.69%**	1.94%	1.76%	1.60%
		SU	3.77%	1.98%	3.97%	-	2.41%	1.66%	3.25%	2.11%	1.66%	1.68%
		CF	2.56%	**1.43%**	**2.67%**	-	**1.32%**	**1.37%**	4.65%	**1.91%**	**0.85%**	**1.60%**
	CAD	N	1.78%	1.75%	2.31%	**1.87%**	-	**1.37%**	**2.50%**	2.06%	1.57%	**1.58%**
		SU	2.59%	1.51%	3.00%	2.38%	-	1.71%	2.93%	2.10%	1.44%	1.65%
		CF	**1.19%**	**1.27%**	**1.06%**	2.44%	-	1.50%	4.08%	**1.73%**	**0.69%**	1.73%
	BRL	N	1.43%	1.63%	**1.82%**	1.37%	1.54%	-	**2.25%**	1.85%	1.37%	1.45%
		SU	1.86%	1.45%	2.04%	1.51%	1.51%	-	2.34%	1.90%	1.21%	1.44%
		CF	**0.89%**	**1.32%**	2.13%	**1.10%**	**1.10%**	-	5.33%	**1.80%**	**0.18%**	**1.33%**
	RUB	N	1.26%	1.51%	2.80%	**1.87%**	1.87%	**1.31%**	-	1.92%	1.53%	**1.58%**
		SU	1.38%	1.40%	3.28%	2.35%	1.96%	1.65%	-	2.06%	1.48%	1.75%
		CF	**0.86%**	**0.76%**	**2.12%**	1.94%	**0.98%**	1.49%	-	**1.71%**	**0.65%**	1.88%
	INR	N	1.57%	1.69%	**2.19%**	1.71%	1.71%	**1.49%**	**2.54%**	-	1.58%	1.48%
		SU	1.89%	1.49%	2.24%	1.85%	1.68%	1.79%	2.73%	-	1.46%	1.45%
		CF	**0.88%**	**0.98%**	2.25%	**1.32%**	**1.10%**	1.52%	5.31%	-	**0.42%**	**1.38%**
	CNY	N	1.94%	2.11%	2.11%	1.68%	1.55%	1.37%	**2.33%**	**1.76%**	-	1.37%
		SU	2.60%	1.95%	2.33%	1.90%	1.58%	1.47%	2.37%	1.80%	-	1.32%
		CF	**1.11%**	**1.42%**	**2.00%**	**1.42%**	**1.06%**	**1.17%**	5.78%	1.77%	-	**1.22%**
	ZAR	N	1.69%	1.73%	2.15%	1.67%	1.93%	**1.39%**	**2.67%**	2.09%	1.56%	-
		SU	2.53%	1.65%	2.73%	2.05%	1.95%	1.86%	3.14%	2.21%	1.41%	-
		CF	**1.05%**	**1.42%**	**1.83%**	**1.55%**	**0.90%**	1.57%	3.87%	**1.84%**	**0.76%**	-

Forex market *i* we present in each row acts as a risk transmitter, while forex market *j* in each column serves as a risk receiver. We computed each RMSE value based on Eq (36) with *L* = 10, 000 runs. For each pair of different forex markets *i* and *j*, we provide the lowest RMSE in boldface.

**Table 6:** Number of ${CoVaR}_{j|\cdot }^{5\%}$ models with the best conditional coverage property.

	*j*									
	EUR	JPY	GBP	AUD	CAD	BRL	RUB	INR	CNY	ZAR
Before COVID-19										
N	9	9	3	6	2	7	0	3	0	8
SU	0	0	0	0	0	0	7	0	0	0
CF	0	0	6	3	7	2	2	6	9	1
During COVID-19										
N	3	0	2	4	0	5	8	2	0	3
SU	0	0	0	0	0	0	1	0	0	0
CF	6	9	7	5	9	4	0	7	9	6

This table summarizes Tables 4 and 5 by counting the number of the best ${CoVaR}_{j|\cdot }^{\text{5\%}}$ models (i.e., the normal model, Johnson’s SU model, and Cornish–Fisher expansion) for each targeted forex market *j*, whose estimated conditional coverage probability ${CoCP}_{j|\cdot }^{5\%}$ has the lowest RMSE.

We now provide in Tables 7 and 8 the RMSEs of the estimated MCoCPs of the forecasted MCoVaRs at the 5% level of significance for all the possible pairs of targeted and distressing forex markets. Based on their summary in Table 9, we also uncovered the superiority of the Cornish–Fisher expansion in accurately forecasting MCoVaR before and during the COVID-19 period. More specifically, the expanded MCoVaR forecast possessed an estimated MCoCP with the lowest RMSE in most cases. This MCoVaR forecasting result before COVID-19 is contrary to the previous CoVaR forecasting result, where the Cornish–Fisher expansion and the normal model competed in many cases. These findings indicate this expansion’s notable ability to reduce the variability of the estimated MCoCP of the unexpanded MCoVaR forecast resulting from the multivariate Johnson’s SU model.

**Table 7:** RMSE of the estimated conditional coverage probability ${MCoCP}_{j|i,\setminus ij}^{5\%}$ of the forecasted ${MCoVaR}_{j|i,\setminus ij}^{5\%}$ before COVID-19.

			***j***									
			**EUR**	**JPY**	**GBP**	**AUD**	**CAD**	**BRL**	**RUB**	**INR**	**CNY**	**ZAR**
*i*	EUR	N	-	**1.48%**	**1.42%**	**1.88%**	1.53%	1.78%	1.74%	**1.43%**	1.58%	1.70%
		SU	-	2.08%	1.75%	1.97%	1.54%	1.53%	2.23%	1.56%	1.56%	1.62%
		CF	-	2.14%	1.71%	1.94%	**1.39%**	**1.16%**	**1.67%**	1.50%	**1.06%**	**1.60%**
	JPY	N	1.49%	-	**1.29%**	**1.41%**	1.68%	1.98%	**1.75%**	1.29%	1.54%	**1.33%**
		SU	1.43%	-	1.43%	1.55%	1.59%	2.07%	2.27%	1.23%	1.48%	1.39%
		CF	**1.40%**	-	1.41%	1.55%	**1.49%**	**0.80%**	2.17%	**1.12%**	**0.92%**	1.41%
	GBP	N	1.63%	**1.29%**	-	1.47%	1.33%	1.63%	2.16%	1.41%	1.66%	**1.31%**
		SU	1.64%	1.60%	-	1.45%	1.28%	1.23%	2.00%	1.32%	1.59%	1.31%
		CF	**1.58%**	1.80%	-	**1.42%**	**1.17%**	**0.73%**	**1.38%**	**1.19%**	**0.97%**	1.31%
	AUD	N	2.04%	**1.57%**	1.61%	-	1.66%	1.80%	1.94%	1.44%	1.59%	1.51%
		SU	1.96%	1.84%	1.66%	-	1.80%	1.64%	1.95%	1.32%	1.49%	1.52%
		CF	**1.89%**	2.13%	**1.60%**	-	**1.61%**	**0.57%**	**1.25%**	**1.20%**	**0.95%**	**1.51%**
	CAD	N	1.60%	**1.66%**	**1.26%**	**1.56%**	-	1.79%	2.95%	1.28%	1.61%	**1.95%**
		SU	1.44%	1.71%	1.36%	1.61%	-	1.41%	2.08%	1.22%	1.50%	2.03%
		CF	**1.37%**	1.85%	1.32%	1.58%	-	**0.36%**	**0.84%**	**1.11%**	**0.93%**	2.03%
	BRL	N	1.52%	**1.56%**	1.20%	1.31%	1.16%	-	2.41%	1.30%	1.46%	1.32%
		SU	1.32%	1.69%	1.26%	1.32%	1.19%	-	2.10%	1.25%	1.35%	1.32%
		CF	**1.32%**	1.70%	**1.23%**	**1.30%**	**1.07%**	-	**1.04%**	**1.13%**	**0.81%**	**1.31%**
	RUB	N	**1.45%**	**1.52%**	**1.31%**	**1.39%**	2.32%	2.03%	-	1.13%	1.44%	**1.45%**
		SU	1.74%	2.09%	1.49%	1.51%	2.51%	1.99%	-	1.15%	1.37%	1.60%
		CF	1.75%	2.41%	1.47%	1.49%	**2.29%**	**0.69%**	-	**1.07%**	**0.84%**	1.57%
	INR	N	1.38%	**1.19%**	**1.29%**	1.27%	1.41%	1.73%	2.11%	-	1.94%	**1.42%**
		SU	**1.32%**	1.43%	1.35%	1.27%	1.31%	1.55%	1.67%	-	1.97%	1.43%
		CF	1.33%	1.60%	1.31%	**1.25%**	**1.19%**	**0.55%**	**1.04%**	-	**1.18%**	1.42%
	CNY	N	1.39%	**1.52%**	**1.36%**	1.31%	1.36%	1.39%	1.88%	1.73%	-	1.32%
		SU	**1.35%**	1.62%	1.41%	1.32%	1.31%	1.15%	1.49%	1.75%	-	1.29%
		CF	1.37%	1.72%	1.37%	**1.31%**	**1.18%**	**0.32%**	**0.92%**	**1.56%**	-	**1.29%**
	ZAR	N	1.71%	**1.49%**	1.40%	1.54%	2.37%	1.63%	3.03%	1.62%	1.47%	-
		SU	**1.70%**	1.62%	1.44%	1.56%	2.35%	1.75%	2.56%	1.65%	1.45%	-
		CF	1.74%	1.83%	**1.39%**	**1.53%**	**2.13%**	**0.75%**	**1.00%**	**1.47%**	**0.92%**	-

Forex market *i* we present in each row acts as a risk transmitter, while forex market *j* in each column serves as a risk receiver. We computed each RMSE value based on Eq (37) with *L* = 10, 000 runs. For each pair of different forex markets *i* and *j*, we provide the lowest RMSE in boldface.

**Table 8:** RMSE of the estimated conditional coverage probability ${MCoCP}_{j|i,\setminus ij}^{5\%}$ of the forecasted ${MCoVaR}_{j|i,\setminus ij}^{5\%}$ during COVID-19.

			***j***									
			**EUR**	**JPY**	**GBP**	**AUD**	**CAD**	**BRL**	**RUB**	**INR**	**CNY**	**ZAR**
*i*	EUR	N	-	2.66%	**1.82%**	**2.39%**	2.39%	1.63%	**2.29%**	2.56%	2.18%	2.00%
		SU	-	2.91%	2.14%	2.40%	2.78%	1.71%	4.07%	2.41%	2.17%	2.04%
		CF	-	**1.40%**	3.26%	2.40%	**2.37%**	**1.53%**	7.73%	**0.86%**	**1.08%**	**1.99%**
	JPY	N	2.56%	-	2.57%	**2.14%**	2.93%	1.77%	2.16%	1.69%	1.63%	1.54%
		SU	2.05%	-	**1.79%**	2.40%	2.36%	1.90%	**2.05%**	1.74%	1.44%	1.52%
		CF	**1.61%**	-	1.93%	2.53%	**2.28%**	**1.71%**	2.22%	**1.23%**	**0.66%**	**1.47%**
	GBP	N	**2.19%**	2.59%	-	**3.04%**	3.05%	1.82%	4.60%	2.17%	1.72%	**2.02%**
		SU	2.27%	2.76%	-	3.20%	2.19%	1.85%	3.36%	2.19%	1.78%	2.20%
		CF	2.62%	**1.60%**	-	3.09%	**1.80%**	**1.66%**	**1.34%**	**0.97%**	**1.01%**	2.16%
	AUD	N	2.76%	2.90%	3.32%	-	3.15%	2.26%	2.42%	2.77%	3.09%	2.03%
		SU	2.73%	3.11%	2.93%	-	2.83%	2.22%	2.26%	2.82%	3.24%	1.94%
		CF	**2.67%**	**2.70%**	**2.33%**	-	**2.20%**	**2.11%**	**1.19%**	**2.41%**	**2.04%**	**1.87%**
	CAD	N	**2.79%**	**2.50%**	**1.89%**	2.00%	-	1.57%	**1.65%**	2.62%	1.67%	2.04%
		SU	2.81%	2.66%	2.01%	2.02%	-	1.67%	1.75%	2.49%	1.63%	2.03%
		CF	3.27%	2.52%	2.89%	**1.97%**	-	**1.48%**	1.73%	**0.93%**	**1.03%**	**1.95%**
	BRL	N	**1.69%**	1.83%	**1.60%**	**2.02%**	2.14%	-	2.04%	1.89%	1.45%	1.69%
		SU	1.79%	1.82%	1.79%	2.19%	1.52%	-	**1.55%**	1.85%	1.34%	1.64%
		CF	2.38%	**1.29%**	3.28%	2.31%	**1.14%**	-	1.74%	**0.69%**	**1.18%**	**1.57%**
	RUB	N	**2.01%**	**1.73%**	3.60%	**2.18%**	2.47%	1.42%	-	1.90%	1.56%	1.74%
		SU	3.35%	2.21%	2.24%	2.21%	1.88%	1.52%	-	1.93%	1.47%	1.73%
		CF	4.64%	1.91%	**1.43%**	2.21%	**1.52%**	**1.35%**	-	**0.83%**	**0.63%**	**1.65%**
	INR	N	1.98%	1.56%	2.01%	**1.74%**	2.82%	1.70%	2.34%	-	1.65%	**1.81%**
		SU	**1.80%**	1.61%	**1.71%**	1.88%	2.23%	1.65%	1.63%	-	1.54%	1.86%
		CF	2.11%	**1.17%**	2.99%	1.98%	**1.83%**	**1.47%**	**0.98%**	-	**0.81%**	1.82%
	CNY	N	2.00%	1.63%	1.82%	**2.45%**	2.23%	1.49%	1.91%	1.84%	-	1.59%
		SU	**1.49%**	1.45%	**1.64%**	2.46%	1.64%	1.39%	1.22%	1.73%	-	1.54%
		CF	1.67%	**0.98%**	2.80%	2.46%	**1.23%**	**1.25%**	**1.08%**	**0.46%**	-	**1.49%**
	ZAR	N	1.98%	1.80%	**2.07%**	**1.94%**	3.38%	1.50%	2.51%	2.66%	1.72%	-
		SU	**1.72%**	1.83%	2.31%	1.97%	2.50%	1.60%	1.81%	2.49%	1.66%	-
		CF	2.14%	**1.23%**	3.91%	1.98%	**2.04%**	**1.44%**	**1.15%**	**0.88%**	**0.85%**	-

Forex market *i* we present in each row acts as a risk transmitter, while forex market *j* in each column serves as a risk receiver. We computed each RMSE value based on Eq (37) with *L* = 10, 000 runs. For each pair of different forex markets *i* and *j*, we provide the lowest RMSE in boldface.

**Table 9:** Number of ${MCoVaR}_{j|\cdot }^{5\%}$ models with the best conditional coverage property.

	*j*									
	EUR	JPY	GBP	AUD	CAD	BRL	RUB	INR	CNY	ZAR
Before COVID-19										
N	1	9	6	4	0	0	1	1	0	5
SU	3	0	0	0	0	0	0	0	0	0
CF	5	0	3	5	9	9	8	8	9	4
During COVID-19										
N	4	2	4	8	0	0	2	0	0	2
SU	3	0	3	0	0	0	2	0	0	0
CF	2	7	2	1	9	9	5	9	9	7

This table summarizes Tables 7 and 8 by counting the number of the best ${MCoVaR}_{j|\cdot }^{5\%}$ models (i.e., the normal model, Johnson’s SU model, and Cornish–Fisher expansion) for each targeted forex market *j*, whose estimated conditional coverage probability ${MCoCP}_{j|\cdot }^{5\%}$ has the lowest RMSE.

The superior performance of the Cornish–Fisher expansion method in modifying the (conditional) tail risk measures, which are basically the quantiles or upper forecasting limits of the (conditional) risk models, is consistent with the findings of previous studies. For instance, Alexander et al. [2] made use of this expansion to approximate the VaR of the aggregated returns of a given asset over a fixed time horizon when the return model obeyed a normal or Student’s *t* distribution. Their approach required less time-consuming simulations and provided a satisfactory degree of accuracy. In addition, Boudt et al. [9] considered expanding a skewed Student’s *t* return model, and the resulting expanded VaR was a good approximation of the portfolio VaR. Gueyi and Amvella [8] and You and Daigler [10], who conducted empirical studies on portfolio (risk) management, supported the above result. More specifically, the former study found the expanded VaR to exhibit a better risk-adjusted performance when using it in portfolio optimization. Meanwhile, the latter study discovered the importance and significance of involving skewness and kurtosis in individual and portfolio VaR expansions for determining the benefits of portfolio diversification. Recent studies, e.g., [57, 63, 64], also noticed the importance of taking a skewed and leptokurtic model into consideration for forecasting conditional or systemic risk measures. Nonetheless, their formulation took no analytical account of its conditional skewness and conditional kurtosis.

In addition, the overperformance of the Cornish–Fisher expansion we revealed in this study is also in line with the more accurate inference procedures some authors arranged through this expansion method. For instance, Johnson [65] introduced a modified one-sample *t*-test based on the expanded quantile function of the statistics of interest (e.g., sample mean), resulting in an improved testing procedure. Boik [66] and Boik and Haaland [67] constructed confidence intervals for 1) regression parameters and 2) functions of correlation coefficients, respectively, by expanding their confidence limits. They showed that their coverage error decreased at a rate faster than that of conventional confidence intervals they developed on the basis of the central limit theorem. In contrast, Boudt et al. [9] highlighted that the Cornish–Fisher expansion might become less reliable when the actual distribution deviated far from normality. See also [7].

### Tail risk networks and their topology measures

**Fig 4:**
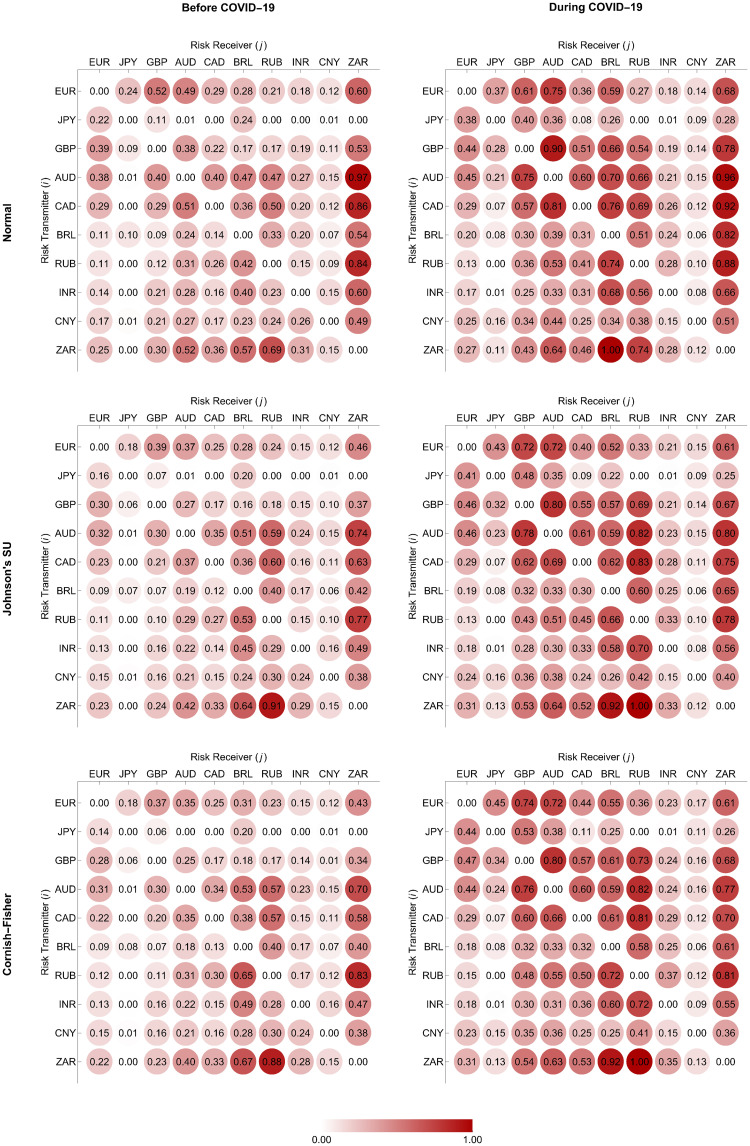
Normalized weighting matrices based on ΔCoVaR forecasts.

**Fig 5:**
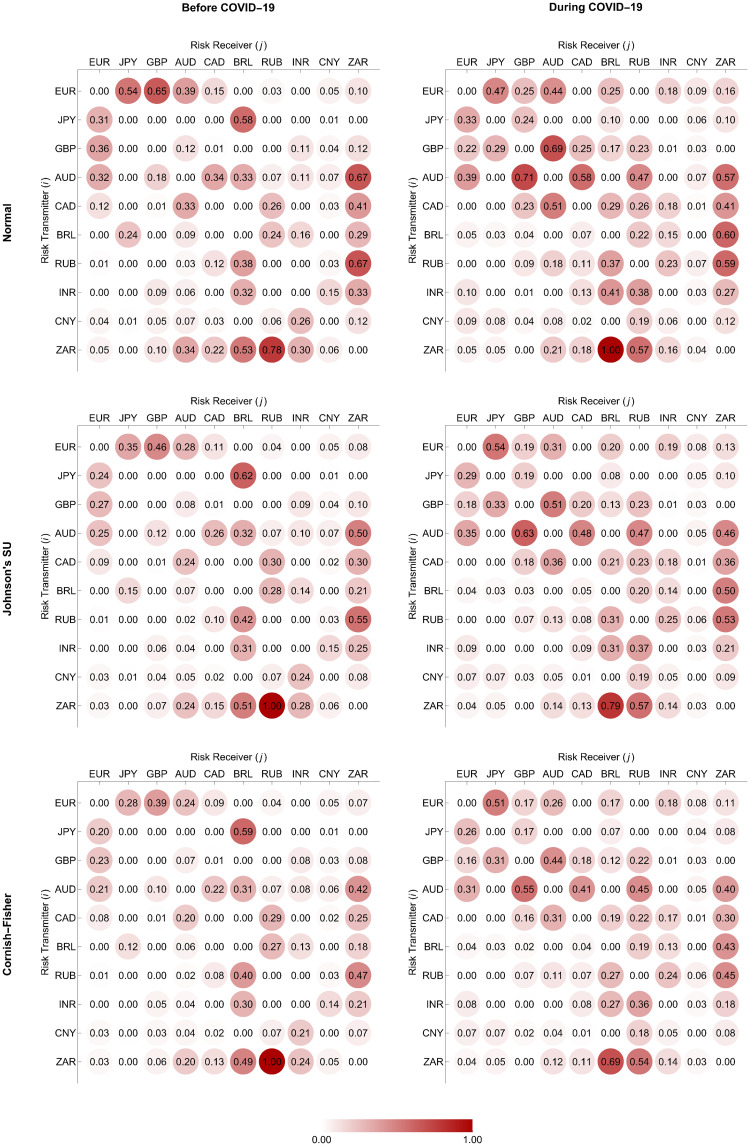
Normalized weighting matrices based on ΔMCoVaR forecasts.

**Fig 6:**
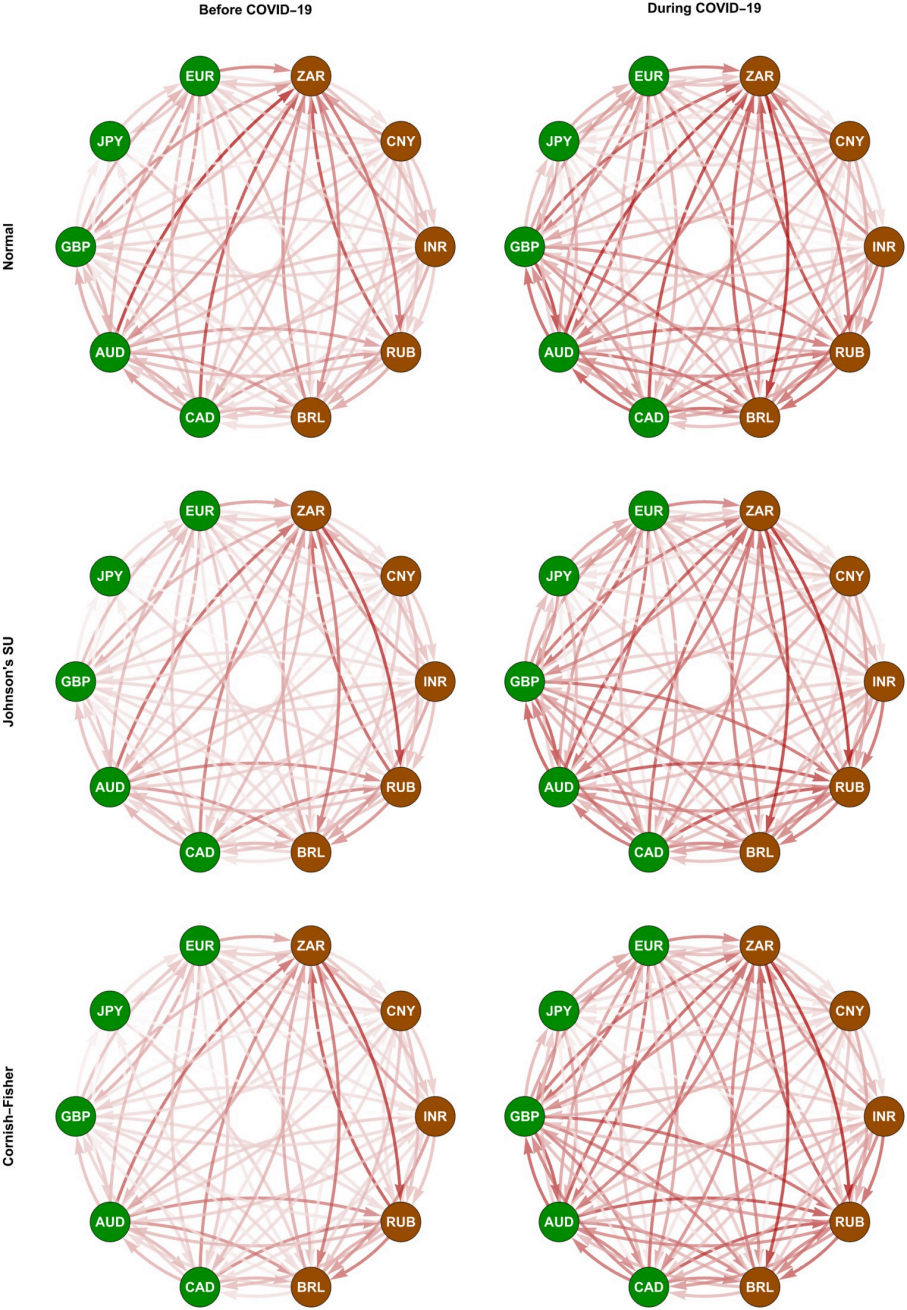
Conditional tail risk networks based on ΔCoVaR forecasts. The green and brown nodes represent the advanced and emerging forex markets, respectively. The darkness of each edge indicates the magnitude of its weight, as we present in Fig 4.

**Fig 7:**
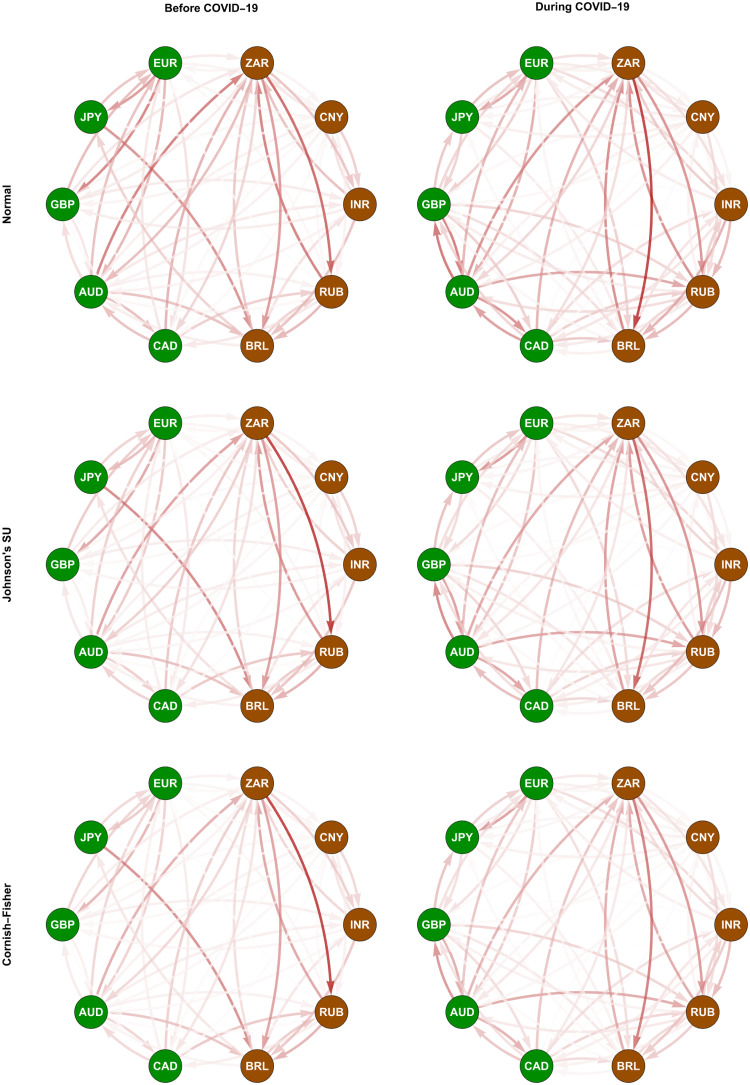
Conditional tail risk networks based on ΔMCoVaR forecasts. The green and brown nodes represent the advanced and emerging forex markets, respectively. The darkness of each edge indicates the magnitude of its weight, as we present in Fig 5.

In Figs 4 and 5, we depict the normalized weighting matrices we determine using the ΔCoVaR and ΔMCoVaR forecasts with positive values according to Eqs (40) and (41), respectively. For each pair of a transmitter and a receiver, the positivity of these systemic risk measure forecasts indicates the presence of a risk transmission channel or link if the former moves from its median state to financial distress. We represent all the possible links using directed and weighted edges, as we display in Figs 6 and 7. An arrow at the end of each edge indicates the direction of the risk transmission (from a transmitter to a receiver), and its darkness reflects the magnitude of the weight we assign. The constructed tail risk networks in these figures completely describe the global forex markets of the advanced and emerging countries we represent using the green and brown nodes, respectively. More specifically, these networks provide information about the tail risk interconnectedness reflecting the systemic risk contributions of one forex market to the others.

We observe from Figs 6 and 7 that the considered forex markets were more interconnected in terms of systemic risk transmission due to the COVID-19 outbreak, as we indicate from their edges appearing to become darker. This evidence is in line with what Wen and Wang [56] found from their study on the volatility connectedness network across global forex markets. More specifically, they revealed evidence that their volatility connectedness increased as a result of several major stressful events during the sample period of 2000–2019. These events include 1) the US recession due to the dot-com bubble burst, 2) a recession in Europe resulting from the euro depreciation, 3) the 2008 global financial crisis, 4) the European sovereign debt crisis, and 5) Brexit (Britain exiting the European Union). Greenwood-Nimmo et al. [51] and Polat [52], who focused only on advanced forex markets, pointed out similar evidence.

**Fig 8:**
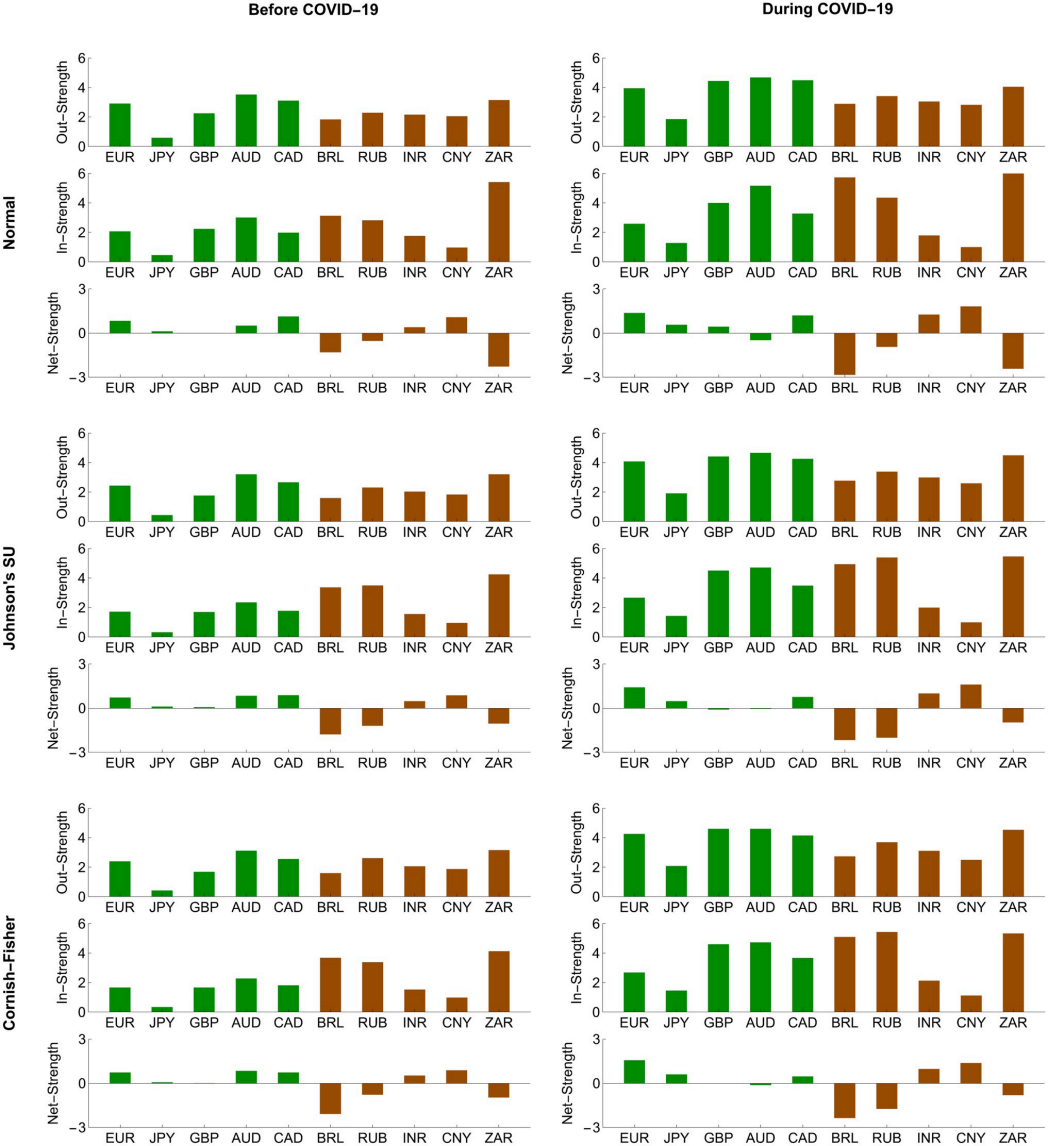
Node strength measures of conditional tail risk networks based on ΔCoVaR forecasts. Green and brown represent the advanced and emerging forex markets, respectively.

**Fig 9:**
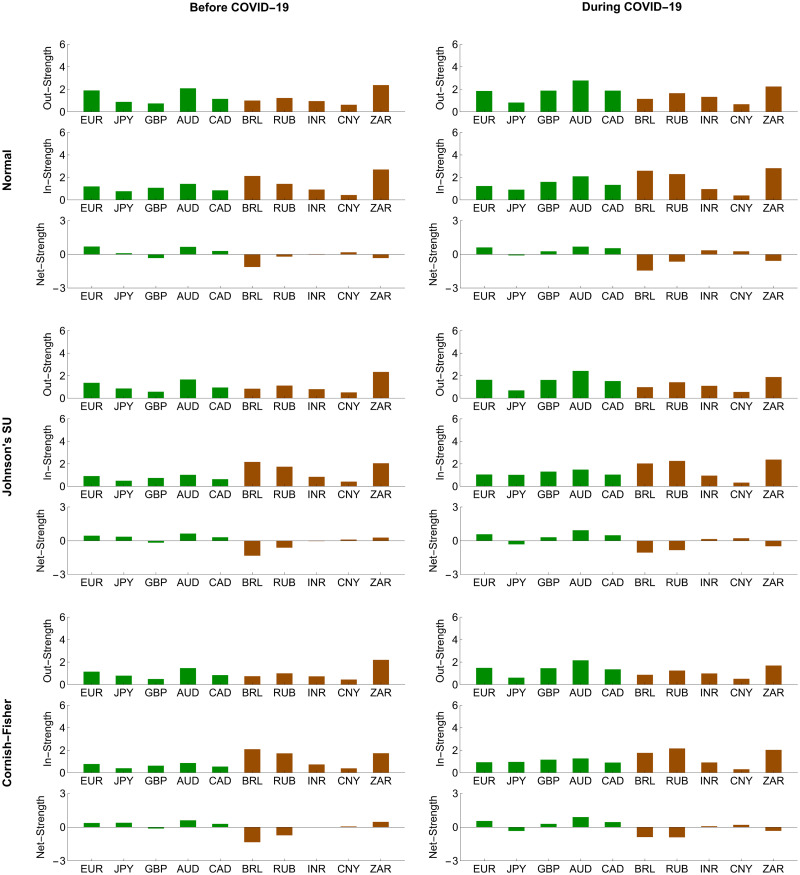
Node strength measures of the conditional tail risk networks based on ΔMCoVaR forecasts. Green and brown represent the advanced and emerging forex markets, respectively.

To further investigate the systemic importance of each forex market in the network, we assessed its topological structure by making use of out-, in-, and net-strength measures, as we formulate in Eqs (42)–(44), respectively. A forex market with the highest out-strength (respectively, in-strength) means that it serves as the most important transmitter (respectively, receiver) of systemic risk. If its net strength is highly positive (negative), it becomes the most systemically important net transmitter (net receiver). Figs 8 and 9 reveal that the magnitude of the out-, in-, and net-strength measures of the ten forex markets under study tended to increase in response to the COVID-19 pandemic. This result is in line with evidence we find from Figs 6 and 7 that they exhibited more pronounced interconnectedness because of this pandemic. In particular, the Australian and South African forex markets had the highest out-strengths during the pre-COVID-19 and COVID-19 periods, meaning that they acted as the most important transmitters of systemic risk. Meanwhile, the Japanese and Chinese forex markets were the smallest contributors to systemic risk transmission. On the basis of in-strength values, the South African forex market ranked first as the most systemically important risk receiver, and the Brazilian and Russian forex markets ranked second and third, respectively. As the COVID-19 pandemic progressed, the forex market of Russia received more impacts from the risk transmission mechanism. Its role as an important recipient of systemic risk became more apparent than that of the South African forex market. On the other hand, the forex markets of Japan and China possessed the lowest in-strengths, indicating that they suffered the least from the risk transmission mechanism. Among the aforementioned forex markets, we observed that the Australian and Brazilian forex markets played roles as the most systemically important net transmitter and net receiver, respectively. Overall, the advanced forex markets appeared to be net transmitters of systemic risk with positive net strengths. In contrast, the emerging forex markets tended to serve as net receivers of systemic risk with negative net strengths. This means that the former emitted more systemic risk contributions than they received, while the latter behaved as their exact opposite.

Evidence that the Australian forex market was the most important (net-)emitter of systemic risk is consistent with what Anwer et al. [54] uncovered from their investigation on spillovers across the Asian-Pacific forex markets during the periods of the Asian and global financial crises and COVID-19. See also Polat [52], who only gave attention to advanced forex markets with the most active trading. However, the role of the above forex market and that of the South African forex market amid the current COVID-19 crisis are contrary to their roles as the least important volatility transmitters in times of previous financial crises, as Wen and Wang [56] documented. Nevertheless, they reported evidence of the strongest bidirectional volatility spillovers between the Australian and New Zealand forex markets. Another difference between our results and those of Wen and Wang [56] lies in the capability of the forex markets of South Africa, Russia, and Brazil to receive risk and volatility spillovers from the remaining forex markets. Meanwhile, Wang and Xie [25], who employed tail dependence-based networks, indicated the smallest contribution that Japan made to the global forex network during the years 2005–2012. They argued that Japan’s forex regime keeping the yen as a weak currency might have caused this indication. Greenwood-Nimmo et al. [51] also found Japan to have the safest forex market when analyzing spillovers across the forex markets of the G10 member countries over the period from January 1999 to October 2014. This finding is in line with the notion that the Japanese yen is a safe-haven currency; see also Ranaldo and S derlind [62]. Our abovementioned results confirm that this currency remains safe in times of the current crisis due to the COVID-19 pandemic; see also our previous indication we detect from Fig 3. However, the current small influence of the Chinese yuan on the global forex market is contrary to another finding of Wang and Xie’s [25] study. More specifically, they stated that this currency had a powerful influence because of its strong lower- and upper-tail dependence on the US dollar during the analyzed period when the US subprime mortgage crisis and the global financial crisis occurred.

**Fig 10:**
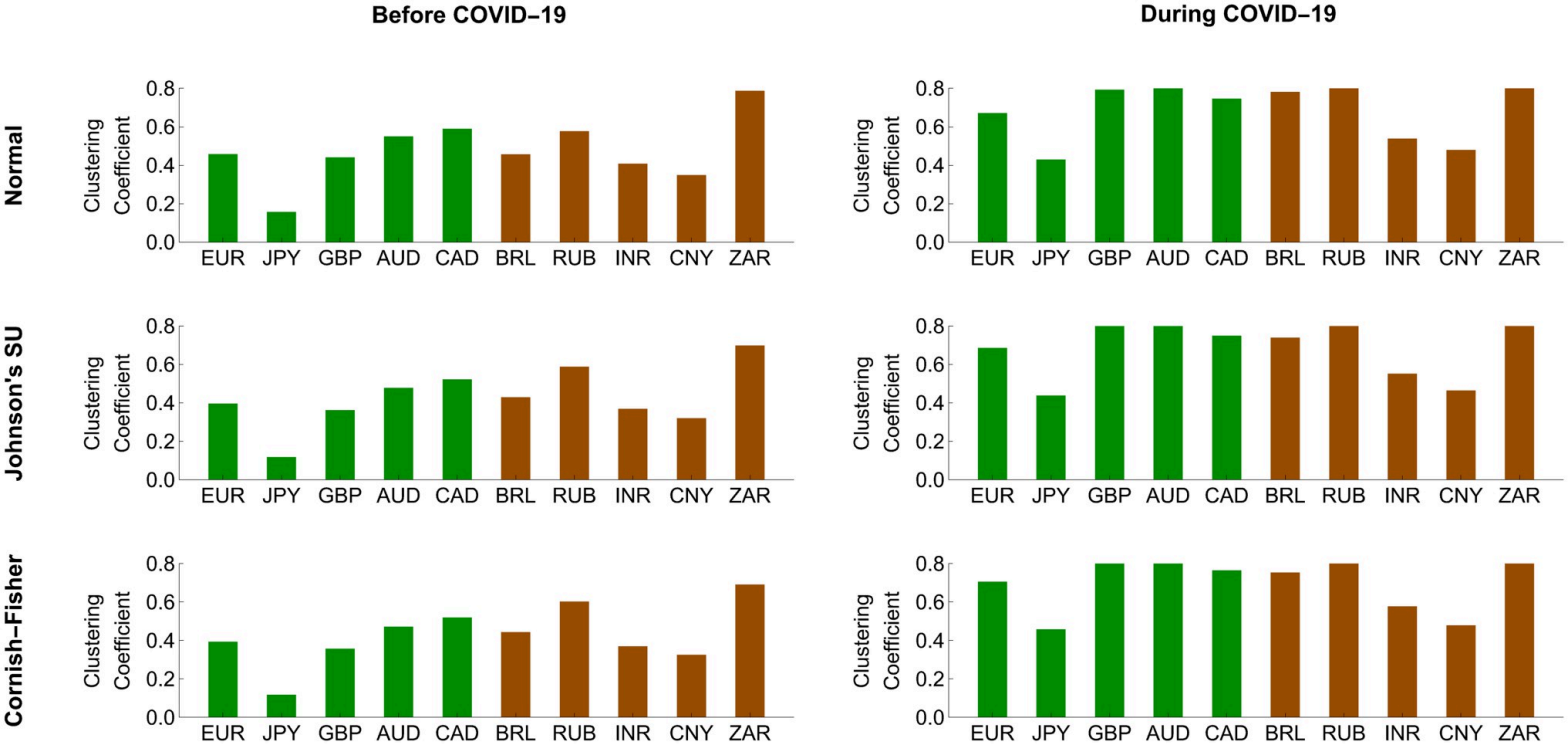
Node clustering coefficients of conditional tail risk networks based on ΔCoVaR forecasts. Green and brown represent the advanced and emerging forex markets, respectively.

**Fig 11:**
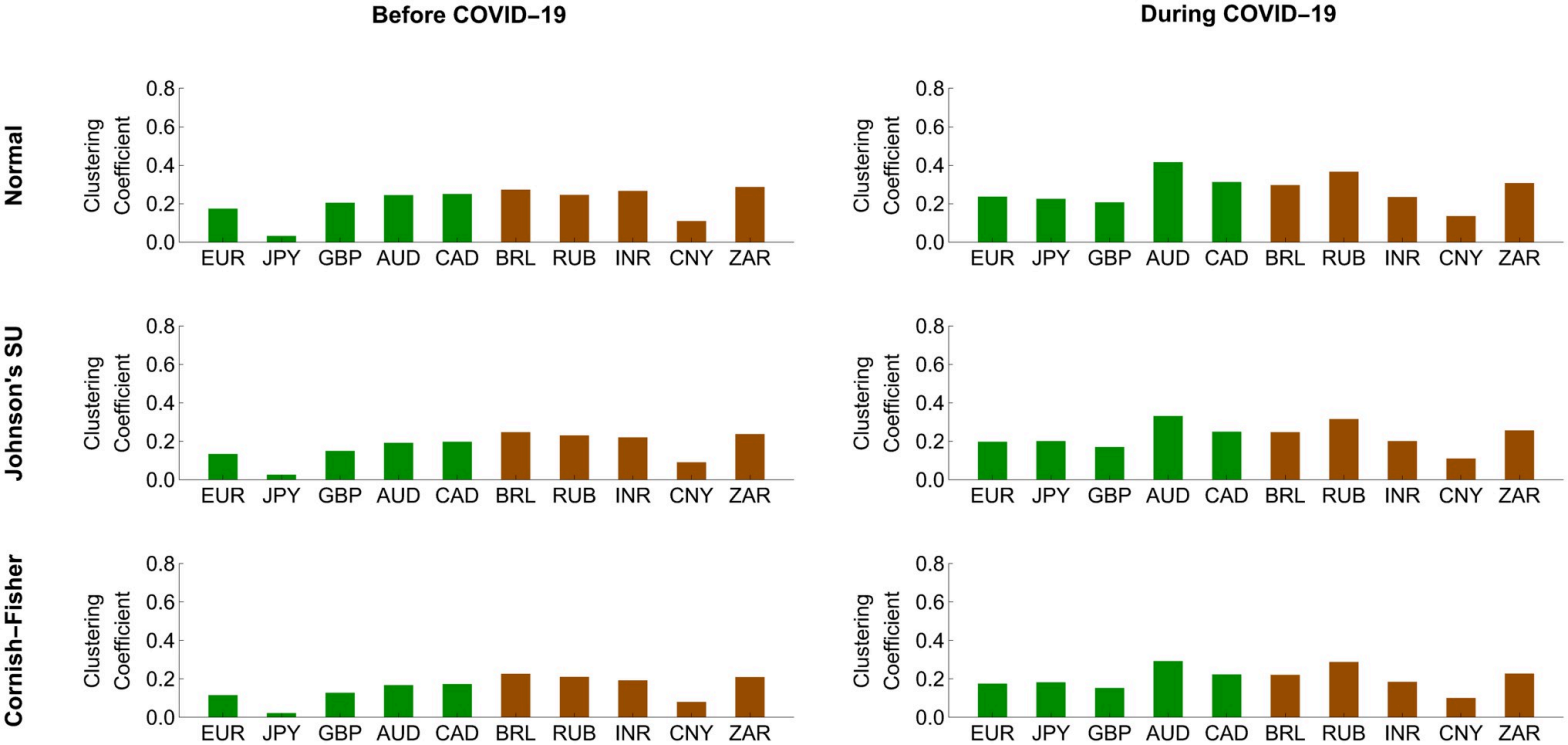
Node clustering coefficients of conditional tail risk networks based on ΔMCoVaR forecasts. Green and brown represent the advanced and emerging forex markets, respectively.

In addition, we computed the coefficient of clustering around each forex market according to Eq (45). The larger the clustering coefficient around a particular market, the higher its tendency to cluster with its neighbors in the network. As we present in Figs 10 and 11, we observe that the forex markets of several emerging countries, such as South Africa, Russia, and Brazil, had relatively high clustering coefficients. This means that they tended to make clusters with their neighbors and establish systemic risk propagation. In the face of the COVID-19 pandemic, the advanced forex markets of Australia, Canada, and the United Kingdom exhibited a substantial increase in their clustering coefficients. In general, the clustering coefficients of the ten forex markets appeared to rise due to the pandemic. As we summarize in Table 10, their average for a given network during COVID-19 also had a larger value than that before COVID-19. Wang and Xie [25] argued that the averaged clustering coefficient reflects the overall tightness of the network. Accordingly, our results indicate that the forex network amid the current COVID-19 crisis period was tighter than that during the quiet period prior to COVID-19. This indication is contrary to Wang and Xie’s [25] finding that pointed out that a network based on the dependence among the forex rate returns’ upper tails (suggesting market booms) was tighter than that based on the dependence among the forex rate returns’ lower tails (indicating market busts).

**Table 10:** Averaged node clustering coefficients of conditional tail risk networks.

	ΔCoVaR-based Network	ΔMCoVaR-based Network
Before COVID-19		
N	0.48	0.21
SU	0.43	0.17
CF	0.43	0.15
During COVID-19		
N	0.71	0.27
SU	0.71	0.23
CF	0.73	0.21

This table summarizes Figs 10 and 11 by computing the average $\bar{c}=\frac{1}{I}\sum_{i\in ℐ}c_{i}$ of the clustering coefficients of all *I* nodes of a given network, where *I* = 10.

### Robustness check

To verify the robustness of the results of this study, we reapplied the proposed methodology to the sampled forex rate returns by considering another starting point for the COVID-19 period. More specifically, we changed the predetermined starting point of March 11, 2020, to January 30, 2020, on which the WHO declared the COVID-19 outbreak a public health emergency of international concern (PHEIC). The resimulation results we provide in S1 Appendix. revealed the superior performance of the Cornish–Fisher expansion in forecasting (M)CoVaR compared to the normal and Johnson’s SU models, particularly amid the COVID-19 period. When applying the resulting Δ(M)CoVaR forecasts to build global forex networks, the interconnectedness across the ten forex markets appeared to increase due to the COVID-19 outbreak. We found that the South African and Australian (Russian) forex markets acted as the most systemically important risk transmitters (receivers) and that advanced and emerging forex markets mostly became net transmitters and net receivers of systemic risk, respectively. Furthermore, we detected that the clustering tendency of these forex markets (in particular, the former) was significantly more apparent amid the COVID-19 period. In summary, a change in the starting point of the COVID-19 period resulted in consistent findings.

## Conclusion

This study proposed a methodological framework to modify the conditional tail risk measures for systemic risk, namely, (Δ)CoVaR and (Δ)MCoVaR. In detail, we carried out the modification by first employing a multivariate extension of Johnson’s SU model and then adopting the so-called Cornish–Fisher expansion. Our motivation for relying on this approach was to make the first four analytic conditional moments appear in the explicit formulas of the above systemic risk measures. We utilized the forecasted values of the (un)expanded ΔCoVaR and ΔMCoVaR to define the direction and weight of each network edge connecting two financial entities. We then formulated several quantities to measure the topological structure of the directed and weighted conditional tail risk networks we constructed. We applied the proposed methodology to global foreign exchange (forex) markets of advanced and emerging economies over a period ranging from the beginning of 2018 to the end of 2021.

The empirical results revealed that the Cornish–Fisher expansion allowed us to derive an accurate (M)CoVaR forecast. The reason is that the expanded (M)CoVaR forecast had an estimated conditional coverage probability or (M)CoCP with the lowest RMSE in most pairs of targeted and distressing forex markets. This superior conditional coverage performance of the above expansion was more evident when we considered the sampled forex rate returns over the COVID-19 period. When paying no attention to this expansion method, Johnson’s SU model may fail to outperform the normally distributed benchmark model. This limitation may result from an increased number of its parameters being much larger than the number of the normal model’s parameters in a multivariate form. In future research, one may propose alternative methods to formulate modified (M)CoVaR forecasts with improved conditional coverage properties. In particular, one can adopt the approaches of Vidoni [68], Ueki and Fueda [69], Kabaila and Syuhada [70], and Syuhada [71], which have introduced improved versions of VaR forecasts.

Using out- and in-strengths as measures of node centrality, we identified the systemic importance of the Australian, South African, and Russian forex markets. More specifically, the former two forex markets were the most important transmitters of systemic risk before and during COVID-19. This means that the total systemic risk contributions we assigned to the edges directed from them to the remaining forex markets were very large. In addition, the forex market of South Africa also served as the most systemically important risk recipient before the COVID-19 pandemic progressed. During the pandemic, the Russian forex market played this role. When we took a net-strength measure into consideration, we found the Australian and Brazilian forex markets to be the most systemically important net transmitter and net receiver, respectively. In addition to these two forex markets, there was evidence that the other advanced and emerging forex markets generally acted as net transmitters and net receivers of systemic risk, respectively. Using a clustering coefficient, we demonstrated that the latter forex markets also exhibited a high tendency to cluster with their neighbors by forming all possible directed triangles in the constructed network. During the COVID-19 period, this tendency was more evident around the former forex markets.

Overall, the total systemic risk contributions that the considered global forex markets made tended to increase in response to the outbreak of the COVID-19 pandemic. A tighter forex network during the COVID-19 period supported evidence that the pandemic gave rise to systemic risk in these markets. This evidence suggests that financial regulators should make appropriate decisions to maintain the stability of the above markets in times of crisis, such as the COVID-19 crisis. Furthermore, they should more deeply investigate the changes in the topological structure of the global forex networks due to the current COVID-19 crisis with the aim of preventing new systemic events. As Wang and Xie [25] recommended, they may achieve this aim by constructing an early warning system for such markets from a macro perspective. In particular, this system should more closely monitor the most systemically important forex markets of Australia and South Africa from which systemic risk may arise with a high possibility. From the forex traders’ viewpoint, the different topological structures of the forex networks over different periods (i.e., a calm period or a crisis period) should also receive more attention when formulating investment and trading strategies. They should immediately adjust these strategies once the forex market situations substantially change due to crises [25]. In addition, they may add, into their portfolios, safe-haven currencies (e.g., the Japanese yen), which are able to shield their investments from extreme risks, particularly during crisis periods. On the basis of our very accurate modification framework and sophisticated networks, they may take our decision-making recommendations with enhanced confidence.

## Supporting information

S1 Data Foreign exchange rate data. (XLSX)

S1 File Data availability statement. (PDF)

S1 Appendix Resimulation results for robustness check. (PDF)

## References

[1] Choi P, Nam K. Asymmetric and leptokurtic distribution for heteroscedastic asset returns: The *S*_*U*_-normal distribution. J Empir Finance. 2008;15(1):41–63. doi: 10.1016/j.jempfin.2006.06.009

[2] Alexander C, Lazar E, Stanescu S. Forecasting VaR using analytic higher moments for GARCH processes. Int Rev Financ Anal. 2013;30:36–45. doi: 10.1016/j.irfa.2013.05.006

[3] Babikir A, Hassan ME, Mwambi H. Asymmetry, fat-tail and autoregressive conditional density in daily stocks return data. Ann Econ Stat. 2019;135:57–68. doi: 10.15609/annaeconstat2009.135.0057

[4] Johnson NL. Systems of frequency curves generated by method of translation. Biometrika. 1949;36(1–2):149–176. doi: 10.1093/biomet/36.1-2.149 18132090

[5] Barndorff-Nielsen OE, Cox DR. Asymptotic techniques for use in statistics. London: Chapman & Hall; 1989.

[6] Favre L, Galeano J-A. Mean-modified value-at-risk optimization with hedge funds. J Altern Invest. 2002;5(2):21–25. doi: 10.3905/jai.2002.319052

[7] Christoffersen P, Gon alves S. Estimation risk in financial risk management. J Risk. 2005;7(3):1–28. doi: 10.21314/JOR.2005.112

[8] Gueyié J-P, Amvella SP. Optimal portfolio allocation using funds of hedge funds. J Wealth Manag. 2006;9(2):85–95. doi: 10.3905/jwm.2006.644221

[9] Boudt K, Peterson B, Croux C. Estimation and decomposition of downside risk for portfolios with non-normal returns. J Risk. 2008;11(2):79–103. doi: 10.21314/JOR.2008.188

[10] You L, Daigler RT. Using four-moment tail risk to examine financial and commodity instrument diversification. Financ Rev. 2010;45(4):1101–1123. doi: 10.1111/j.1540-6288.2010.00287.x

[11] Bredin D, Conlon T, Potì V. The price of shelter—Downside risk reduction with precious metals. Int Rev Financ Anal. 2017;49:48–58. doi: 10.1016/j.irfa.2016.12.005

[12] Billio M, Getmansky M, Lo AW, Pelizzon L. Econometric measures of connectedness and systemic risk in the finance and insurance sectors. J Financ Econ. 2012;104(3):535–559. doi: 10.1016/j.jfineco.2011.12.010

[13] Deng Y, Zhang Z, Zhu L. A model-based index for systemic risk contribution measurement in financial networks. Econ Model. 2021;95:35–48. doi: 10.1016/j.econmod.2020.11.011

[14] Bernard C, Brechmann EC, Czado C. Statistical assessments of systemic risk measures. In: Fouque J-P, Langsam JA, editors. Handbook on systemic risk. New York: Cambridge University Press; 2013. pp. 165–179.

[15] Bekiros S, Nguyen DK, Sandoval L Jr, Uddin GS. Information diffusion, cluster formation and entropy-based network dynamics in equity and commodity markets. Eur J Oper Res. 2017;256(3):945–961. doi: 10.1016/j.ejor.2016.06.052

[16] Mantegna RN. Hierarchical structure in financial markets. Eur Phys J B. 1999;11(1):193–197. doi: 10.1007/s100510050929

[17] Onnela J-P, Kaski K, Kertész J. Clustering and information in correlation based financial networks. Eur Phys J B. 2004;38(2):353–362. doi: 10.1140/epjb/e2004-00128-7

[18] Nobi A, Lee S, Kim DH, Lee JW. Correlation and network topologies in global and local stock indices. Phys Lett A. 2014;378(34):2482–2489. doi: 10.1016/j.physleta.2014.07.009

[19] Wang G-J, Xie C, Zhang P, Han F, Chen S. Dynamics of foreign exchange networks: A time-varying copula approach. Discrete Dyn Nat Soc. 2014;2014:170921. doi: 10.1155/2014/170921

[20] Giudici P, Polinesi G. Crypto price discovery through correlation networks. Ann Oper Res. 2021;299(1–2):443–457. doi: 10.1007/s10479-019-03282-3

[21] Siudak D. A network analysis of the value migration process on the financial market. The effect of value migration network structure on stock returns. Expert Syst Appl. 2022;191:116129. doi: 10.1016/j.eswa.2021.116129

[22] Kenett DY, Tumminello M, Madi A, Gur-Gershgoren G, Mantegna RN, Ben-Jacob E. Dominating clasp of the financial sector revealed by partial correlation analysis of the stock market. PLoS ONE. 2010;5(12):e15032. doi: 10.1371/journal.pone.0015032 21188140 PMC3004792

[23] Cerchiello P, Giudici P. Conditional graphical models for systemic risk estimation. Expert Syst Appl. 2016;43:165–174. doi: 10.1016/j.eswa.2015.08.047

[24] Torri G, Giacometti R, Paterlini S. Robust and sparse banking network estimation. Eur J Oper Res. 2018;270(1):51–65. doi: 10.1016/j.ejor.2018.03.041

[25] Wang G-J, Xie C. Tail dependence structure of the foreign exchange market: A network view. Expert Syst Appl. 2016;46:164–179. doi: 10.1016/j.eswa.2015.10.037

[26] Li W, Hommel U, Paterlini S. Network topology and systemic risk: Evidence from the Euro Stoxx market. Finance Res Lett. 2018;27:105–112. doi: 10.1016/j.frl.2018.02.016

[27] Wen F, Yang X, Zhou W-X. Tail dependence networks of global stock markets. Int J Finance Econ. 2019;24(1):558–567. doi: 10.1002/ijfe.1679

[28] Wang D, Huang W-Q. Centrality-based measures of financial institutions’ systemic importance: A tail dependence network view. Phys A Stat Mech Appl. 2021;562:125345. doi: 10.1016/j.physa.2020.125345

[29] Diebold FX, Yılmaz K. On the network topology of variance decompositions: Measuring the connectedness of financial firms. J Econom. 2014;182(1):119–134. doi: 10.1016/j.jeconom.2014.04.012

[30] Schweitzer F, Fagiolo G, Sornette D, Vega-Redondo F, Vespignani A, White DR. Economic networks: The new challenges. Science. 2009;325(5939):422–425. doi: 10.1126/science.1173644 19628858

[31] Wang G-J, Xie C, He K, Stanley HE. Extreme risk spillover network: application to financial institutions. Quant Finance. 2017;17(9):1417–1433. doi: 10.1080/14697688.2016.1272762

[32] Sandoval L Jr. Structure of a global network of financial companies based on transfer entropy. Entropy. 2014;16(8):4443–4482. doi: 10.3390/e16084443

[33] Wang G-J, Yi S, Xie C, Stanley HE. Multilayer information spillover networks: measuring interconnectedness of financial institutions. Quant Finance. 2021;21(7):1163–1185. doi: 10.1080/14697688.2020.1831047

[34] Wang G-J, Chen Y-Y, Si H-B, Xie C, Chevallier J. Multilayer information spillover networks analysis of China’s financial institutions based on variance decompositions. Int Rev Econ Finance. 2021;21(7):1163–1185. 10.1016/j.iref.2021.01.005

[35] Adrian T, Brunnermeier MK. CoVaR. Am Econ Rev. 2016;106(7):1705–1741. doi: 10.1257/aer.20120555

[36] Hautsch N, Schaumburg J, Schienle M. Financial network systemic risk contributions. Rev Finance. 2015;19(2):685–738. doi: 10.1093/rof/rfu010

[37] Härdle WK, Wang W, Yu L. TENET: Tail-Event driven NETwork risk. J Econom. 2016;192(2):499–513. doi: 10.1016/j.jeconom.2016.02.013

[38] Cao Z. Systemic risk measures, banking supervision and financial stability. Doctoral Dissertation, Universit de Toulouse. 2013. Available from: https://publications.ut-capitole.fr/id/eprint/13749

[39] Bernardi M, Petrella L. Interconnected risk contributions: A heavy-tail approach to analyze U.S. financial sectors. J Risk Financ Manag. 2015;8(2):198–226. doi: 10.3390/jrfm8020198

[40] Torri G, Giacometti R, Tich T. Network tail risk estimation in the European banking system. J Econ Dyn Control. 2021;127:104125. doi: 10.1016/j.jedc.2021.104125

[41] He F, Liu Z, Chen S. Industries return and volatility spillover in Chinese stock market: An early warning signal of systemic risk. IEEE Access. 2019;7:9046–9056. doi: 10.1109/ACCESS.2018.2888522

[42] Liu X, An H, Li H, Chen Z, Feng S, Wen S. Features of spillover networks in international financial markets: Evidence from the G20 countries. Phys A Stat Mech Appl. 2017;479:265–278. doi: 10.1016/j.physa.2017.03.016

[43] Baumöhl E, Kočenda E, Lyósca S, Výrost T. Networks of volatility spillovers among stock markets. Phys A Stat Mech Appl. 2018;490:1555–1574. doi: 10.1016/j.physa.2017.08.123

[44] Zhang W, Zhuang X, Lu Y. Spatial spillover effects and risk contagion around G20 stock markets based on volatility network. North Am J Econ Finance. 2020;51:101064. doi: 10.1016/j.najef.2019.101064

[45] Zhang W, Zhuang X, Wang J, Lu Y. Connectedness and systemic risk spillovers analysis of Chinese sectors based on tail risk network. North Am J Econ Finance. 2020;54:101248. doi: 10.1016/j.najef.2020.101248

[46] Lai Y, Hu Y. A study of systemic risk of global stock markets under COVID-19 based on complex financial networks. Phys A Stat Mech Appl. 2021;566:125613. doi: 10.1016/j.physa.2020.125613

[47] Li Y, Zhuang X, Wang J, Dong Z. Analysis of the impact of COVID-19 pandemic on G20 stock markets. North Am J Econ Finance. 2021;58:101530. doi: 10.1016/j.najef.2021.101530

[48] Li W. COVID-19 and asymmetric volatility spillovers across global stock markets. North Am J Econ Finance. 2021;58:101474. doi: 10.1016/j.najef.2021.101474

[49] Gunay S, Can G. The source of financial contagion and spillovers: An evaluation of the covid-19 pandemic and the global financial crisis. PLoS ONE. 2022;17(1):e0261835. doi: 10.1371/journal.pone.0261835 35030202 PMC8759666

[50] Wang H, Yuan Y, Li Y, Wang X. Financial contagion and contagion channels in the forex market: A new approach via the dynamic mixture copula-extreme value theory. Econ Model. 2021;94:401–414. doi: 10.1016/j.econmod.2020.10.002 33071422 PMC7550255

[51] Greenwood-Nimmo M, Nguyen VH, Rafferty B. Risk and return spillovers among the G10 currencies. J Financ Mark. 2016;31:43–62. doi: 10.1016/j.finmar.2016.05.001

[52] Polat O. Systemic risk contagion in FX market: A frequency connectedness and network analysis. Bull Econ Res. 2019;71(4):585–598. doi: 10.1111/boer.12197

[53] Bouri E, Lucey B, Saeed T, Vo XV. Extreme spillovers across Asian-Pacific currencies: A quantile-based analysis. Int Rev Financ Anal. 2020;72:101605. doi: 10.1016/j.irfa.2020.101605

[54] Anwer Z, Naeem MA, Hassan MK, Karim S. Asymmetric connectedness across Asia-Pacific currencies: Evidence from time-frequency domain analysis. Finance Res Lett. 2022;47:102782. doi: 10.1016/j.frl.2022.102782

[55] Chen Y, Mo D, Xu Z. A study of interconnections and contagion among Chinese financial institutions using a ΔCoVaR network. Finance Res Lett. 2022;45:102395. doi: 10.1016/j.frl.2021.102395

[56] Wen T, Wang G-J. Volatility connectedness in global foreign exchange markets. J Multinatl Financ Manag. 2020;54:100617. doi: 10.1016/j.mulfin.2020.100617

[57] Choi P, Min I, Park K. *S*_*U*_-Δ*CoVaR*. Econ Lett. 2012;115(2):218–220. doi: 10.1016/j.econlet.2011.12.002

[58] Mauleon I, Perote J. Testing densities with financial data: an empirical comparison of the EdgeworthSargan density to the Students t. Eur J Finance. 2000;6(2):225–239. doi: 10.1080/13518470050020851

[59] Barrat A, Barthélemy M, Pastor-Satorras R, Vespignani A. The architecture of complex weighted networks. Proc Natl Acad Sci USA. 2004;101(11):3747–3752. doi: 10.1073/pnas.0400087101 15007165 PMC374315

[60] Watts DJ, Strogatz SH. Collective dynamics of ‘small-world’ networks. Nature. 1998;393:440–442. doi: 10.1038/30918 9623998

[61] Fagiolo G. Clustering in complex directed networks. Phys Rev E Stat Nonlin Soft Matter Phys. 2007;76(2):026107. doi: 10.1103/PhysRevE.76.026107 17930104

[62] Ranaldo A, Söderlind P. Safe haven currencies. Rev Finance. 2010;14,385–407.

[63] Girardi G, Ergün AT. Systemic risk measurement: Multivariate GARCH estimation of CoVaR. J Bank Finance. 2013;37(8):3169–3180. doi: 10.1016/j.jbankfin.2013.02.027

[64] Bianchi ML, De Luca G, Rivieccio G. Non-Gaussian models for CoVaR estimation. Int J Forecast. 2022. doi: 10.1016/j.ijforecast.2021.12.002

[65] Johnson NJ. Modified *t* tests and confidence intervals for asymmetrical populations. J Am Stat Assoc. 1978;73(363):536–544. doi: 10.2307/2286597

[66] Boik RJ. Accurate confidence intervals in regression analyses of non-normal data. Ann Inst Stat Math. 2008;60(1):61–83. doi: 10.1007/s10463-006-0085-1

[67] Boik RJ, Haaland B. Second-order accurate inference on simple, partial, and multiple correlations. J Mod Appl Stat Methods. 2006;5(2):283–308. doi: 10.22237/jmasm/1162353660

[68] Vidoni P. Improved prediction intervals for stochastic process models. J Time Ser Anal. 2004;25(1):137–154. doi: 10.1111/j.1467-9892.2004.00341.x

[69] Ueki M, Fueda K. Adjusting estimative prediction limits. Biometrika. 2007;94(2):509–511. doi: 10.1093/biomet/asm032

[70] Kabaila P, Syuhada K. Improved prediction limits for AR(*p*) and ARCH(*p*) processes. J Time Ser Anal. 2008;29(2):213–223. doi: 10.1111/j.1467-9892.2007.00553.x

[71] Syuhada K. The improved value-at-risk for heteroscedastic processes and their coverage probability. J Probab Stat. 2020;2020:7638517. doi: 10.1155/2020/7638517

