# Supplementary material for: Modifying (M)CoVaR and constructing tail risk networks through analytic higher-order moments: Evidence from the global forex markets
Source: PLoS One. 2022 Nov 29;17(11):e0277756. doi: 10.1371/journal.pone.0277756 (PMC9707806; doi:10.1371/journal.pone.0277756)
Supplement: S1 File — (PDF) [file pone.0277756.s002.pdf]

Modifying (M)CoVaR and constructing tail risk networks through analytic higher-order moments: Evidence from the global forex markets

Arief Hakim, A.N.M. Salman, Yeva Ashari, Khreshna Syuhada*

Faculty of Mathematics and Natural Sciences, Institut Teknologi Bandung, Bandung, Indonesia

* khreshna@itb.ac.id

Data Availability Statement

All relevant data are mentioned in the manuscript and provided in the Supporting Information "S1 Data. (XLSX)." These data were collected from Yahoo Finance (https://www.finance.yahoo.com/currencies) for the period ranging from January 1, 2018, to December 31, 2021.
