# Supplementary material for: Modifying (M)CoVaR and constructing tail risk networks through analytic higher-order moments: Evidence from the global forex markets
Source: PLoS One. 2022 Nov 29;17(11):e0277756. doi: 10.1371/journal.pone.0277756 (PMC9707806; doi:10.1371/journal.pone.0277756)
Supplement: S1 Appendix — (PDF) [file pone.0277756.s003.pdf]

Modifying (M)CoVaR and constructing tail risk networks through analytic higher-order moments: Evidence from the global forex markets

Arief Hakim, A.N.M. Salman, Yeva Ashari, Khreshna Syuhada*

Faculty of Mathematics and Natural Sciences, Institut Teknologi Bandung, Bandung, Indonesia

* khreshna@itb.ac.id

Appendix

See Figs A.1–A.10 and Tables A.1–A.9.

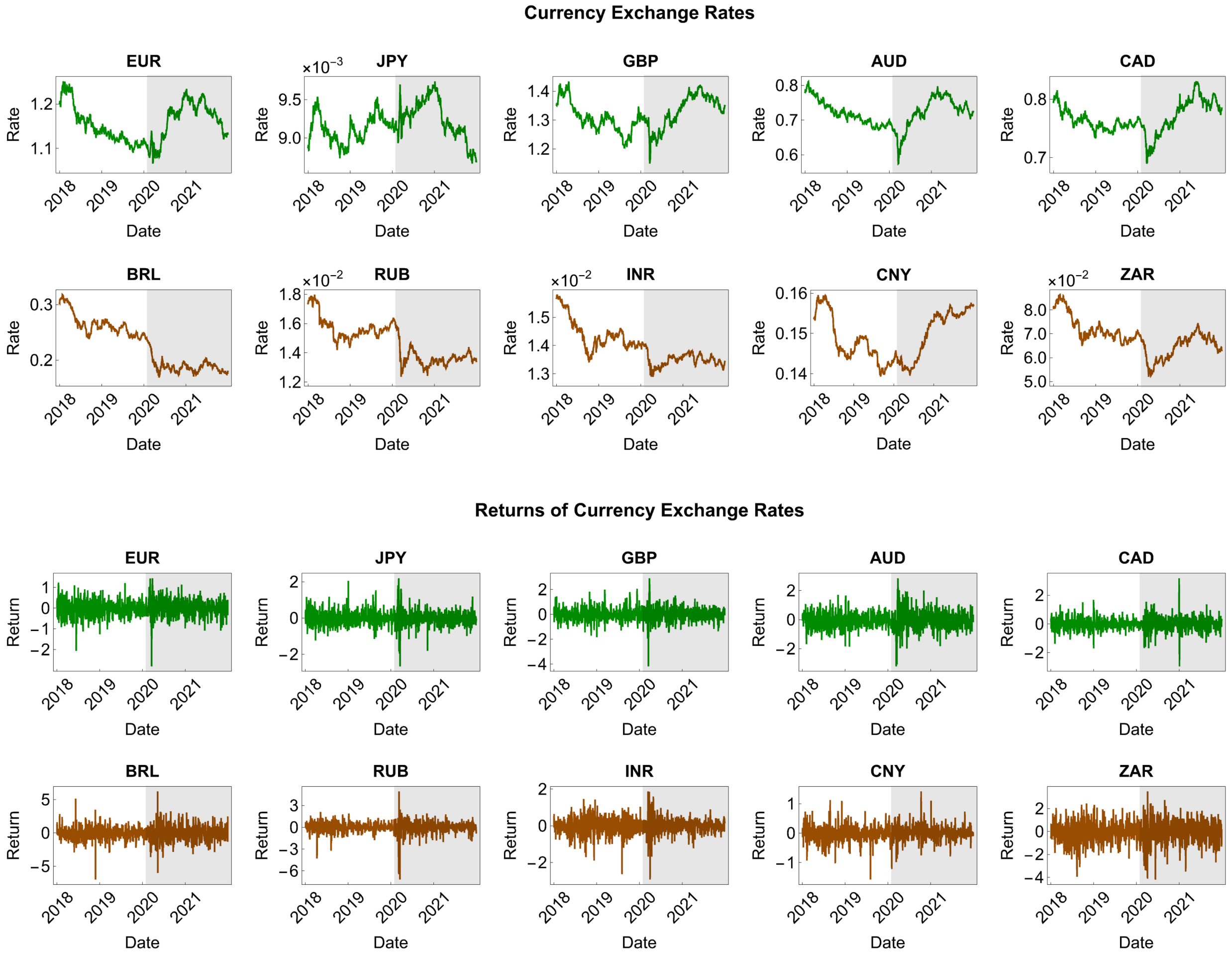

Fig A.1. Daily exchange rates of ten currencies and their returns. We denominate each currency exchange rate in US dollars. Green and brown represent the currencies of the advanced and emerging countries, respectively. Meanwhile, the shaded region represents the COVID-19 period ranging from January 30, 2020, to December 31, 2021.

Table A.1. Summary statistics of the returns of currency exchange rates.

	EUR	JPY	GBP	AUD	CAD	BRL	RUB	INR	CNY	ZAR
Before COVID-19										
Mean	−0.02	0.01	−0.01	−0.03	−0.01	−0.04	−0.01	−0.02	−0.01	−0.03
Variance	0.15	0.16	0.27	0.25	0.15	0.93	0.45	0.24	0.08	0.82
Skewness	−0.23[a]	0.51[a]	0.35[a]	−0.08	0.23[a]	−0.35[a]	−0.87[a]	−0.44[a]	−0.31[a]	−0.29[a]
Kurtosis	4.55[b]	4.71[b]	4.52[b]	3.89[b]	5.00[b]	9.42[b]	8.23[b]	4.70[b]	6.39[b]	3.65[b]
Jarque–Bera	61.27[c]	92.17[c]	65.40[c]	19.27[c]	98.87[c]	968.84[c]	704.37[c]	85.51[c]	275.68[c]	17.37[c]
During COVID-19										
Mean	0.01	−0.01	0.01	0.01	0.01	−0.06	−0.04	−0.01	0.02	−0.02
Variance	0.18	0.19	0.33	0.47	0.26	1.57	0.88	0.19	0.06	1.09
Skewness	−0.50[a]	−0.28[a]	−0.63[a]	−0.15	−0.10	0.14	−1.50[a]	−0.41[a]	−0.01	−0.48[a]
Kurtosis	6.88[b]	9.08[b]	9.76[b]	5.41[b]	8.67[b]	5.06[b]	15.26[b]	9.42[b]	7.95[b]	4.13[b]
Jarque–Bera	347.30[c]	807.30[c]	1021.83[c]	127.93[c]	695.60[c]	94.42[c]	3434.12[c]	905.08[c]	530.61[c]	47.19[c]

[a]The skewness is significantly nonzero based on the D'Agostino test at the 5% level.
[b]The kurtosis is significantly above three based on the Anscombe–Glynn test at the 5% level.
[c]The Jarque–Bera test significantly rejects the null hypothesis of normality at the 5% level.

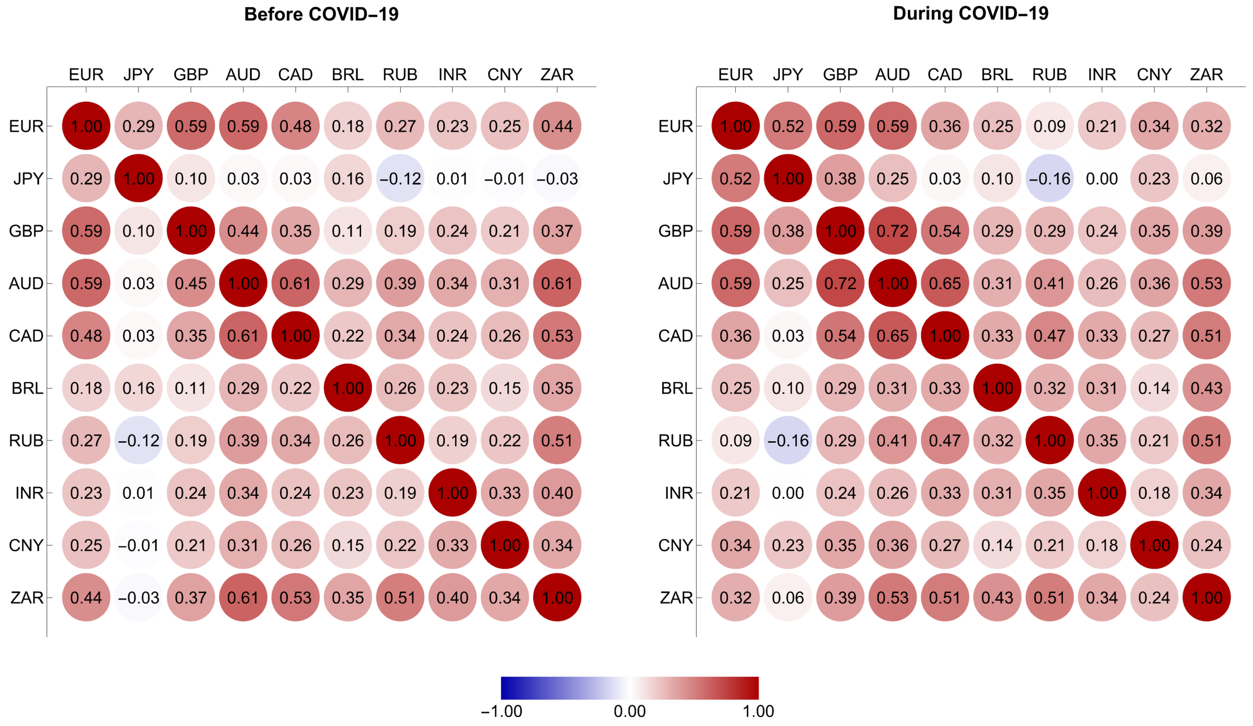

Fig A.2. Empirical correlation matrices of the returns of currency exchange rates.

Table A.2. RMSE of the estimated coverage probability $\mathrm{CP}_i^{5\%}$ of the forecasted $\mathrm{VaR}_i^{5\%}$.

	i									
	**EUR**	**JPY**	**GBP**	**AUD**	**CAD**	**BRL**	**RUB**	**INR**	**CNY**	**ZAR**
Before COVID-19										
N	0.86%	**0.73%**	**0.75%**	0.78%	**0.81%**	1.18%	1.21%	0.91%	1.01%	0.80%
SU	0.83%	0.80%	0.82%	0.79%	0.87%	**1.06%**	1.00%	0.83%	0.91%	0.78%
CF	**0.73%**	1.10%	0.91%	**0.76%**	0.88%	1.31%	**0.76%**	**0.75%**	**0.31%**	**0.78%**
During COVID-19										
N	1.10%	1.25%	1.31%	0.97%	1.18%	0.87%	1.70%	1.27%	1.12%	0.94%
SU	0.96%	**1.10%**	**1.05%**	0.92%	**1.03%**	0.91%	**1.27%**	**1.11%**	**1.00%**	0.85%
CF	**0.39%**	1.19%	1.09%	**0.59%**	1.32%	**0.86%**	3.41%	1.17%	1.15%	**0.80%**

For each forex market *i* over each period, we provide the lowest RMSE in boldface.

Table A.3. RMSE of the estimated conditional coverage probability $\mathrm{CoCP}_{j|i}^{5\%}$ of the forecasted $\mathrm{CoVaR}_{j|i}^{5\%}$ before COVID-19.

			j									
			**EUR**	**JPY**	**GBP**	**AUD**	**CAD**	**BRL**	**RUB**	**INR**	**CNY**	**ZAR**
i	EUR	N	-	**1.10%**	**1.25%**	**1.34%**	**1.30%**	**1.48%**	1.37%	1.08%	1.22%	**1.28%**
		SU	-	1.42%	1.60%	1.65%	1.66%	1.72%	1.40%	1.09%	1.02%	1.56%
		CF	-	1.42%	1.56%	1.64%	1.45%	1.67%	**1.06%**	**0.98%**	**0.81%**	1.56%
	JPY	N	**1.21%**	-	**1.01%**	1.19%	1.26%	1.59%	1.45%	1.14%	1.17%	1.15%
		SU	1.48%	-	1.08%	1.15%	1.25%	1.70%	1.16%	1.03%	1.04%	1.06%
		CF	1.31%	-	1.07%	**1.06%**	**1.09%**	**1.58%**	**0.28%**	**0.86%**	**0.35%**	**1.02%**
	GBP	N	**1.45%**	**1.06%**	-	**1.27%**	1.20%	1.43%	1.39%	1.23%	1.32%	**1.20%**
		SU	2.33%	1.37%	-	1.40%	1.39%	1.49%	1.35%	1.32%	1.24%	1.32%
		CF	2.13%	1.64%	-	1.34%	**1.16%**	**1.41%**	**1.05%**	**1.15%**	**0.77%**	1.29%
	AUD	N	**1.50%**	**1.07%**	**1.22%**	-	**1.40%**	**1.53%**	1.48%	**1.26%**	1.41%	**1.35%**
		SU	2.47%	1.47%	1.44%	-	1.87%	1.96%	1.63%	1.50%	1.38%	1.74%
		CF	2.26%	1.94%	1.35%	-	1.68%	1.78%	**0.95%**	1.30%	**0.85%**	1.73%
	CAD	N	**1.42%**	**1.14%**	**1.10%**	**1.35%**	-	**1.55%**	1.31%	1.20%	1.37%	**1.31%**
		SU	2.10%	1.22%	1.19%	1.61%	-	1.80%	1.40%	1.32%	1.29%	1.62%
		CF	1.88%	1.44%	1.11%	1.61%	-	1.69%	**0.99%**	**1.15%**	**0.82%**	1.62%
	BRL	N	**1.34%**	1.19%	1.02%	**1.14%**	1.15%	-	1.64%	1.32%	1.48%	**1.23%**
		SU	1.53%	**1.19%**	1.04%	1.17%	1.35%	-	1.70%	1.48%	1.44%	1.42%
		CF	1.36%	1.21%	**1.01%**	1.16%	**1.12%**	-	**1.02%**	**1.30%**	**0.75%**	1.48%
	RUB	N	**1.28%**	**1.14%**	1.10%	**1.24%**	1.31%	**1.65%**	-	1.23%	1.47%	**1.30%**
		SU	1.54%	1.33%	1.11%	1.28%	1.45%	2.07%	-	1.32%	1.41%	1.62%
		CF	1.38%	1.86%	**1.02%**	1.24%	**1.21%**	1.88%	-	**1.16%**	**0.83%**	1.67%
	INR	N	**1.33%**	**1.11%**	1.12%	**1.10%**	1.13%	**1.49%**	1.45%	-	1.72%	**1.22%**
		SU	1.60%	1.25%	1.21%	1.16%	1.27%	1.81%	1.41%	-	1.91%	1.40%
		CF	1.40%	1.56%	**1.11%**	1.11%	**1.02%**	1.80%	**1.07%**	-	**0.99%**	1.40%
	CNY	N	**1.29%**	**1.28%**	1.09%	1.09%	1.10%	**1.31%**	1.33%	**1.58%**	-	**1.20%**
		SU	1.54%	1.36%	1.11%	1.12%	1.26%	1.49%	1.33%	1.95%	-	1.37%
		CF	1.36%	1.64%	**1.02%**	**1.08%**	**1.03%**	1.52%	**1.07%**	1.70%	-	1.37%
	ZAR	N	**1.35%**	**1.13%**	**1.16%**	**1.33%**	**1.36%**	**1.56%**	1.76%	**1.39%**	1.47%	-
		SU	1.99%	1.37%	1.27%	1.57%	1.77%	2.18%	2.11%	1.75%	1.52%	-
		CF	1.77%	1.78%	1.17%	1.52%	1.53%	1.75%	**0.96%**	1.50%	**0.88%**	-

Forex market *i* we present in each row acts as a risk transmitter, while forex market *j* in each column serves as a risk receiver. For each pair of different forex markets *i* and *j*, we provide the lowest RMSE in boldface.

Table A.4. RMSE of the estimated conditional coverage probability CoCP$^{5\%}_{j|i}$ of the forecasted CoVaR$^{5\%}_{j|i}$ during COVID-19.

			j									
			**EUR**	**JPY**	**GBP**	**AUD**	**CAD**	**BRL**	**RUB**	**INR**	**CNY**	**ZAR**
i	EUR	N	-	2.32%	2.21%	1.66%	1.41%	1.21%	1.92%	1.79%	1.73%	1.32%
		SU	-	2.44%	2.93%	1.94%	1.51%	1.44%	**1.79%**	1.82%	1.62%	1.29%
		CF	-	**1.00%**	**0.97%**	**1.57%**	**1.17%**	**1.17%**	4.53%	**1.71%**	**0.96%**	**1.21%**
	JPY	N	**2.04%**	-	2.08%	1.54%	1.54%	1.21%	2.23%	1.67%	1.65%	1.43%
		SU	3.55%	-	2.33%	1.58%	1.42%	1.40%	**2.20%**	1.64%	1.53%	1.33%
		CF	2.19%	-	**1.72%**	**1.12%**	**1.04%**	**1.15%**	3.14%	**1.32%**	**0.80%**	**1.21%**
	GBP	N	**1.87%**	2.04%	-	**1.92%**	1.85%	**1.30%**	2.86%	1.86%	1.77%	**1.47%**
		SU	3.53%	1.97%	-	2.58%	1.82%	1.60%	**2.84%**	1.94%	1.71%	1.53%
		CF	2.41%	**1.36%**	-	2.77%	**0.88%**	1.34%	5.60%	**1.72%**	**0.95%**	1.52%
	AUD	N	**1.85%**	1.93%	2.67%	-	2.17%	1.34%	2.65%	1.87%	1.73%	1.53%
		SU	3.51%	1.90%	3.82%	-	2.19%	1.61%	**2.63%**	2.02%	1.61%	1.58%
		CF	2.25%	**1.49%**	**2.43%**	-	**1.11%**	**1.31%**	3.97%	**1.72%**	**0.96%**	**1.52%**
	CAD	N	1.68%	1.55%	2.23%	**1.81%**	-	**1.32%**	2.78%	2.00%	1.58%	**1.54%**
		SU	2.42%	1.61%	2.85%	2.24%	-	1.64%	**2.68%**	2.02%	1.47%	1.62%
		CF	**1.10%**	**1.16%**	**1.02%**	2.26%	-	1.42%	3.07%	**1.60%**	**0.88%**	1.73%
	BRL	N	1.35%	1.51%	**1.78%**	1.33%	1.52%	-	2.26%	1.81%	1.33%	1.39%
		SU	1.79%	1.40%	1.98%	1.44%	1.43%	-	**1.97%**	1.86%	1.18%	1.36%
		CF	**0.89%**	**1.10%**	1.94%	**1.05%**	**1.17%**	-	4.41%	**1.63%**	**0.47%**	**1.27%**
	RUB	N	1.41%	**1.78%**	2.77%	1.78%	1.88%	**1.29%**	-	1.83%	1.49%	**1.55%**
		SU	1.53%	2.22%	3.09%	2.21%	1.96%	1.59%	-	1.94%	1.43%	1.75%
		CF	**0.77%**	4.14%	**2.19%**	**1.70%**	**1.00%**	1.36%	-	**1.65%**	**0.85%**	1.87%
	INR	N	1.50%	1.53%	2.10%	1.67%	1.69%	**1.43%**	2.54%	-	1.51%	1.43%
		SU	1.81%	1.47%	2.15%	1.77%	1.59%	1.74%	**2.30%**	-	1.39%	1.39%
		CF	**0.87%**	**1.24%**	**2.03%**	**1.24%**	**1.18%**	1.45%	4.48%	-	**0.67%**	**1.33%**
	CNY	N	1.81%	1.89%	2.06%	1.63%	1.50%	1.32%	2.28%	1.71%	-	1.32%
		SU	2.55%	1.85%	2.32%	1.81%	1.49%	1.43%	**2.04%**	1.73%	-	1.25%
		CF	**1.12%**	**1.42%**	**1.81%**	**1.34%**	**1.15%**	**1.11%**	4.98%	**1.59%**	-	**1.17%**
	ZAR	N	1.60%	1.53%	2.09%	1.62%	1.93%	**1.34%**	2.88%	2.00%	1.49%	-
		SU	2.30%	1.72%	2.57%	1.95%	1.87%	1.76%	2.76%	2.11%	1.35%	-
		CF	**0.98%**	**1.17%**	**1.85%**	**1.46%**	**0.91%**	1.47%	**2.72%**	**1.71%**	**0.91%**	-

Forex market i we present in each row acts as a risk transmitter, while forex market j in each column serves as a risk receiver. For each pair of different forex markets i and j, we provide the lowest RMSE in boldface.

Table A.5. Number of $\text{CoVaR}_{j|\cdot}^{5\%}$ models with the best conditional coverage property.

	j									
	**EUR**	**JPY**	**GBP**	**AUD**	**CAD**	**BRL**	**RUB**	**INR**	**CNY**	**ZAR**
Before COVID-19										
N	9	8	5	7	3	7	0	3	0	8
SU	0	1	0	0	0	0	0	0	0	0
CF	0	0	4	2	6	2	9	6	9	1
During COVID-19										
N	3	1	1	2	0	5	0	0	0	3
SU	0	0	0	0	0	0	8	0	0	0
CF	6	8	8	7	9	4	1	9	9	6

This table summarizes Tables A.3 and A.4 by counting the number of the best $\text{CoVaR}_{j|\cdot}^{5\%}$ models (i.e., the normal model, Johnson's SU model, and Cornish–Fisher expansion) for each targeted market j, whose estimated conditional coverage probability $\text{CoCP}_{j|\cdot}^{5\%}$ has the lowest RMSE.

Table A.6. RMSE of the estimated conditional coverage probability $\mathrm{MCoCP}^{5\%}_{j|i,\backslash ij}$ of the forecasted $\mathrm{MCoVaR}^{5\%}_{j|i,\backslash ij}$ before COVID-19.

			j									
			**EUR**	**JPY**	**GBP**	**AUD**	**CAD**	**BRL**	**RUB**	**INR**	**CNY**	**ZAR**
i	EUR	N	-	**1.55%**	**1.43%**	**1.90%**	1.62%	1.83%	1.97%	**1.47%**	1.67%	1.78%
		SU	-	1.90%	1.85%	1.99%	1.64%	1.58%	1.67%	1.63%	1.67%	1.69%
		CF	-	1.85%	1.81%	1.95%	**1.54%**	**1.37%**	**1.08%**	1.58%	**1.19%**	**1.67%**
	JPY	N	1.45%	-	**1.28%**	**1.42%**	1.67%	1.96%	1.98%	1.29%	1.47%	**1.35%**
		SU	1.29%	-	1.41%	1.53%	1.57%	1.98%	1.46%	1.19%	1.40%	1.38%
		CF	**1.26%**	-	1.39%	1.54%	**1.51%**	**0.77%**	**0.93%**	**1.09%**	**0.91%**	1.41%
	GBP	N	1.67%	**1.22%**	-	1.48%	1.34%	1.65%	2.20%	1.49%	1.45%	1.36%
		SU	1.61%	1.40%	-	1.44%	1.33%	1.25%	1.75%	1.38%	1.38%	**1.35%**
		CF	**1.54%**	1.56%	-	**1.41%**	**1.25%**	**0.76%**	**1.12%**	**1.24%**	**0.86%**	1.36%
	AUD	N	1.98%	**1.57%**	1.67%	-	**1.71%**	1.80%	2.06%	1.49%	1.64%	1.53%
		SU	1.84%	1.88%	1.71%	-	1.91%	1.66%	1.72%	1.37%	1.52%	1.50%
		CF	**1.76%**	2.19%	**1.65%**	-	1.78%	**0.60%**	**1.11%**	**1.25%**	**0.99%**	**1.49%**
	CAD	N	1.69%	1.78%	**1.25%**	**1.65%**	-	1.86%	1.44%	1.33%	1.56%	2.01%
		SU	1.40%	1.57%	1.35%	1.70%	-	1.44%	1.24%	1.25%	1.43%	1.99%
		CF	**1.35%**	**1.55%**	1.31%	1.67%	-	**0.31%**	**0.91%**	**1.14%**	**0.92%**	**1.98%**
	BRL	N	1.58%	1.58%	**1.21%**	1.35%	1.20%	-	2.26%	1.28%	1.51%	1.35%
		SU	**1.36%**	1.41%	1.27%	1.35%	1.20%	-	1.91%	1.21%	1.40%	1.31%
		CF	1.37%	**1.31%**	1.25%	**1.33%**	**1.11%**	-	**1.17%**	**1.09%**	**0.88%**	**1.30%**
	RUB	N	1.35%	1.39%	**1.33%**	**1.39%**	1.54%	2.00%	-	1.21%	1.54%	1.34%
		SU	**1.32%**	**1.35%**	1.38%	1.42%	1.56%	1.87%	-	1.14%	1.40%	1.31%
		CF	1.40%	1.48%	1.36%	1.40%	**1.48%**	**0.61%**	-	**1.06%**	**0.89%**	**1.28%**
	INR	N	1.42%	1.21%	**1.35%**	1.29%	1.41%	1.77%	2.00%	-	1.99%	1.47%
		SU	**1.34%**	**1.18%**	1.42%	1.29%	1.33%	1.54%	1.53%	-	2.06%	1.45%
		CF	1.39%	1.25%	1.38%	**1.27%**	**1.27%**	**0.47%**	**0.96%**	-	**1.29%**	**1.45%**
	CNY	N	1.45%	1.51%	**1.26%**	1.36%	1.32%	1.42%	1.76%	1.76%	-	1.35%
		SU	**1.39%**	**1.39%**	1.32%	1.37%	1.29%	1.18%	1.38%	1.79%	-	1.29%
		CF	1.44%	1.41%	1.28%	**1.35%**	**1.20%**	**0.27%**	**0.90%**	**1.59%**	-	**1.29%**
	ZAR	N	1.75%	**1.53%**	1.42%	1.59%	2.33%	1.68%	2.55%	1.63%	1.55%	-
		SU	**1.75%**	1.56%	1.47%	1.59%	2.27%	1.87%	2.61%	1.64%	1.54%	-
		CF	1.84%	1.69%	**1.42%**	**1.56%**	**2.13%**	**0.81%**	**1.38%**	**1.46%**	**1.02%**	-

Forex market i we present in each row acts as a risk transmitter, while forex market j in each column serves as a risk receiver. For each pair of different forex markets i and j, we provide the lowest RMSE in boldface.

Table A.7. RMSE of the estimated conditional coverage probability $\mathrm{MCoCP}_{j|i,\setminus ij}^{5\%}$ of the forecasted $\mathrm{MCoVaR}_{j|i,\setminus ij}^{5\%}$ during COVID-19.

			j									
			**EUR**	**JPY**	**GBP**	**AUD**	**CAD**	**BRL**	**RUB**	**INR**	**CNY**	**ZAR**
i	EUR	N	-	2.37%	**1.76%**	2.25%	2.19%	1.58%	**1.96%**	2.49%	2.05%	1.97%
		SU	-	2.86%	2.02%	2.25%	2.53%	1.65%	3.68%	2.33%	2.01%	2.02%
		CF	-	**1.65%**	3.06%	**2.24%**	**2.05%**	**1.46%**	5.34%	**0.81%**	**0.83%**	**1.97%**
	JPY	N	2.27%	-	2.61%	**2.03%**	2.88%	1.68%	**1.95%**	1.77%	1.57%	1.53%
		SU	2.06%	-	1.85%	2.29%	2.36%	1.85%	2.16%	1.80%	1.42%	1.51%
		CF	**1.68%**	-	**1.63%**	2.41%	**2.31%**	**1.63%**	2.32%	**0.93%**	**0.53%**	**1.47%**
	GBP	N	**2.04%**	2.43%	-	2.83%	2.87%	1.72%	3.98%	2.14%	2.21%	**1.92%**
		SU	2.15%	2.90%	-	2.92%	2.10%	1.76%	3.52%	2.13%	2.27%	2.11%
		CF	2.41%	**1.81%**	-	**2.81%**	**1.69%**	**1.56%**	**2.21%**	**0.79%**	**1.01%**	2.07%
	AUD	N	2.55%	2.59%	3.22%	-	2.92%	2.14%	2.34%	2.62%	2.75%	1.93%
		SU	2.56%	2.83%	2.87%	-	2.65%	2.11%	2.20%	2.68%	2.89%	1.91%
		CF	**2.45%**	**2.43%**	**2.22%**	-	**2.01%**	**1.99%**	**1.16%**	**2.30%**	**1.58%**	**1.84%**
	CAD	N	**2.57%**	**2.45%**	**1.82%**	1.88%	-	1.48%	2.37%	2.53%	1.77%	1.96%
		SU	2.61%	2.58%	1.96%	1.92%	-	1.60%	2.21%	2.41%	1.73%	2.00%
		CF	2.95%	2.67%	2.77%	**1.87%**	-	**1.40%**	**1.63%**	**0.89%**	**0.81%**	**1.93%**
	BRL	N	**1.54%**	1.75%	**1.56%**	**1.90%**	2.06%	-	1.99%	1.93%	1.38%	1.64%
		SU	1.68%	1.79%	1.71%	2.08%	1.49%	-	1.70%	1.92%	1.26%	1.61%
		CF	2.21%	**1.20%**	3.06%	2.20%	**1.10%**	-	**1.53%**	**0.72%**	**0.89%**	**1.55%**
	RUB	N	**1.94%**	**1.74%**	3.35%	**2.03%**	2.68%	1.48%	-	1.76%	1.55%	1.75%
		SU	3.22%	2.27%	2.18%	2.07%	2.22%	1.61%	-	1.79%	1.48%	1.81%
		CF	4.27%	2.43%	**1.43%**	2.08%	**1.78%**	**1.41%**	-	**0.80%**	**0.56%**	**1.74%**
	INR	N	1.83%	1.51%	1.93%	**1.64%**	2.66%	1.63%	2.20%	-	1.58%	**1.77%**
		SU	**1.71%**	1.61%	**1.60%**	1.79%	2.11%	1.62%	1.63%	-	1.50%	1.84%
		CF	1.97%	**1.14%**	2.64%	1.92%	**1.70%**	**1.42%**	**0.97%**	-	**0.56%**	1.81%
	CNY	N	1.81%	1.52%	1.95%	**2.27%**	2.17%	1.40%	1.98%	1.87%	-	1.51%
		SU	**1.40%**	1.50%	**1.83%**	2.31%	1.64%	1.31%	1.37%	1.75%	-	1.49%
		CF	1.53%	**0.97%**	2.83%	2.30%	**1.21%**	**1.18%**	**1.05%**	**0.43%**	-	**1.45%**
	ZAR	N	2.01%	1.67%	**1.95%**	**1.83%**	3.24%	1.45%	2.68%	2.55%	1.61%	-
		SU	**1.71%**	1.76%	2.24%	1.91%	2.46%	1.56%	2.01%	2.40%	1.54%	-
		CF	1.96%	**1.16%**	3.77%	1.91%	**1.98%**	**1.39%**	**0.99%**	**0.79%**	**0.66%**	-

Forex market i we present in each row acts as a risk transmitter, while forex market j in each column serves as a risk receiver. For each pair of different forex markets i and j, we provide the lowest RMSE in boldface.

Table A.8. Number of $\text{MCoVaR}^{5\%}_{j|\cdot}$ models with the best conditional coverage property.

	j									
	**EUR**	**JPY**	**GBP**	**AUD**	**CAD**	**BRL**	**RUB**	**INR**	**CNY**	**ZAR**
Before COVID-19										
N	0	4	7	4	1	0	0	1	0	1
SU	5	3	0	0	0	0	0	0	0	1
CF	4	2	2	5	8	9	9	8	9	7
During COVID-19										
N	4	2	4	6	0	0	2	0	0	2
SU	3	0	2	0	0	0	0	0	0	0
CF	2	7	3	3	9	9	7	9	9	7

This table summarizes Tables A.6 and A.7 by counting the number of the best $\text{MCoVaR}^{5\%}_{j|\cdot}$ models (i.e., the normal model, Johnson's SU model, and Cornish–Fisher expansion) for each targeted forex market j, whose estimated conditional coverage probability $\text{MCoCP}^{5\%}_{j|\cdot}$ has the lowest RMSE.

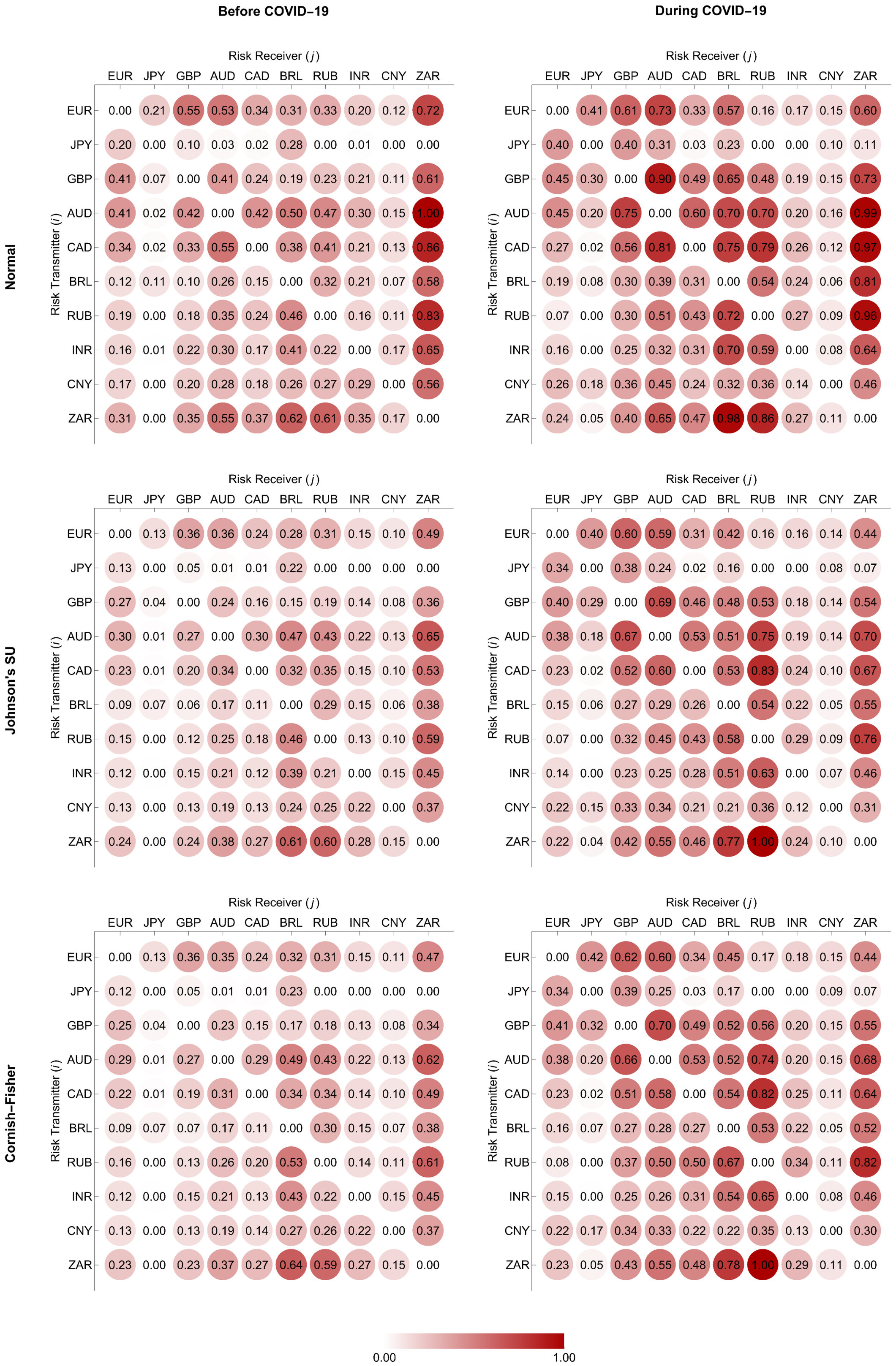

Fig A.3. Normalized weighting matrices based on ΔCoVaR forecasts.

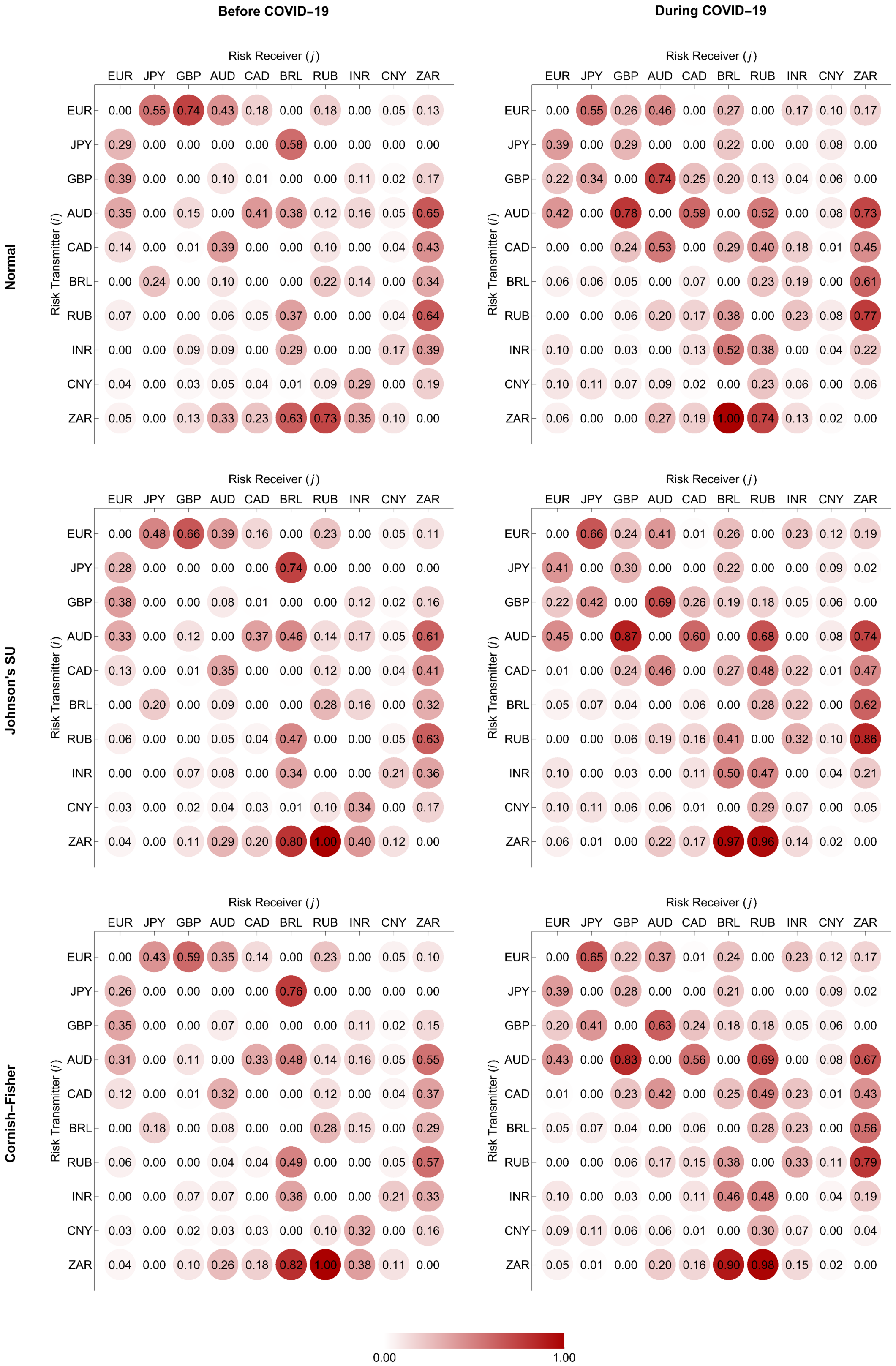

Fig A.4. Normalized weighting matrices based on ΔMCoVaR forecasts.

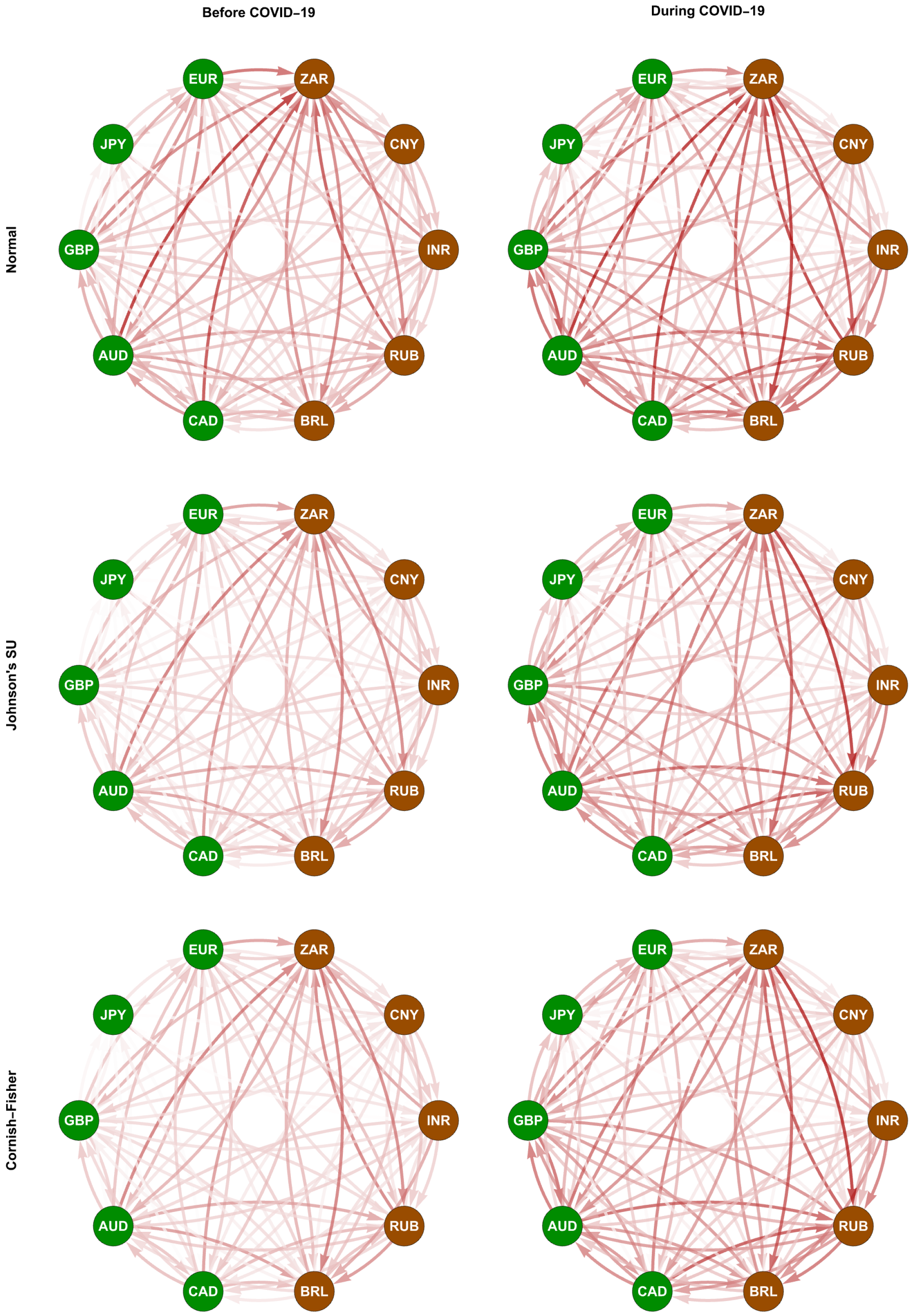

Fig A.5. Conditional tail risk networks based on ΔCoVaR forecasts. The green and brown nodes represent the advanced and emerging forex markets, respectively. The darkness of each edge indicates the magnitude of its weight, as we present in Fig A.3.

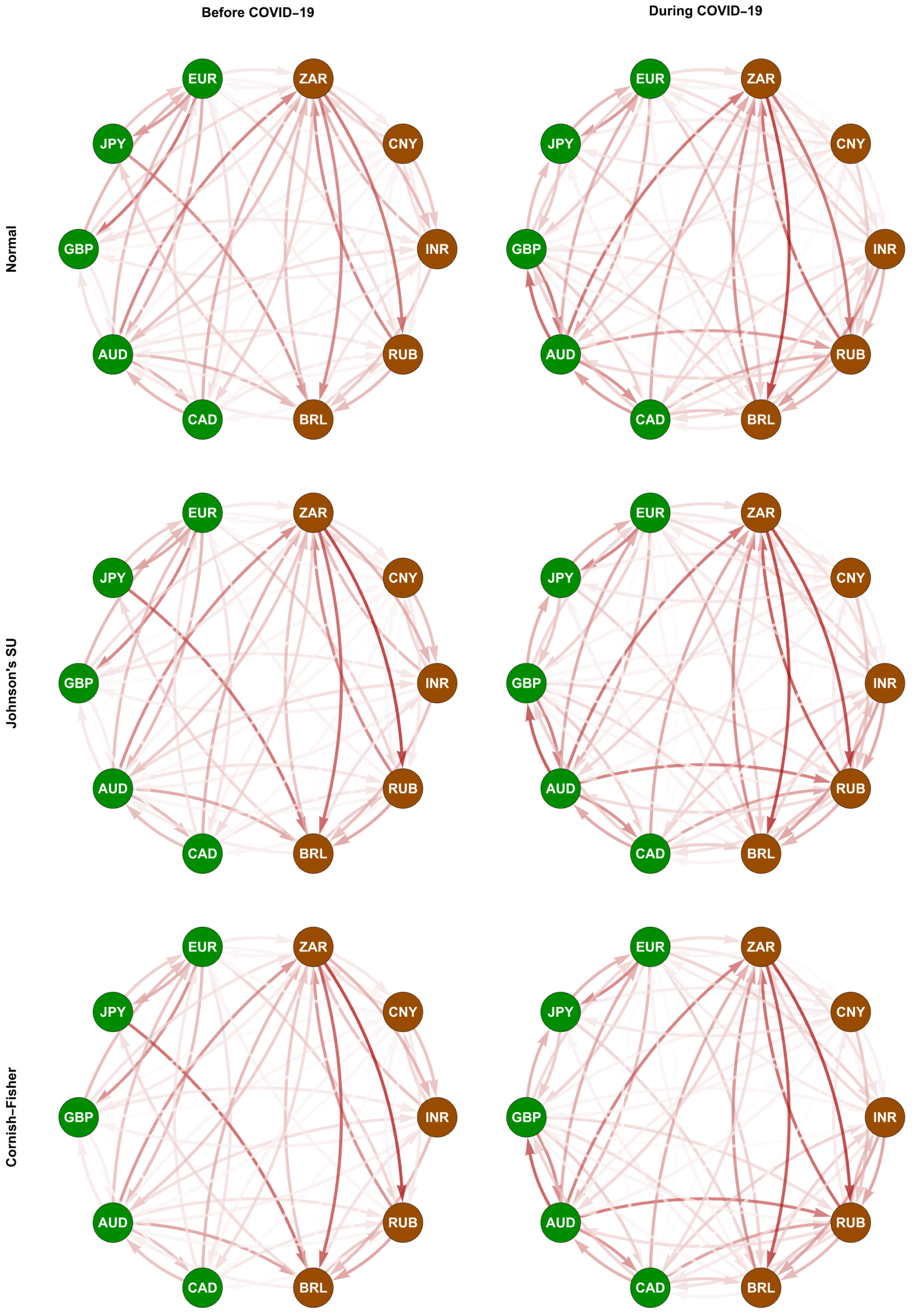

Fig A.6. Conditional tail risk networks based on ΔMCoVaR forecasts. The green and brown nodes represent the advanced and emerging forex markets, respectively. The darkness of each edge indicates the magnitude of its weight, as we present in Fig A.4.

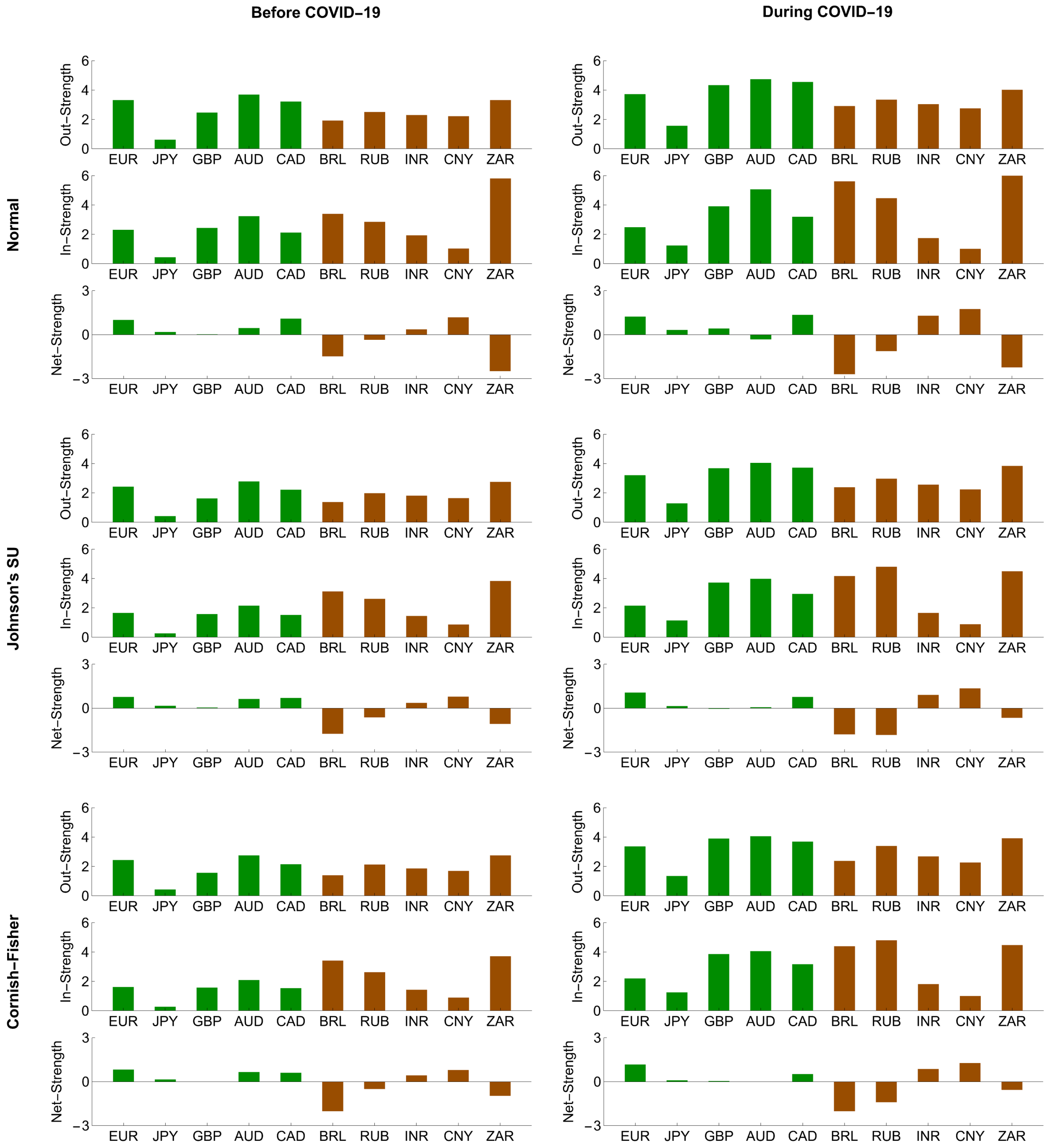

Fig A.7. Node strength measures of conditional tail risk networks based on ΔCoVaR forecasts. Green and brown represent the advanced and emerging forex markets, respectively.

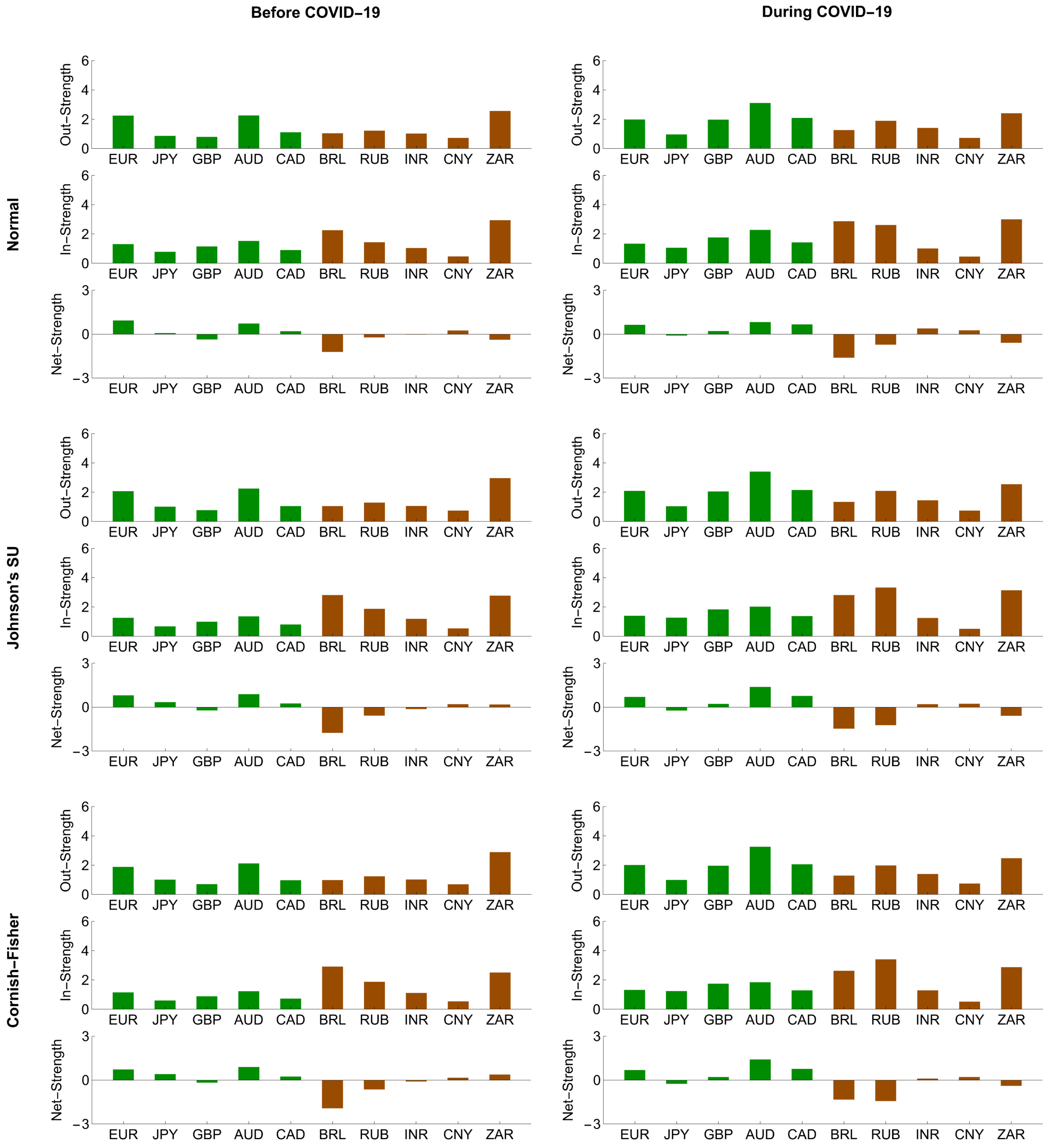

Fig A.8. Node strength measures of conditional tail risk networks based on ΔMCoVaR forecasts. Green and brown represent the advanced and emerging forex markets, respectively.

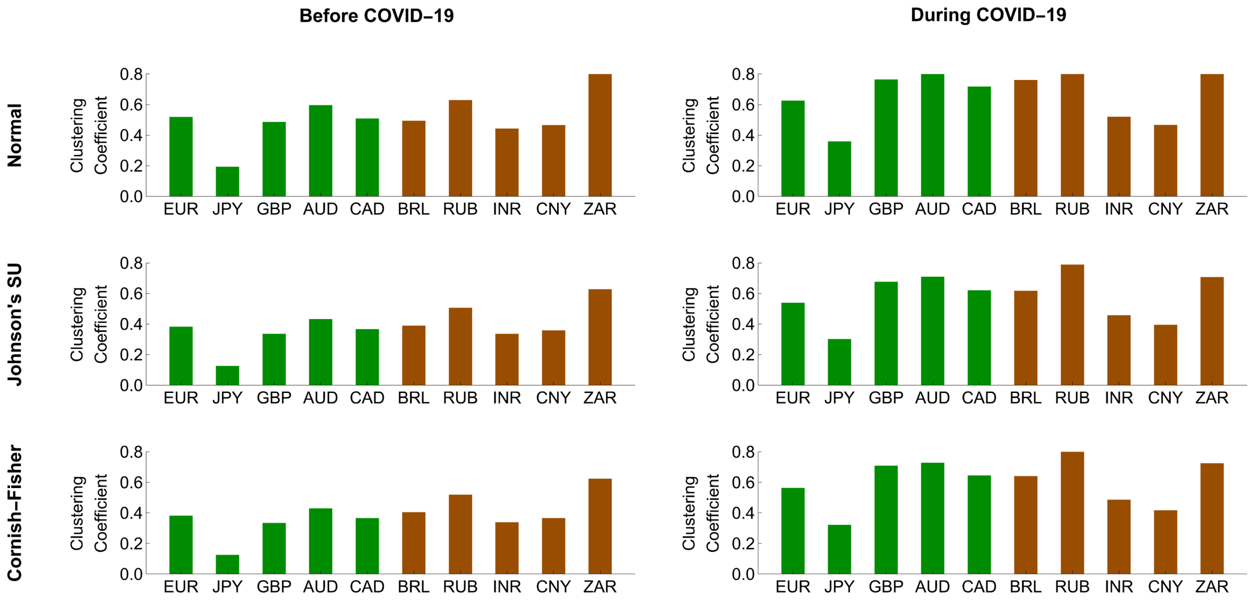

Fig A.9. Node clustering coefficients of conditional tail risk networks based on ΔCoVaR forecasts. Green and brown represent the advanced and emerging forex markets, respectively.

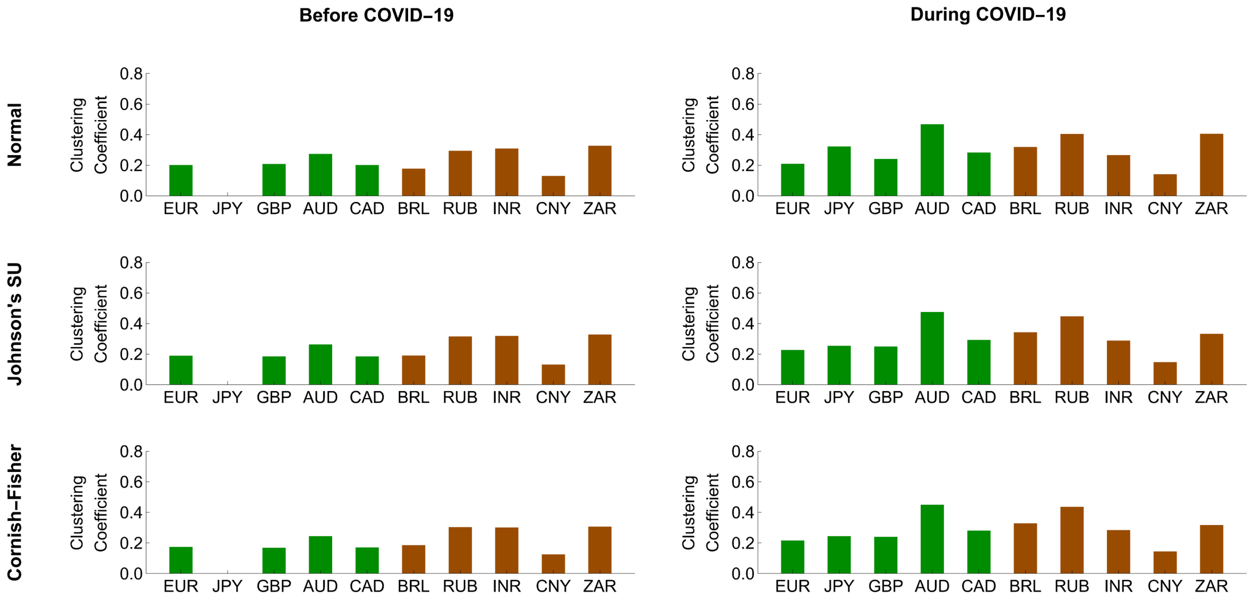

Fig A.10. Node clustering coefficients of conditional tail risk networks based on ΔMCoVaR forecasts. Green and brown represent the advanced and emerging forex markets, respectively.

Table A.9. Averaged node clustering coefficients of conditional tail risk networks.

	**ΔCoVaR-based Network**	**ΔMCoVaR-based Network**
Before COVID-19		
N	0.52	0.21
SU	0.39	0.21
CF	0.39	0.20
During COVID-19		
N	0.68	0.31
SU	0.58	0.31
CF	0.61	0.29

This table summarizes Figs A.9 and A.10 by computing the average of the clustering coefficients of all the nodes of a given network.
